# Extralevator Abdominoperineal Excision Improves Overall Survival Compared to Standard Abdominoperineal Excision: A Systematic Review and Meta‐Analysis

**DOI:** 10.1002/ags3.70182

**Published:** 2026-01-25

**Authors:** Sarolta Beáta Kávási, Diana‐Elena Floria, Anett Rancz, Dániel Sándor Veres, Nándor Faluhelyi, Pál Miheller, Péter Hegyi, Szabolcs Ábrahám

**Affiliations:** ^1^ Centre for Translational Medicine Semmelweis University Budapest Hungary; ^2^ Department of Surgery Toldy Ferenc Hospital Cegléd Hungary; ^3^ Grigore T. Popa University of Medicine and Pharmacy Iași Romania; ^4^ Department of Internal Medicine and Hematology, Medical School Semmelweis University Budapest Hungary; ^5^ Department of Rheumatology and Immunology Semmelweis University Budapest Hungary; ^6^ Department of Biophysics and Radiation Biology Semmelweis University Budapest Hungary; ^7^ Department of Surgery, Transplantation and Gastroenterology Semmelweis University Budapest Hungary; ^8^ Institute for Translational Medicine, Medical School University of Pécs Pécs Hungary; ^9^ Institute of Pancreatic Diseases Semmelweis University Budapest Hungary; ^10^ Department of Surgery University of Szeged Szeged Hungary

**Keywords:** abdominoperineal excision, disease‐free survival, meta‐analysis, rectal neoplasms, survival analysis

## Abstract

**Background:**

In advanced low rectal cancer, the surgical technique chosen can affect long‐term oncological outcomes. Extralevator abdominoperineal excision (ELAPE) was developed to improve radicality compared with standard abdominoperineal excision (APE). Although earlier analyses suggested reduced local recurrence and mortality with ELAPE, its survival benefit remains debated, and concerns about wound complications persist. This meta‐analysis compared ELAPE and APE, focusing on oncological outcomes.

**Methods:**

The protocol was registered on PROSPERO (CRD42023412308). A systematic search of PubMed, Embase, and CENTRAL was performed (April 14, 2023). Comparative studies of ELAPE versus APE were included. Data were synthesized using random‐effects models. For overall survival (OS), disease‐free survival (DFS), and local recurrence‐free survival (LRFS), hazard ratios (HRs) with 95% confidence intervals (CIs) were calculated, with individual patient data extracted from Kaplan–Meier curves.

**Results:**

Thirty‐eight studies, including over 5000 patients, were analyzed. ELAPE reduced mortality by 46% (HR 0.54, 95% CI 0.40–0.73) and achieved a 5‐year OS of 83% (CI 0.53–0.95) versus 69% (CI 0.40–0.88) for APE. ELAPE improved DFS (HR 0.74, 95% CI 0.55–0.99), with a 5‐year DFS of 87% (CI 0.50–0.98) compared with 65% (CI 0.24–0.92) for APE. LRFS favored ELAPE but was not significant (HR 0.67, 95% CI 0.45–1.00). Rates of perineal wound complications did not differ (OR 1.39, 95% CI 0.81–2.40).

**Conclusion:**

ELAPE improves OS and DFS compared with APE without increasing wound complications. These findings support the broader application of ELAPE in rectal cancer treatment.

## Graphical abstract

This systematic review and meta‐analysis of 38 studies, encompassing over 5000 patients, compared extralevator abdominoperineal excision (ELAPE) with standard abdominoperineal excision (APE) in patients with advanced low rectal cancer. ELAPE was associated with significantly improved overall and disease‐free survival, reduced intraoperative perforation and positive circumferential resection margins, and comparable perineal wound morbidity. These findings support broader indications for ELAPE and provide robust evidence to inform surgical guidelines and clinical practice.

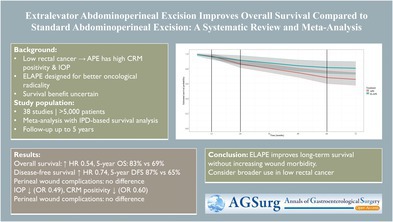

## 1 Introduction

Rectal cancer is a significant global health issue, with over 700 000 new cases diagnosed in 2020 [1]. Surgical resection is essential for long‐term survival, with a 5‐year overall survival (OS) exceeding 80% [2]. Standard abdominoperineal excision (APE) is the primary surgical option for distal rectal cancer when sphincter preservation is not feasible [3, 4]. A drawback of APE is the “waisting” of the specimen, where the tissue narrows excessively at the distal end, creating an hourglass‐like shape at the junction of abdominal and perineal dissection planes [5, 6]. This increases the risk of intraoperative perforation (IOP) and positive circumferential resection margin (CRM) [7, 8].

To address these limitations, extralevator abdominoperineal excision (ELAPE) was introduced as a modified technique to improve resection quality. ELAPE produces a wider, cylindrical specimen through extended perineal dissection, reducing IOP and improving local recurrence (LR) [9] compared to APE. The prone jackknife position further enhances surgical precision, facilitating a more complete dissection and lowering the risk of LR [10, 11].

A major dilemma in rectal cancer surgery is balancing oncological radicality with surgical morbidity. While ELAPE has been associated with reduced IOP and CRM positivity [12, 13], its impact on long‐term survival remains controversial, with some studies showing no significant improvement in OS or DFS. Additionally, ELAPE is associated with higher surgical complexity, longer operative times, and increased perineal wound morbidity, raising concerns about its overall clinical benefit [14]. Given these conflicting results and the lack of consensus on the optimal surgical approach, a comprehensive evaluation of both techniques is needed.

The aim of this study was to compare ELAPE and APE by evaluating long‐term oncological outcomes—OS, DFS, and LRFS—as well as short‐term surgical factors influencing prognosis, including CRM involvement and IOP.

## 2 Methods

The PRISMA 2020 guideline [15] and the Cochrane Handbook [16] were followed. The study protocol was preregistered on PROSPERO (CRD42023412308).

### 2.1 Protocol Deviation

One‐arm studies were excluded because of the unexpectedly high number of comparative studies.

### 2.2 Eligibility Criteria

The PICO framework was used. The **P**opulation consisted of adults with advanced low rectum cancer who underwent curative surgery, the **I**ntervention was ELAPE, while the **C**omparator was APE. The primary **O**utcomes were the long‐term oncological outcomes (OS, DFS, LRFS). Secondary **O**utcomes were intraoperative parameters (IOP, positive CRM, CRM length, blood loss, operative time), postoperative parameters (30‐day mortality, overall complications, reoperation, hospital stay), and perineal wound complications (overall complications, infection, dehiscence, perineal hernia, and perineal pain).

### 2.3 Information Sources

The systematic search was conducted on April 14, 2023, in three databases: MEDLINE (via PubMed), Embase, and Cochrane Central Register of Controlled Trials (CENTRAL). The search was done in all fields/all text in every database. No language or other filters were applied.

### 2.4 Search Strategy

The search key comprised two main domains: population and the two types of surgeries. The detailed search key can be found on the 2nd page in Supporting Information.

### 2.5 Selection Process

Two independent review authors performed the screening and selection process (S.B.K. and D.E.F.). The bibliographic management software “EndNote” was used to eliminate duplicates, with automatic and manual removal of entries with identical authors, titles, and publication years. The online platform “Rayyan” was used to evaluate studies based on their titles and abstracts, before the selection of full‐texts. Cohen's kappa coefficient (κ) was applied to evaluate inter‐rater reliability at each phase, with any disagreements resolved by a senior review author (A.R.).

On October 2nd 2024, the online automated tool “citationchaser” was used to conduct forward and backward citation searches.

### 2.6 Data Collection Process

Data were collected by two authors (S.B.K. and D.E.F.) independently using Microsoft Excel spreadsheets. Any discrepancies were resolved with the assistance of a senior review author (A.R.). In cases of publications with overlapping populations, all relevant data were retrieved from either the first publication or the publication with the most patients included.

### 2.7 Data Items

The following data were extracted: publication and study characteristics, patients' characteristics, all the outcomes presented in the eligibility criteria section, as the number of patients and events for each type of surgery, and duration of follow‐up. Lastly, preoperative and pathological TNM, oncological therapy, type of surgery (open/laparoscopic/robotic), types of perineal defect reconstruction, and tumor height were additionally collected.

Details on the handling of outcome definitions, subgroup classifications, and extraction of survival data from Kaplan–Meier curves are provided in the SM.

### 2.8 Risk of Bias and Certainty of Evidence Assessment

Based on the recommendation of the Cochrane Collaboration, the Revised Cochrane risk‐of‐bias tool ROB 2 [17] was used for RCTs, while for non‐randomized interventional studies, the Risk Of Bias In Non‐randomized Studies—of Interventions (ROBINS‐I) tool [18] was used. Small study publication bias was evaluated through visual inspection of Funnel plots and the (modified) Egger's test.

The certainty of evidence was assessed by Grading of Recommendations Assessment, Development, and Evaluation (GRADE) approach [19] and the GRADEpro software tool.

Two authors (S.B.K. and D.E.F.) independently conducted both evaluations. Any disagreements were resolved through discussion with a third reviewer (A.R.).

### 2.9 Synthesis Methods

Considerable heterogeneity among the study populations was assumed, and random effects models were used within a frequentist framework. For dichotomous outcomes, the odds ratio (OR) was calculated from the total number of patients and events in each group. For continuous outcomes, the mean difference (MD) was derived from sample size, mean, and standard deviation (SD). For time‐to‐event data, an HR with a 95% confidence interval (CI) was used, comparing ELAPE and APE groups. Two methods were applied: a classical random‐effects meta‐analysis for study HRs and an IPD‐based random‐effects Cox hazard model with Gaussian random effects. Survival probabilities were estimated at specific time points and pooled using a 3‐level model. Additionally, pooled survival curves were estimated with Pandey's method [20]. Results were summarized in tables, forest plots, and Kaplan–Meier survival curves. Heterogeneity was assessed by between‐study variance (*τ* ^2^) and Higgins' *I* ^2^ [21]. For IPD‐based outcomes, the standard error of random effects distribution was reported. Small‐study bias was evaluated using funnel plots and tests like Egger and Harbord [22]. Outlier studies were checked using leave‐one‐out influence measures as suggested by Harrer et al. [23]. *R software* [24] was used for all statistical analyses: *meta*, *dmetar*, and *metafor* packages for meta‐analysis; *survival*, *survminer* and *coxme* for IPD‐based calculations; and MetaSurvival [20] for pooled curve estimates.

Further information are presented in the Supporting Information.

## 3 Results

### 3.1 Search and Selection

**FIGURE 1:**
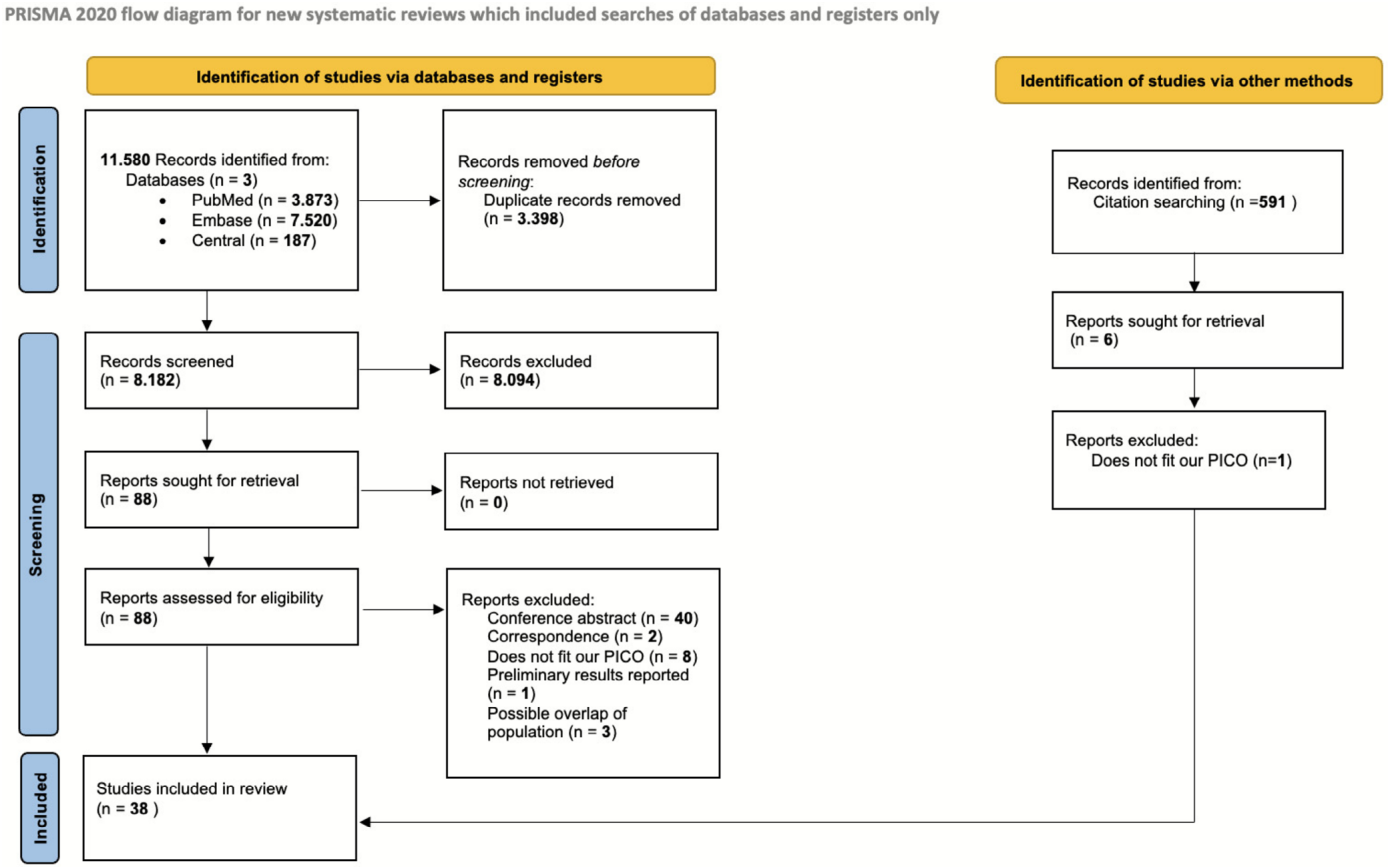
PRISMA 2020 flowchart representing the study selection process. *From*: Page et al. [15]. For more information, visit: http://www.prisma‐statement.org/.

Out of 12 171 studies screened, 38 were included in the final analysis. The reasons for excluding studies can be found in Table S1. The selection process is illustrated in Figure 1.

### 3.2 Basic Characteristics of Included Studies

Baseline characteristics are shown in Table S2. Four studies were RCTs, one prospective case–control study, one propensity case‐matched analysis, and one prospective study, the remainder were retrospective cohorts.

Neoadjuvant therapy was generally balanced, though some reported higher use in ELAPE. Tumor height was generally comparable, with a few studies favoring ELAPE in lower tumors.

In four studies, both groups underwent purely laparoscopic procedures. Fourteen articles showed a balanced distribution of open and laparoscopic techniques. Seven studies reported more frequent use of laparoscopic or robotic approaches in the ELAPE group, while Bianco et al. utilized these methods more in the APE group. West et al. had significant missing data, and 11 studies did not report it.

TNM classification was highly inconsistent across studies, with mixed use of clinical, pathological, and posttreatment staging, so a reliable summary was possible only for the seven OS studies (Table S3). Overall, tumor staging appeared broadly comparable. Since most studies did not provide stratified outcome data based on pathological stage or tumor location, meaningful pooled analyses by these factors were not possible.

### 3.3 Long‐Term Oncological Outcomes

#### 3.3.1 Overall Survival

Although OS was reported in 10 studies, only seven provided extractable data because of poor Kaplan–Meier curve quality; qualitative findings from the excluded studies are summarized at the end of this subsection.

**FIGURE 2:**
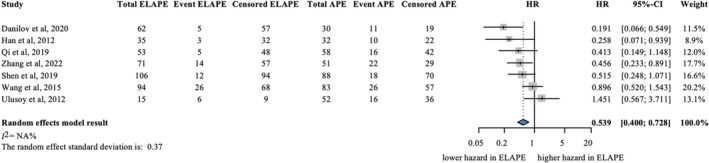
Forest plots representing overall survival following ELAPE vs. APE—IPD‐based analysis.

Patients undergoing ELAPE had approximately a 47% lower risk of death than those receiving APE over the entire follow‐up period (HR: 0.54, CI: 0.40–0.73 from IPD—see Figure 2; for sensitivity analysis, see Figure S1).

**FIGURE 3:**
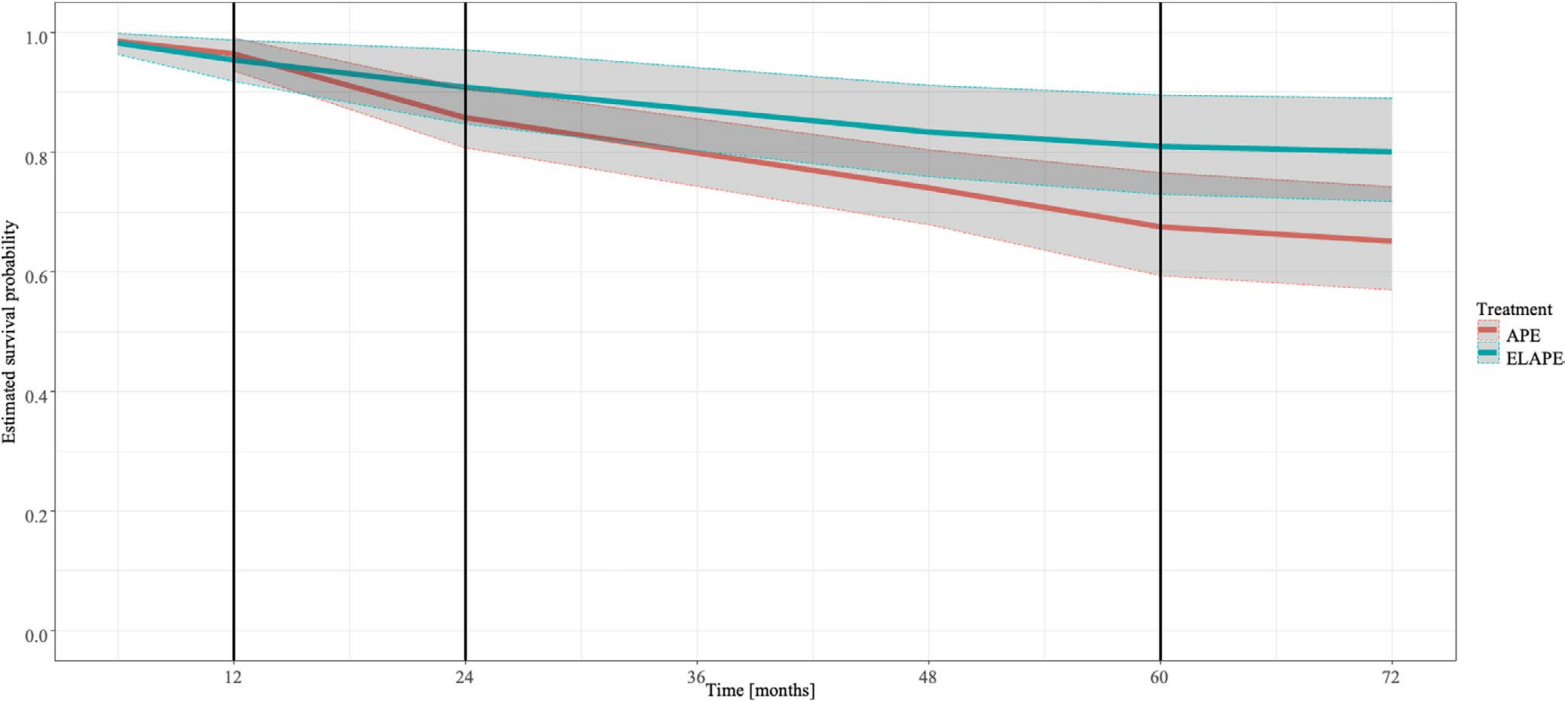
Estimated survival probabilities at given times following ELAPE vs. APE.

At 12 months, the two groups' estimated survival probabilities were similar: 98% (CI: 0.92–0.99) for APE and 97% (CI: 0.90–0.99) for ELAPE. However, by 24 months, a divergence emerged, with survival declining slightly in both groups but remaining higher in the ELAPE cohort (93% for ELAPE (CI: 0.80–0.97) vs. 86% for APE (CI: 0.68–0.95)). The clinically relevant difference was observed at 5 years, where the ELAPE group showed a relevantly better survival rate of 83% (CI: 0.53–0.95) compared to 69% (CI: 0.40–0.88) for APE. See Figure 3 and Figures S2–S6.

Carpelan et al. [25] reported mean follow‐up times of 3.2 years for ELAPE and 5.8 years for APE, with no significant OS difference (*p* = 0.8173). Klein et al. [26] found 4‐year OS rates of 74% for APE and 77% for ELAPE (*p* = 0.59). Similarly, Prytz et al. [27] observed no difference in 3‐year OS between the two groups. For transparency, we also generated exploratory forest plots based on reconstructed survival estimates from the two registry studies; these are available in the SM (Figure S7).

#### 3.3.2 Disease‐Free Survival

**FIGURE 4:**
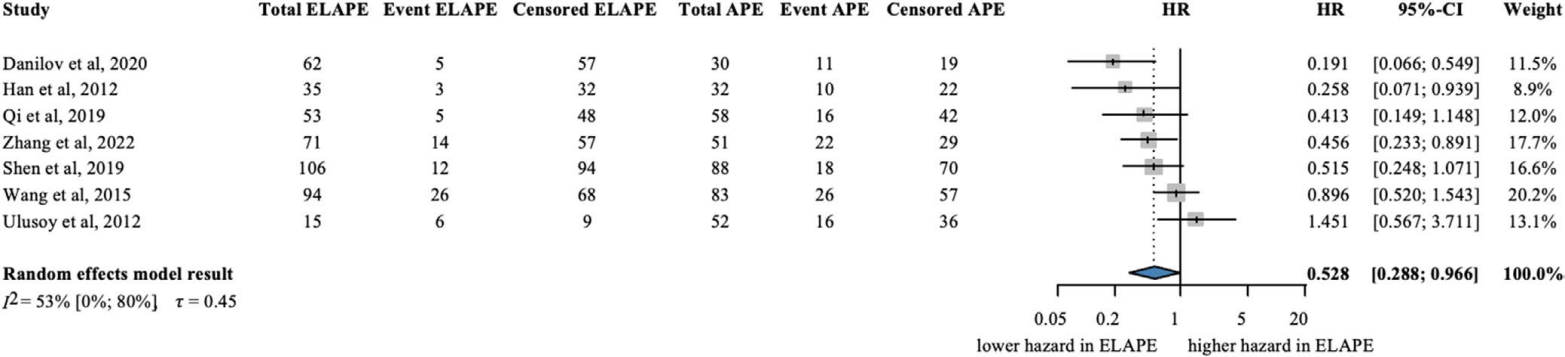
Forest plots representing disease free survival following ELAPE vs. APE using IPD‐based analysis.

Data from five articles showed a 26% lower risk of DFS in the ELAPE group over the entire follow‐up period (HR: 0.74, CI: 0.55–0.99 from IPD—see Figure 4; for sensitivity analysis, see Figure S8).

**FIGURE 5:**
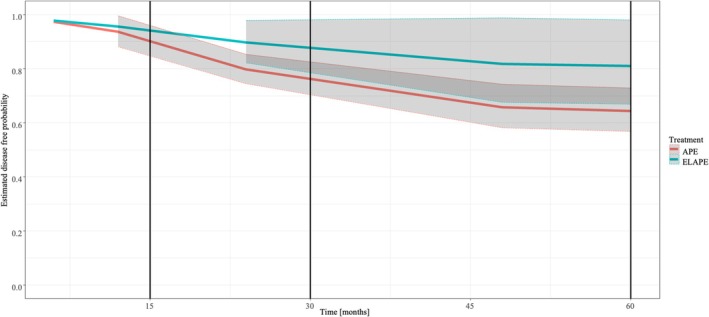
Estimated DFS probabilities at given times following ELAPE vs. APE.

At 12 months, the estimated DFS probability was slightly higher for ELAPE (98%, CI: 0.87–1.00) compared to APE (96%, CI: 0.79–0.99). By 24 months, the gap widened, with DFS at 78% (CI: 0.50–0.93) for APE and 89% (CI: 0.68–0.97) for ELAPE. At 5 years, ELAPE demonstrated a notably higher DFS of 87% (CI: 0.50–0.98), while APE had a DFS of 65% (CI: 0.24–0.92)—see Figure 5 and Figures S9–S13.

Carpelan et al. [25] observed mean follow‐up times of 3.2 years for ELAPE and 5.8 years for APE, with no significant difference in DFS (*p* = 0.6311). Klein et al. [26] found 4‐year DFS rates of 67% for APE and 66% for ELAPE (*p* = 0.82). An exploratory forest plot based on reconstructed survival estimates from the registry study is available in the SM (Figure S14).

#### 3.3.3 Local Recurrence‐Free Survival

**FIGURE 6:**
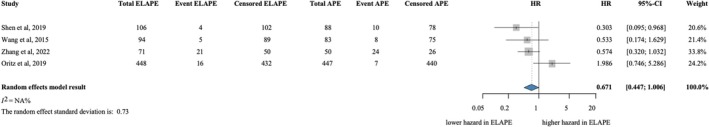
Forest plots representing local recurrence free survival following ELAPE vs. APE using IPD‐based analysis.

Data from four studies suggested a 33% lower risk of LR in the ELAPE group over the entire follow‐up period, but the results did not reach statistical significance (HR: 0.67, CI: 0.45–1.00 from IPD—see Figure 6; HR: 0.67, CI: 0.2–2.25 from aggregated data—see Figure S15).

**FIGURE 7:**
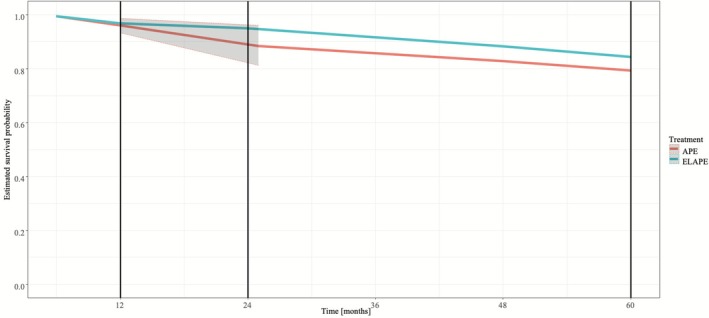
Estimated LRFS probabilities at given times following ELAPE vs. APE.

At 12 months, the estimated LRFS probability was slightly higher for ELAPE (98%, CI: 0.89–1.00) compared to APE (96%, CI: 0.78–0.99). By 24 months, the advantage of ELAPE became more pronounced (96%, CI: 0.68–1.00 for ELAPE vs. 87%, CI: 0.46–0.98 for APE). At 5 years, ELAPE maintained a higher LRFS (81%, CI: 0.02–1.00) compared to APE (72%, CI: 0.02–1.00), though the wide confidence intervals indicate uncertainty in these estimates. For more details, see Figure 7 and Figures S16–S19.

Carpelan et al. [25] reported local recurrence rates of 7% for ELAPE and 19% for APE (*p* = 0.2473), suggesting a lower recurrence risk with ELAPE. In contrast, Prytz et al. [27] found higher 3‐year local recurrence rates for ELAPE compared to APE, with a relative risk of 4.91 (median follow‐up: 3.43 years).

### 3.4 Intraoperative Parameters

ELAPE significantly reduced IOP (OR: 0.49; CI: 0.34–0.72), with an even greater effect in RCTs (OR: 0.22; CI: 0.07–0.73), corresponding to 78% lower odds of perforation compared to APE.

ELAPE was associated with lower CRM positivity (OR: 0.60; CI: 0.40–0.90), with an even greater reduction in the RCT subgroup (OR: 0.15; CI: 0.06–0.42), corresponding to 85% lower odds compared with APE.

ELAPE resulted in less blood loss (MD: −54.68 mL; CI: −95.42 to −13.93 mL), but a longer operative time by about 1 h (MD 61.72 min; CI: 38.7–84.76 min), compared to APE.

See Table 1 in the manuscript and Figures S20–S25 in the SM.

**TABLE 1:** Results of the secondary outcomes.

Outcome of interest	Nr. of studies included	Nr. of patients (ELAPE/APE)	MD/OR (95% CI)	Study heterogeneity	
				*I* ^2^ (95% CI)	*τ*
Intraoperative parameters					
IOP	27	2241/1932	0.49 (0.34; 0.72)	39% (3%; 62%)	0.25
IOP—subgroup of RCT's only	4	82/79	0.22 (0.07; 0.73)	0% (0%; 85%)	0
Positive CRM	30	2423/2116	0.60 (0.40; 0.90)	64% (47%; 76%)	0.5
Positive CRM—subgroup of RCT's only	4	82/79	0.16 (0.06; 0.42)	0% (0%; 85%)	0
Blood loss	14	1365/946	−54.68 (−95.42; −13.93)	77% (62%; 86%)	1748.03
Operative time	15	1179/816	61.73 (38.70; 84.76)	88% (81%; 92%)	1287.56
Postoperative parameters					
Mortality	18	1567/1325	0.90 (0.64; 1.26)	0% (0%; 50%)	0
Mortality—subgroup of RCT's only	3	62/59	0.45 (0.03; 5.76)	0% (0%; 90%)	0
Overall complications	8	1209/937	1.06 (0.69; 1.63)	40% (0%; 73%)	0.11
Reoperation	8	1171/997	1.18 (0.84; 1.66)	0% (0%; 68%)	0
Hospital stay	11	791/694	0.11 (−2.53; 2.75)	94% (91%; 96%)	13.14
Perineal wound complications					
Overall complications	13	1173/1244	1.39 (0.81; 2.40)	72% (50%; 84%)	0.51
Infection	19	1045/1052	1.02 (0.69; 1.50)	40% (0%; 65%)	0.24
Infection—subgroup of RCT's only	3	72/69	0.82 (0.13; 5.06)	0% (0%; 90%)	0
Dehiscence	9	603/712	1.72 (0.96; 3.09)	29% (0%; 67%)	0.14
Perineal hernia	13	789/803	0.84 (0.37; 1.87)	33% (0%; 65%)	0.43
Perineal pain	5	386/388	3.59 (1.10; 11.74)	44% (0%; 79%)	0.41

Abbreviations: APE, abdominoperineal excision; CI, confidence interval; CRM, circumferential resection margin; ELAPE, extralevator abdominoperineal excision; IOP, intraoperative perforation; MD, mean difference; OR, odds ratio; RCT, randomized controlled trial.

### 3.5 Postoperative Parameters

Analysis of mortality, overall complications, reoperations, and hospital stay revealed no significant differences between ELAPE and APE. For further details: Table 1—manuscript and Figures S26–S30.

### 3.6 Perineal Wound Complications

Analysis of overall perineal wound complications and infections revealed no significant differences. While the OR of 1.72 may suggests a higher rate of perineal wound dehiscence with ELAPE, the confidence interval (CI: 0.96–3.09), indicating no statistically significant difference. Perineal hernia occurrence was similar (OR: 0.84; CI: 0.37–1.87). However, perineal pain was more frequently reported in ELAPE patients (OR: 3.59; CI: 1.1–11.74).

See Table 1 in the manuscript and Figures S31–S36.

### 3.7 Risk of Bias Assessment

The RoB assessment for non‐randomized studies is presented in Table S4. Most included studies demonstrated an overall moderate RoB. Three studies had a serious RoB, while one study exhibited a low RoB. From the four RCTs included, three had an overall low RoB, while one showed some concerns in the randomization process. For more information, see Table S5.

### 3.8 Publication Bias and Heterogeneity

Visual analysis of funnel plots (see Figures S37–S56) showed no potential small study bias for any evaluated outcomes. While heterogeneity was low in the RCT subgroup analyses, considerable between‐study heterogeneity was observed in the pooled analyses of observational studies.

### 3.9 Certainty of Evidence

The certainty of evidence was low to very low for most outcomes with concerns regarding risk of bias, inconsistency, and imprecision. However, intraoperative outcomes showed moderate certainty in RCT subgroups. See Table S6.

## 4 Discussion

This study provides a comprehensive analysis comparing ELAPE and APE in advanced low rectal cancer, focusing on oncological and perioperative parameters. ELAPE was associated with improved OS and DFS, as well as significant advantages in IOP and CRM positivity. Perineal wound complications did not differ significantly, suggesting that concerns about increased morbidity with ELAPE may be overstated.

Patients undergoing ELAPE had a 7 percentage point higher OS at 24 months and a 14 percentage point advantage at 60 months, meaning that for every 100 patients, 14 more survived at 5 years with ELAPE. DFS followed a similar pattern, with a 9‐point higher probability at 24 months and a 22‐point difference at 60 months, indicating fewer recurrences over time. The IPD‐based analysis confirmed these survival benefits as statistically significant, reinforcing the oncological advantage of extended resection. While LRFS showed a 9‐point improvement at 60 months, this difference did not reach statistical significance. Although this trend favors ELAPE, the available evidence is insufficient to confirm a true reduction in local recurrence. Despite the conceptual advantage of improved pelvic control through reduced CRM involvement and IOP, the lack of significance likely reflects the limited amount of extractable data, the small number of events, and the wide confidence intervals that substantially reduce statistical power.

The discrepancy between improved OS/DFS and unchanged LRFS suggests that factors beyond pelvic control may have contributed to the survival difference. Reporting on distant metastasis, adjuvant chemotherapy, and major complications was inconsistent, and some studies used neoadjuvant therapy more frequently in the ELAPE group, potentially enhancing systemic control. It is also possible that centre‐related differences or subtle selection effects favored ELAPE.

Surgeon selection bias is an additional consideration. In non‐randomized cohorts, the choice between ELAPE and APE is shaped by surgeon judgment, tumor anatomy, and centre‐specific expertise. ELAPE is often selected for distal, bulky, or technically challenging tumors, while APE may be preferred for less complex cases. Although reported baseline characteristics appeared broadly comparable, these measures do not capture key operative nuances. Unmeasured factors, including tumor fixation, response to neoadjuvant therapy, pelvic dimensions, or concerns about a threatened CRM, may have influenced procedure selection and could partly account for the observed differences between techniques.

The studies excluded [25, 26, 27] due to poor Kaplan–Meier curve quality nonetheless provide important context for interpreting our findings. Two large‐scale, population‐based analyses [26, 27] reported no significant differences in OS or DFS after adjustment for key prognostic factors, while Prytz et al. [27] also observed higher LR rates in the ELAPE group, particularly among patients with smaller tumors. These patterns likely reflect limitations of registry data, including residual confounding, imprecise or incomplete coding of tumor characteristics, neoadjuvant therapy, CRM status, and surgical technique, as well as high rates of missing or unverifiable data. ELAPE was also more frequently used for very distal or advanced tumors, introducing selection effects that may have biased outcomes. In addition, heterogeneity in the perineal dissection technique could not be assessed, further complicating interpretation.

To assess the potential influence of these datasets, we generated exploratory forest plots based on reconstructed OS and DFS estimates from the two registry studies. Incorporating these data shifted pooled effects toward the null, with OS remaining significant but attenuated and DFS crossing the line of no effect. However, the reconstructed estimates were derived from Kaplan–Meier curves lacking censoring information and presented with insufficient graphical resolution, yielding unstable and potentially biased survival estimates. As all other included studies provided extractable, censoring‐appropriate data, integrating these would have reduced analytical consistency. For this reason, the exploratory plots are provided solely for transparency, and the quantitative synthesis relies exclusively on studies meeting our predefined criteria for accurate and reproducible survival extraction.

Perineal wound complications did not differ significantly, countering concerns that the larger perineal defect and use of reconstruction techniques in ELAPE might increase these risks [28]. In the APE group, perineal closure was primarily performed with direct suturing, while ELAPE required various reconstructive approaches, including mesh placement (both synthetic and biological), Vertical Rectus Abdominis Myocutaneous flap, gluteus maximus myocutaneous flap, V‐Y advancement flap, and gracilis flap. Despite these differences, complication rates remained comparable, likely because the use of well‐vascularized flaps or biological mesh in ELAPE may counteract the effect of the larger perineal defect by improving tissue coverage and reducing closure tension. Additionally, enhancements in perioperative care in more recent ELAPE cohorts, including standardized antibiotic prophylaxis and more structured postoperative wound management, may also contribute to comparable outcomes.

Perineal pain was significantly more frequent in ELAPE (OR 3.59), representing an important postoperative morbidity that should be weighed against the potential oncological advantages. This increased pain burden is likely related to the more extensive perineal dissection and the more frequent use of coccygectomy in ELAPE.

Sexual and urinary dysfunction are important long‐term considerations after rectal cancer surgery. Although ELAPE involves a wider perineal dissection, both techniques share the same abdominal phase—where most autonomic nerves are encountered—and the perineal dissection in ELAPE occurs below the hypogastric plexus, making major differences in autonomic injury unlikely. The included studies assessed these outcomes using heterogeneous and noncomparable questionnaires, often with small samples or substantial missing data, and several reported only early postoperative urinary retention rather than long‐term dysfunction. Owing to this variability, quantitative synthesis was not possible; however, the available evidence does not indicate meaningful differences in long‐term sexual or urinary dysfunction.

ELAPE surgery is approximately 1 h longer than APE. This difference is largely due to the prone jackknife position used during the perineal dissection in ELAPE. After completing the abdominal phase, the intubated patient must be repositioned, which is a time‐consuming process. Many surgeons are more familiar with the lithotomy position, while the prone approach changes the anatomical view and requires more cautious dissection. However, with increasing experience and familiarity, we anticipate that operative times for ELAPE will decrease in the future.

The lower intraoperative blood loss associated with ELAPE likely reflects the greater use of laparoscopic or robotic approaches, which reduce venous oozing through improved visualization and the tamponade effect of pneumoperitoneum. The standardized cylindrical dissection of ELAPE, which remains outside the levator musculature and sphincter complex, may also limit bleeding from the pelvic floor. Lower conversion rates and the greater involvement of specialist colorectal surgeons may further contribute to this finding.

While prior analyses highlighted a significant reduction in IOP with ELAPE, they did not find significant differences in positive CRM rates [9, 29]. In contrast, our study demonstrates significant benefits in both, reinforcing the advantages of a more extensive resection. Moreover, we have further validated these findings through a subgroup analysis of RCT studies, confirming the consistency and robustness of our results.

Prior studies have reported inconsistent oncological outcomes, often focusing on short‐term measures. Negoi et al. [29] found no difference in 1‐year OS but observed improved 3‐year OS with ELAPE. Qi et al. [9] reported a significantly lower LR rate for ELAPE contradicting Negoi et al., who found no such benefit. These discrepancies stem from methodological differences, particularly the reliance on ORs, which fail to account for event timing. Our meta‐analysis overcomes these limitations by using HRs for a time‐dependent evaluation of all three key oncological outcomes over a longer, 5‐year follow‐up period. Additionally, incorporating IPD minimizes biases and enhances statistical power. By refining the assessment of ELAPE's long‐term oncological impact, our study provides a clearer evidence base for surgical decision‐making.

### 4.1 Strengths and Limitations

A meticulous methodology was adhered to, including risk‐of‐bias and GRADE assessments. A strength of this study is its use of HRs, IPD, and subgroup analyses of RCTs for multiple outcomes.

The main limitations are the predominance of non‐randomized studies, the lack of long‐term follow‐up studies, and the lack of possibility for adjusting confounding factors. Additionally, the poor quality of some Kaplan–Meier curves resulted in the exclusion of data from two major studies with large sample sizes.

### 4.2 Implications for Practice and Research

The effective translation of these findings into clinical decision‐making is essential for optimizing patient outcomes [30, 31]. Current guidelines [3, 32] recommend ELAPE mainly for bulky, locally advanced tumors with levator involvement; however, our results challenge this restrictive approach. The consistent oncological advantages observed suggest that ELAPE may provide benefits across tumor stages, supporting broader indications. Surgeons should balance these benefits with transparency about potential postoperative issues such as perineal pain.

Future research should include RCTs with long‐term follow‐up, use multivariate survival models for more nuanced insights, and explore ELAPE across different TNM stages to refine patient selection. To support reproducibility, anonymized datasets should be shared for external validation.

## 5 Conclusion

ELAPE offers clinically relevant oncological advantages over APE, with improved OS, DFS, LRFS, and reduced rates of IOP and CRM positivity. Despite the extended resection, perineal wound complications were similar between the two techniques.

## Author Contributions

**Sarolta Beáta Kávási:** conceptualization, investigation, project administration, validation, data curation, writing – original draft. **Diana‐Elena Floria:** conceptualization, investigation, data curation, writing – review and editing. **Anett Rancz:** conceptualization, methodology, project administration, validation, writing – review and editing. **Dániel Sándor Veres:** conceptualization, writing – review and editing, formal analysis, software. **Nándor Faluhelyi:** conceptualization, writing – review and editing. **Pál Miheller:** conceptualization, writing – review and editing. **Péter Hegyi:** conceptualization, writing – review and editing. **Szabolcs Ábrahám:** conceptualization, supervision, validation, writing – original draft.

## Funding

The authors have nothing to report.

## Ethics Statement

The authors have nothing to report.

## Conflicts of Interest

The authors declare no conflicts of interest.

## Supporting information


**Table S1:** Reasons for exclusion during full‐text assessment.
**Table S2:** Baseline characteristics of the enrolled studies.
**Table S3:** TNM Staging in Studies Included in the OS Analysis.
**Table S4:** Risk of bias assessment for the non‐randomized studies by ROBINS tool.
**Table S5:** Risk of bias assessment for the randomized studies by ROB‐2 tool.
**Table S6:** Detailed assessment of the certainty of the evidence for the comparison of ELAPE to APE.
**Figure S1:** Forest plots representing overall survival following ELAPE vs. APE using “classical” analysis.
**Figure S2:** Estimated survival probabilities at given times following ELAPE vs. APE.
**Figure S3:** Estimated survival probability at 12 months following extralevator abdominoperineal excision vs. standard abdominoperineal excision.
**Figure S4:** Estimated survival probability at 24 months following extralevator abdominoperineal excision vs. standard abdominoperineal excision.
**Figure S5:** Estimated survival probability at 60 months following extralevator abdominoperineal excision vs. standard abdominoperineal excision.
**Figure S6:** Kaplan–Meier curves for overall survival, showing individual study‐specific survival probabilities for extralevator abdominoperineal excision and standard abdominoperineal excision.
**Figure S7:** Forest plot representing exploratory pooled overall survival effects for ELAPE vs. APE after integrating the two registry studies with the previously analyzed data.
**Figure S8:** Forest plots representing disease‐free survival following ELAPE vs. APE using “classical” analysis.
**Figure S9:** Estimated DFS probabilities at given times following ELAPE vs. APE.
**Figure S10:** Estimated disease‐free survival probability at 12 months following extralevator abdominoperineal excision vs. standard abdominoperineal excision.
**Figure S11:** Estimated disease‐free survival probability at 24 months following extralevator abdominoperineal excision vs. standard abdominoperineal excision.
**Figure S12:** Estimated disease‐free survival probability at 60 months following extralevator abdominoperineal excision vs. standard abdominoperineal excision.
**Figure S13:** Kaplan–Meier curves for disease‐free survival, showing individual study‐specific survival probabilities for extralevator abdominoperineal excision and standard abdominoperineal excision.
**Figure S14:** Forest plot representing exploratory pooled disease‐free survival effects for ELAPE vs. APE after integrating the two registry studies with the previously analyzed data.
**Figure S15:** Forest plots representing local recurrence free survival following ELAPE vs. APE using “classical” analysis.
**Figure S16:** Estimated recurrence‐free survival probability at 12 months following extralevator abdominoperineal excision vs. standard abdominoperineal excision.
**Figure S17:** Estimated recurrence‐free survival probability at 12 months following extralevator abdominoperineal excision vs. standard abdominoperineal excision.
**Figure S18:** Estimated recurrence‐free survival probability at 60 months following extralevator abdominoperineal excision vs. standard abdominoperineal excision.
**Figure S19:** Kaplan–Meier curves for local‐recurrence‐free survival, showing individual study‐specific survival probabilities for extralevator abdominoperineal excision and standard abdominoperineal excision.
**Figure S20:** Forest plot comparing the risk of intraoperative perforation following extralevator abdominoperineal excision to standard abdominoperineal excision.
**Figure S21:** Forest plot comparing the risk of intraoperative perforation following extralevator abdominoperineal excision to standard abdominoperineal excision—subgroup of RCT's.
**Figure S22:** Forest plot comparing the risk of positive CRM following extralevator abdominoperineal excision to standard abdominoperineal excision.
**Figure S23:** Forest plot comparing the risk of positive CRM following extralevator abdominoperineal excision to standard abdominoperineal excision—subgroup of RCT's.
**Figure S24:** Forest plot comparing the blood loss during extralevator abdominoperineal excision to standard abdominoperineal excision.
**Figure S25:** Forest plot comparing the operative time of extralevator abdominoperineal excision to standard abdominoperineal excision.
**Figure S26:** Forest plot comparing the mortality following extralevator abdominoperineal excision to standard abdominoperineal excision.
**Figure S27:** Forest plot comparing the mortality following extralevator abdominoperineal excision to standard abdominoperineal excision—subgroup of RCT's.
**Figure S28:** Forest plot comparing the overall complications following extralevator abdominoperineal excision to standard abdominoperineal excision.
**Figure S29:** Forest plot comparing the reoperation rates following extralevator abdominoperineal excision to standard abdominoperineal excision.
**Figure S30:** Forest plot comparing the hospital stay following extralevator abdominoperineal excision to standard abdominoperineal excision.
**Figure S31:** Forest plot comparing the perineal wound complication rates following extralevator abdominoperineal excision to standard abdominoperineal excision.
**Figure S32:** Forest plot comparing the perineal wound infection rates following extralevator abdominoperineal excision to standard abdominoperineal excision.
**Figure S33:** Forest plot comparing the perineal wound infection rates following extralevator abdominoperineal excision to standard abdominoperineal excision—subgroup of RCT's.
**Figure S34:** Forest plot comparing the perineal wound dehiscence rates following extralevator abdominoperineal excision to standard abdominoperineal excision.
**Figure S35:** Forest plot comparing the perineal hernia rates following extralevator abdominoperineal excision to standard abdominoperineal excision.
**Figure S36:** Forest plot comparing the existence of perineal pain following extralevator abdominoperineal excision to standard abdominoperineal excision.
**Figure S37:** Funnel plot for overall survival following extralevator abdominoperineal excision compared to standard abdominoperineal excision.
**Figure S38:** Funnel plot for disease‐free survival following extralevator abdominoperineal excision compared to standard abdominoperineal excision.
**Figure S39:** Funnel plot for local‐recurrence‐free survival following extralevator abdominoperineal excision compared to standard abdominoperineal excision.
**Figure S40:** Funnel plot for IOP rates following extralevator abdominoperineal excision compared to standard abdominoperineal excision.
**Figure S41:** Funnel plot for IOP rates following extralevator abdominoperineal excision compared to standard abdominoperineal excision—subgroup of RCT's.
**Figure S42:** Funnel plot for positive CRM rates following extralevator abdominoperineal excision compared to standard abdominoperineal excision.
**Figure S43:** Funnel plot for positive CRM rates following extralevator abdominoperineal excision compared to standard abdominoperineal excision—subgroup of RCT's.
**Figure S44:** Funnel plot for the difference in blood loss during extralevator abdominoperineal excision compared to standard abdominoperineal excision.
**Figure S45:** Funnel plot for the difference in operative time during extralevator abdominoperineal excision compared to standard abdominoperineal excision.
**Figure S46:** Funnel plot for mortality following extralevator abdominoperineal excision compared to standard abdominoperineal excision.
**Figure S47:** Funnel plot for mortality following extralevator abdominoperineal excision compared to standard abdominoperineal excision—subgroup of RCT's.
**Figure S48:** Funnel plot for overall complications following extralevator abdominoperineal excision compared to standard abdominoperineal excision.
**Figure S49:** Funnel plot for reoperation rates following extralevator abdominoperineal excision compared to standard abdominoperineal excision.
**Figure S50:** Funnel plot for hospital stay following extralevator abdominoperineal excision compared to standard abdominoperineal excision.
**Figure S51:** Funnel plot for perineal wound complications following extralevator abdominoperineal excision compared to standard abdominoperineal excision.
**Figure S52:** Funnel plot for perineal wound infections following extralevator abdominoperineal excision compared to standard abdominoperineal excision.
**Figure S53:** Funnel plot for perineal wound infections following extralevator abdominoperineal excision compared to standard abdominoperineal excision—subgroup for RCT's.
**Figure S54:** Funnel plot for perineal wound dehiscence following extralevator abdominoperineal excision compared to standard abdominoperineal excision.
**Figure S55:** Funnel plot for perineal hernia following extralevator abdominoperineal excision compared to standard abdominoperineal excision.
**Figure S56:** Funnel plot for the existence of perineal wound pain following extralevator abdominoperineal excision compared to standard abdominoperineal excision.
