# Supplementary material for: Extralevator Abdominoperineal Excision Improves Overall Survival Compared to Standard Abdominoperineal Excision: A Systematic Review and Meta‐Analysis
Source: Ann Gastroenterol Surg. 2026 Jan 25;10(4):1066–76. doi: 10.1002/ags3.70182 (PMC13327026; doi:10.1002/ags3.70182)
Supplement: Supplementary file 1 — Table S1: Reasons for exclusion during full‐text assessment. Table S2: Baseline characteristics of the enrolled studies. Table S3: TNM Staging in Studies Included in the OS Analysis. Table S4: Risk of bias assessment for the non‐randomized studies by ROBINS tool. Table S5: Risk of bias assessment for the randomized studies by ROB‐2 tool. Table S6: Detailed assessment of the certainty of the evidence for the comparison of ELAPE to APE. Figure S1: Forest plots representing overall survival following ELAPE vs. APE using “classical” analysis. Figure S2: Estimated survival probabilities at given times following ELAPE vs. APE. Figure S3: Estimated survival probability at 12 months following extralevator abdominoperineal excision vs. standard abdominoperineal excision. Figure S4: Estimated survival probability at 24 months following extralevator abdominoperineal excision vs. standard abdominoperineal excision. Figure S5: Estimated survival probability at 60 months following extralevator abdominoperineal excision vs. standard abdominoperineal excision. Figure S6: Kaplan–Meier curves for overall survival, showing individual study‐specific survival probabilities for extralevator abdominoperineal excision and standard abdominoperineal excision. Figure S7: Forest plot representing exploratory pooled overall survival effects for ELAPE vs. APE after integrating the two registry studies with the previously analyzed data. Figure S8: Forest plots representing disease‐free survival following ELAPE vs. APE using “classical” analysis. Figure S9: Estimated DFS probabilities at given times following ELAPE vs. APE. Figure S10: Estimated disease‐free survival probability at 12 months following extralevator abdominoperineal excision vs. standard abdominoperineal excision. Figure S11: Estimated disease‐free survival probability at 24 months following extralevator abdominoperineal excision vs. standard abdominoperineal excision. Figure S12: Estimated disease‐free survival probability at 60 mo [file AGS3-10-1066-s001.docx]

**Title**

**Extralevator Abdominoperineal Excision Improves Overall Survival Compared to Standard Abdominoperineal Excision: A Systematic Review and Meta-Analysis**

**Authors**

Sarolta Beáta Kávási ^1,2^, Diana - Elena Floria ^1,3^, Anett Rancz ^1,4^, Dániel Sándor Veres ^1,5^, Nándor Faluhelyi ^1^, Pál Miheller ^1,6^, Péter Hegyi ^1,7,9^, Szabolcs Ábrahám ^1,9^

**Affiliations**

1. Centre for Translational Medicine, Semmelweis University, Budapest, Hungary

2. Department of Surgery, Toldy Ferenc Hospital, Cegléd, Hungary

3. Grigore T. Popa University of Medicine and Pharmacy, Iași, Romania

4. Department of Internal Medicine and Hematology, Semmelweis University, Medical School, Budapest, Hungary

5. Department of Biophysics and Radiation Biology, Semmelweis University, Budapest, Hungary

6. Department of Surgery, Transplantation and Gastroenterology, Semmelweis University, Budapest

7. Institute for Translational Medicine, Medical School, University of Pécs, Pécs, Hungary

8. Institute of Pancreatic Diseases, Semmelweis University, Budapest, Hungary

9. Department of Surgery, University of Szeged, Hungary

**E-mail addresses**

Sarolta Beáta Kávási: [kavasisarolta@yahoo.com](mailto:kavasisarolta@yahoo.com)

Diana - Elena Floria: [iovdiana95@gmail.com](mailto:iovdiana95@gmail.com)

Anett Rancz: [rancz.anett@gmail.com](mailto:rancz.anett@gmail.com)

Dániel Sándor Veres: [veres.daniel@med.semmelweis-univ.hu](mailto:veres.daniel@med.semmelweis-univ.hu)

Nándor Faluhelyi: [nandor.faluhelyi@gmail.com](mailto:nandor.faluhelyi@gmail.com)

Pál Miheller: [pmiheller@gmail.com](mailto:pmiheller@gmail.com)

Péter Hegyi: [hegyi2009@gmail.com](mailto:hegyi2009@gmail.com)

Szabolcs Ábrahám: [abraham.szabolcs@med.u-szeged.hu](mailto:abraham.szabolcs@med.u-szeged.hu)

**Corresponding author**

Szabolcs Ábrahám M.D., Ph.D.

Postal address: Semmelweis u. 8., H-6725 Szeged, Hungary

Tel: +36/62/545462

E-mail address: [abraham.szabolcs@med.u-szeged.hu](mailto:abraham.szabolcs@med.u-szeged.hu)

**Supplementary Materials - Index**

| **Prisma checklist**  **Supplementary Methods** | *pag. 3-6* |
| --- | --- |
| Searchkey | *pag. 7* |
| Data items | *pag. 7* |
| Detailed synthesis method | *pag. 7-8* |
| **Supplementary Results** | *pag. 9* |
| **Supplementary Figures and Tables** |  |
| Reasons for exclusion during full-text assessment | *pag. 9-15* |
| Baseline characteristics of the enrolled studies | *pag. 15-19* |
| TNM Staging in Studies Included in the OS Analysis | *pag. 19-21* |
| OS: Figures S1 – S6 | *pag. 21-24* |
| DFS: Figures S7 – S12 | *pag. 24-26* |
| LRFS: Figures S12 – S18  Secondary outcomes: Figures S19 – S35  ROB  Funnel plots: Figures S36 – S55  Certainty of the evidence | *pag. 27-29*  *pag. 30-38*  *pag. 38-40*  *pag. 41-50*  *pag. 51-56* |
| **References** | *pag. 57-58* |
|  |  |

**Prisma checklist**

| **Section and Topic** | **Item #** | **Checklist item** | **Location where item is reported** |
| --- | --- | --- | --- |
| **TITLE** | | |  |
| Title | 1 | Identify the report as a systematic review. | Page 1 |
| **ABSTRACT** | | |  |
| Abstract | 2 | See the PRISMA 2020 for Abstracts checklist. | Page 3 |
| **INTRODUCTION** | | |  |
| Rationale | 3 | Describe the rationale for the review in the context of existing knowledge. | Page 5 |
| Objectives | 4 | Provide an explicit statement of the objective(s) or question(s) the review addresses. | Page 5 |
| **METHODS** | | |  |
| Eligibility criteria | 5 | Specify the inclusion and exclusion criteria for the review and how studies were grouped for the syntheses. | Page 6 |
| Information sources | 6 | Specify all databases, registers, websites, organisations, reference lists and other sources searched or consulted to identify studies. Specify the date when each source was last searched or consulted. | Page 6 |
| Search strategy | 7 | Present the full search strategies for all databases, registers and websites, including any filters and limits used. | Page 6 |
| Selection process | 8 | Specify the methods used to decide whether a study met the inclusion criteria of the review, including how many reviewers screened each record and each report retrieved, whether they worked independently, and if applicable, details of automation tools used in the process. | Page 6 |
| Data collection process | 9 | Specify the methods used to collect data from reports, including how many reviewers collected data from each report, whether they worked independently, any processes for obtaining or confirming data from study investigators, and if applicable, details of automation tools used in the process. | Page 6 |
| Data items | 10a | List and define all outcomes for which data were sought. Specify whether all results that were compatible with each outcome domain in each study were sought (e.g. for all measures, time points, analyses), and if not, the methods used to decide which results to collect. | Page 6-7 |
|  | 10b | List and define all other variables for which data were sought (e.g. participant and intervention characteristics, funding sources). Describe any assumptions made about any missing or unclear information. | Page 6-7 |
| Study risk of bias assessment | 11 | Specify the methods used to assess risk of bias in the included studies, including details of the tool(s) used, how many reviewers assessed each study and whether they worked independently, and if applicable, details of automation tools used in the process. | Page 7 |
| Effect measures | 12 | Specify for each outcome the effect measure(s) (e.g. risk ratio, mean difference) used in the synthesis or presentation of results. | Page 7 |
| Synthesis methods | 13a | Describe the processes used to decide which studies were eligible for each synthesis (e.g. tabulating the study intervention characteristics and comparing against the planned groups for each synthesis (item #5)). | Page 7 |
|  | 13b | Describe any methods required to prepare the data for presentation or synthesis, such as handling of missing summary statistics, or data conversions. | Page 7 |
|  | 13c | Describe any methods used to tabulate or visually display results of individual studies and syntheses. | Page 7 |
|  | 13d | Describe any methods used to synthesize results and provide a rationale for the choice(s). If meta-analysis was performed, describe the model(s), method(s) to identify the presence and extent of statistical heterogeneity, and software package(s) used. | Page 7 |
|  | 13e | Describe any methods used to explore possible causes of heterogeneity among study results (e.g. subgroup analysis, meta-regression). | Page 7 |
|  | 13f | Describe any sensitivity analyses conducted to assess robustness of the synthesized results. | Page 7 |
| Reporting bias assessment | 14 | Describe any methods used to assess risk of bias due to missing results in a synthesis (arising from reporting biases). | Page 7 |
| Certainty assessment | 15 | Describe any methods used to assess certainty (or confidence) in the body of evidence for an outcome. | Page 7 |
| **RESULTS** | | |  |
| Study selection | 16a | Describe the results of the search and selection process, from the number of records identified in the search to the number of studies included in the review, ideally using a flow diagram. | Page 8 |
|  | 16b | Cite studies that might appear to meet the inclusion criteria, but which were excluded, and explain why they were excluded. | Page 8 |
| Study characteristics | 17 | Cite each included study and present its characteristics. | Page 8 |
| Risk of bias in studies | 18 | Present assessments of risk of bias for each included study. | Page 10 |
| Results of individual studies | 19 | For all outcomes, present, for each study: (a) summary statistics for each group (where appropriate) and (b) an effect estimate and its precision (e.g. confidence/credible interval), ideally using structured tables or plots. | Page 8-10 |
| Results of syntheses | 20a | For each synthesis, briefly summarise the characteristics and risk of bias among contributing studies. | Page 8-10 |
|  | 20b | Present results of all statistical syntheses conducted. If meta-analysis was done, present for each the summary estimate and its precision (e.g. confidence/credible interval) and measures of statistical heterogeneity. If comparing groups, describe the direction of the effect. | Page 8-10 |
|  | 20c | Present results of all investigations of possible causes of heterogeneity among study results. | Page 10 |
|  | 20d | Present results of all sensitivity analyses conducted to assess the robustness of the synthesized results. | Page 10 |
| Reporting biases | 21 | Present assessments of risk of bias due to missing results (arising from reporting biases) for each synthesis assessed. | Page 10 |
| Certainty of evidence | 22 | Present assessments of certainty (or confidence) in the body of evidence for each outcome assessed. | Page 10 |
| **DISCUSSION** | | |  |
| Discussion | 23a | Provide a general interpretation of the results in the context of other evidence. | Page 11 |
|  | 23b | Discuss any limitations of the evidence included in the review. | Page 12 |
|  | 23c | Discuss any limitations of the review processes used. | Page 12 |
|  | 23d | Discuss implications of the results for practice, policy, and future research. | Page 12 |
| **OTHER INFORMATION** | | |  |
| Registration and protocol | 24a | Provide registration information for the review, including register name and registration number, or state that the review was not registered. | Page 3 |
|  | 24b | Indicate where the review protocol can be accessed, or state that a protocol was not prepared. | Page 3 |
|  | 24c | Describe and explain any amendments to information provided at registration or in the protocol. | Psge 3 |
| Support | 25 | Describe sources of financial or non-financial support for the review, and the role of the funders or sponsors in the review. | Page 2 |
| Competing interests | 26 | Declare any competing interests of review authors. | Page 2 |
| Availability of data, code and other materials | 27 | Report which of the following are publicly available and where they can be found: template data collection forms; data extracted from included studies; data used for all analyses; analytic code; any other materials used in the review. | Page 2 |

*From:*  Page MJ, McKenzie JE, Bossuyt PM, Boutron I, Hoffmann TC, Mulrow CD, et al. The PRISMA 2020 statement: an updated guideline for reporting systematic reviews. BMJ 2021;372:n71. doi: 10.1136/bmj.n71. This work is licensed under CC BY 4.0. To view a copy of this license, visit <https://creativecommons.org/licenses/by/4.0/>

**Supplementary Methods**

**Searchkey:**

MEDLINE (via PubMed) and Cochrane Central Register of Controlled Trials (CENTRAL):

(Rectal neoplasms OR "rectal tumour") AND (ELAPE OR extralevator OR extra-levator OR levator OR abdominoperineal OR abdomino-perineal OR abdomino)

Embase:

(Rectal neoplasms OR 'rectal tumour') AND (ELAPE OR extralevator OR extra-levator OR levator OR abdominoperineal OR abdomino-perineal OR abdomino)

**Data items**

In instances where perineal wound dehiscence and/or infection were reported together, the data were collected under the perineal wound complication outcome (1–4). In cases where the perineal abscess was reported separately (5), it was added to the perineal wound infection dataset. In the articles by Vaughan-Shaw and Murashko (6,7), the patients undergoing APE were divided into laparoscopic and open groups; however, as other studies did not report these separately, they have been combined for the present study.

Since some articles lacked hazard ratios (HR) but provided Kaplan-Meier curves, individual patient data (IPD) was estimated from these plots using WebPlotDigitizer (8). Where possible, extracted data (e.g., median time-to-event, survival probabilities, HR estimates) was validated against published values, confirming only minimal differences.

**Detailed Synthesis Method**:

For estimating the pooled MD, if a study reported median, lower, and upper quartiles instead of the mean and SD, we used the Luo and Shi methods (9,10) (as implemented in the used *meta R* package) to estimate the study mean and SD. We assumed that the distribution is not relevantly different from a lognormal distribution regarding these estimations. (We labeled with * the studies on forest plots where this estimation was applied).

For pooling the effect size, pooled OR was calculated by the Mantel-Haenszel method (11,12). The exact Mantel-Haenszel method (without continuity correction) was used to handle zero cell counts (as recommended by Cooper and Sweeting (13,14)). The inverse variance weighting method was used to calculate the pooled MD.

We used a Hartung-Knapp adjustment (15,16) for CIs. To estimate the heterogeneity variance measure (𝜏2), the Paule-Mandel method (17) for a dichotomous and restricted maximum-likelihood estimator for continuous outcomes (or “direct OR”) was used with the Q profile method for the confidence interval (recommended in Harrer et al. and Veroniki et al. (18,19)).

On the forest plots, the t-distribution-based method was used for the CI of MD calculation of individual studies. For OR, normal approximation CI was shown on the forest plot. In the case of 0 cell counts, individual study OR and CI were calculated by adding 0.5 as continuity correction (it was used only for visualization on forest plots).

For time-to-event data in the case of *IPD-based* analysis, we used a mixed-effect Cox model with a single random intercept (also called frailty) model. Gaussian random effects distribution was assumed. The assumption of proportional hazard and random effects distribution, martingale residuals were examined on plots and found acceptable. As an additional sensitivity assessment, we performed a leave-one-out study analysis when comparing APE and ELAPE groups.

For time-to-event data in the case of the *classical* HR pooling method, for pooling the effect size, the inverse variance method was used. We used a Hartung-Knapp adjustment (15,16) for CIs. This adjustment was applied only if it was more conservative than the classical one (as recommended in Jackson et al. (20) as hybrid method 2). To estimate the heterogeneity variance measure (𝜏2), a restricted maximum likelihood method was used with the Q profile method for the confidence interval (recommended by Harrer et al. and Veroniki et al. (18,19)). We used this approach for sensitivity analysis, using the HR values estimated from the IPD data.

We assumed the secondary outcomes (survival probabilities - proportions - in APE and ELAPE groups) are more like each other for different samples within the same study than for samples from different studies. Consequently, we used an additional (independent) random effect in our analysis using a *multilevel* meta-analysis for pooling survival probabilities at a given time point among groups. The survival probabilities with its 95% CI were estimated point-wise using Kaplan-Meier estimates (21) implemented in the *survival* R package. In the multilevel analyses, we used the logit transformation of the calculated proportions. In case when the calculated proportion was 1 (or 0), we did a continuity correction by adding 0.5 to 0 event and the corresponding total sample size value, then calculating the proportion with its exact binomial 95% CI. We used inverse variance weighting with a restricted maximum likelihood method for pooling the proportions. For confidence interval calculation and prediction intervals, we used a t-distribution method. For the test of subgroup differences, a “Cochrane Q” test (an omnibus test) was used between subgroups (18). The null hypothesis was rejected on a 5% significance level. In the case of the 3-level model, we reported the 𝐼2 statistics for each level with its 95% CI as given in (22). We refer to “total 𝐼2” for the sum of the two levels as the total heterogeneity over sampling variance. In the case of multilevel results, we calculated dfbetas (the pooled effect size without the given study expressed in logit scale), Cook’s distances (shows how much the estimated effect size changes, leaving out the given study but taking into consideration how much differ the leaved-out study effect size from the pooled effect size. – the typical threshold for potential influential value is 2) and hat values (the value of the hat matrix without the given study) leaving out 1 study at a time.

The survival curve estimate method was based on the article by Combescure (23) and implemented in the *metaSurvival* package. We used the Greenwoods formula to estimate survival probabilities. As the sample size was 20-100/groups in the studies, to avoid overfitting, we used only a maximum 6 time points to estimate the pooled curve.

**Supplementary Results**

The excluded studies found no significant differences in OS. Carpelan et al. (1) reported mean follow-up times of 3.2 years for ELAPE and 5.8 years for APE, with no significant OS difference (p = 0.8173). Klein et al. (24) found 4-year OS rates of 74% for APE and 77% for ELAPE (p = 0.59). Similarly, Prytz et al. (25) observed no difference in 3-year OS between the two groups.

Carpelan et al. (1) and Klein et al. (24) reported no significant differences in DFS. The former observed mean follow-up times of 3.2 years for ELAPE and 5.8 years for APE, with no significant difference in DFS (p = 0.6311). The latter found 4-year DFS rates of 67% for APE and 66% for ELAPE (p = 0.82).

Carpelan et al.(1) reported LR rates of 7% for ELAPE and 19% for APE (p = 0.2473). In contrast, Prytz et al. (25) found higher 3-year local recurrence rates for ELAPE compared to APE, with a RR of 4.91 (median follow-up: 3.43 years).

**Supplementary Figures and Tables:**

**Table S1:** Reasons for exclusion during full-text assessment

| Nr. | First author | Year | Title | Reason for exclusion |
| --- | --- | --- | --- | --- |
| 1. | Alkhusheh et al | 2016 | Retrospective comparison of the outcomes of extralevator abdominoperineal excision (ELAPE) with abdominoperineal resection (APR) for rectal cancer | Conference abstract |
| 2. | Araujo et al | 2015 | Prone extralevator abdominoperineal excision versus conventional APR for low rectal cancer | Conference abstract |
| 3. | Asplund et al | 2015 | Persistent perineal morbidity is common following abdominoperineal excision for rectal cancer | Does not follow our exact PICO framework, in 252 cases the surgical technique *“remained interminable”.* |
| 4. | Bell et al | 2011 | Short-term outcomes following the extralevator approach to abdominoperineal resection for low rectal cancer | Conference abstract |
| 5. | Bell et al | 2011 | The extralevator approach to abdominoperineal resection for low rectal cancer reduces positive margin rates and intra-operative perforations | Conference abstract |
| 6. | Bianco et al | 2016 | Extralevator with vertical rectus abdominis myocutaneous flap vs. nonextralevator abdominoperineal excision for rectal cancer: the RELAPE trial | Conference abstract |
| 7. | Byrne et al | 2011 | Extralevator abdominoperineal resection results in improved oncological outcome without lengthening stay | Conference abstract |
| 8. | Colov et al | 2014 | Long term oncological and clinical outcomes after extralevatory abdominoperineal resection and conventional abdominoperineal resection for rectal cancer: a nationwide study | Conference abstract |
| 9. | Cooper et al | 2012 | Extra-levator versus standard abdominoperineal excision – is there really a difference? | Conference abstract |
| 10. | Dayal et al | 2011 | Oncological superiority and training potential of ELAPE: best of both worlds? | Conference abstract |
| 11. | Dijkstra et al | 2020 | A low incidence of perineal hernia when using a biological mesh after extralevator abdominoperineal excision with or without pelvic exenteration or distal sacral resection in locally advanced rectal cancer patients | “*one patient was treated for a rectal gastrointestinal stromal tumour (GIST).”* |
| 12. | Dinnewitzer et al | 2012 | Prone perineal approach for extralevator abdomino-perineal resection versus conventional abdomino-perineal resection – Comparison of short term outcome | Conference abstract |
| 13. | Duboczki et al | 2019 | Extralevator abdominoperineal excision can be an effective surgical treatment in advanced, low rectal adenocarcinomas – a single institution experience | Conference abstract |
| 14. | Gao et al | 2019 | Comparison of short-term outcomes of 3D laparoscopic extralevator abdominoperineal excision versus conventional abdominoperineal resection in low rectal cancer. | Conference abstract |
| 15. | Gash et al | 2014 | No observable difference in operative oncological outcomes between extra-levator versus standard abdominoperineal excision | Correspondence |
| 16. | Grover et al | 2012 | Evolution of abdominoperineal excision for distal rectal cancer: analysis of the short-term outcomes of the extralevator and conventional approach over a 5-year period in a large tertiary referral centre | Conference abstract |
| 17. | Habr-Gama et al | 2017 | Extralevator Abdominal Perineal Excision Versus Standard Abdominal Perineal Excision: Impact on Quality of the Resected Specimen and Postoperative Morbidity | Recurrent rectal cancer cases added also. |
| 18. | Hayes et al | 2013 | Comparison of outcomes between ELAPE and traditional APER in low rectal cancer | Conference abstract |
| 19. | He et al | 2023 | Comparison of the clinical efficacy of laparoscopic extralevator abdominoperineal excision (ELAPE) and non‐ELAPE for low rectal cancer | Article doesn’t state what non-ELAPE is, does it include only APR cases or other also. |
| 20. | How et al | 2013 | A comparison of short term outcomes between conventional abdominoperineal excision and extralevator abdominoperineal excision in a prospective multicentre study | Conference abstract |
| 21. | Johnson et al | 2014 | Comparison of short-term outcomes of extralevator abdominoperineal excision and standard abdominoperineal excision for rectal cancer | Conference abstract |
| 22. | Jones et al | 2015 | LOREC registry – interim analysis of operative technique and perineal wound healing outcomes after abdomino-perineal excision | Conference abstract |
| 23. | Jones et al | 2015 | The LOREC APE registry – Operative technique, oncological outcome and perineal wound healing after abdominoperineal excision | “*non-ELAPE procedures , comprising 76 ‘standard’ APE, nine intersphincteric, two ischioanal and seven procedures of unspecified type*.” |
| 24. | Kamali et al | 2016 | Patient’s Quality of Life After Standard Versus Extra-Levator Abdominoperineal Excision: A Prospective Case Control Study | Conference abstract |
| 25. | Kamali et al | 2016 | Extra Levator Versus Standard Abdominoperineal Excision for Rectal Cancer: Is There Any Oncological Benefit? | Conference abstract |
| 26. | Lehtonen et al | 2018 | Oncological outcomes before and after the ELAPE-era in rectal cancer patients treated with abdominoperineal excision in a single center, high volume unit | “*The first group (A) of 71 patients, had operations during 2000-2007 before the adoption of ELAPE. The other group (B) of 135 patients had operations in 2008-2014. This second cohort B (2008-2014) consisted of both conventionally operated patients (n=37, 27.4%) and ELAPE-operated patients with threatened margins (n=98, 72.6%).”* |
| 27. | Liu et al | 2019 | Improvement in the quality of lymph node harvests using extra-levator abdominoperineal excisions compared with conventional techniques | Conference abstract |
| 28. | Martijnse et al | 2011 | Improved Oncological Outcome After Modified Extralevator Abdominoperineal Excision in Low Rectal Cancer Patients | Conference abstract |
| 29. | Mege et al | 2023 | Supine bottom‐up extralevator abdominoperineal excision for anorectal adenocarcinoma is not inferior to standard approach and may be thus safely performed | In the description, the “supine bottom-up extralevator abdominoperineal excision” seems like a modified procedure, not the standard ELAPE |
| 30. | Mejias et al | 2017 | Prophylactic mesh in extralevator abdominoperinealresection (elape) for low rectal cancer | Conference abstract |
| 31. | Miah et al | 2015 | Neo-adjuvant chemo-DXT and ELAPR: the gold standard in the treatment of low rectal cancer | Conference abstract |
| 32. | Musbahi et al | 2016 | Extralevator Versus Standard Abdominoperineal Excision: Does Radiotherapy Play A Part in Wound Breakdown? | Conference abstract |
| 33. | Musters et al | 2014 | Perineal wound problems after abdominoperineal resection for rectal cancer; a two-institutional experience in the era of intensified oncological treatment | Conference abstract |
| 34. | Musters et al | 2014 | Perineal wound healing after abdominoperineal resection for rectal cancer: a two-centre experience in the era of intensified oncological treatment | It compares different time periods, when not only ELAPE and APER was performed but also intersphincteric resection. Subgroup analysis is not possible. |
| 35. | Ocampo et al | 2012 | Factors which influenced waisting, perforation, and circumferential resection margin positivity in the abdominoperineal resection specimen | Conference abstract |
| 36. | Pai et al | 2014 | Extralevator adomino perineal resection-Short-term oncological and clinical outcomes in comparision with conventional procedure | Conference abstract |
| 37. | Pai et al | 2015 | Selective extra levator versus standard abdominoperineal resection – experience from a tertiary care centre | Conference abstract |
| 38. | Paraoan et al | 2014 | Does national laparoscopic and LoRec cancer training deliver improved outcomes in low rectal cancer? | Conference abstract |
| 39. | Patel et al | 2013 | Circumferential resection margin involvement in standard versus extralevator abdominoperineal excision of rectal cancer | Conference abstract |
| 40. | Patel et al | 2013 | Clinical outcomes after abdominoperineal excision of rectum: standard versus ELAP approach | Conference abstract |
| 41. | Patel et al | 2013 | Site of circumferential resection margin involvement in standard versus extralevator abdominoperineal excision of rectal cancer | Conference abstract |
| 42. | Polkowoski et al | 2014 | Extralevator abdomino-perineal excision (ELAPE) or abdomino-sacral amputation of the rectum (ASAR): Revitalized approach for low rectal carcinoma described by Tadeusz Koszarowski in the 50s | Correspondence |
| 43. | Prytz et al | 2013 | Swedish patients operated with extralevator APE (ELAPE) | Conference abstract |
| 44. | Prytz et al | 2014 | Oncological results of extralevator abdomnioperineal excision (ELAPE) in a Swedish, national cohort | Conference abstract |
| 45. | Prytz et al | 2018 | Association between operative technique and intrusive thoughts on health-related Quality of Life 3 years after APE/ ELAPE for rectal cancer: results from a national Swedish cohort with comparison with normative Swedish data | Does not report any outcome of interest. |
| 46. | Sharpe et al | 2016 | Laparoscopic Versus Open Abdominoperineal Excision: Is There Any Impact on Patient Quality of Life? | Conference abstract |
| 47. | Shen et al | 2015 | Prospective controlled study of the safety and oncological outcomes of ELAPE procure with definitive anatomic landmarks versus conventional APE for lower rectal cancer | Preliminary results reported. We have the final study included. |
| 48. | Sutaria et al | 2014 | Extralevator APER is not associated with increased morbidity compared with standard APER | Conference abstract |
| 49. | Thirkettle et al | 2015 | Role of technique of abdomino-perineal excision of rectum on perineal complications and interventions – our experience | Conference abstract |
| 50. | Wang et al | 2011 | Preliminary Experience of Radical Surgery for Locally Advanced Lower Rectal Cancer − Cylindrical Versus Conventional Abdominoperineal Resection | Conference abstract |
| 51. | Wang et al | 2012 | Preliminary experience of cylindrical abdominoperineal resection with pelvic floor reconstruction using human acellular dermal matrix for locally advanced lower rectal cancer | Conference abstract |
| 52. | West et al | 2008 | Evidence of the Oncologic Superiority of Cylindrical Abdominoperineal Excision for Low Rectal Cancer | Possible overlap in population with the included article of West el at, 2010 |
| 53. | Wu et al | 2013 | Extralevator abdominoperineal excision versus traditional abdominoperineal excision in the treatment of low rectal cancer | Possible overlap in population with the included article of Zhang et al 2014 |
| 54. | Xiao et al | 2014 | Feasibility and short-outcomes of laparoscopic extralevator abdominoperineal excision without changing position for distal rectal cancer | Possible overlap in population with the included article of Zhang et al 2014 |
| 55. | Zhang et al | 2012 | Standard abdominoperineal excision can achieve similar circumferential resection margin as more invasive extralevator technique | Conference abstract |

**Table S2.** Baseline characteristics of the enrolled studies

| Nr. | First author | Year | Country | ELAPE | | | | APE | | | |
| --- | --- | --- | --- | --- | --- | --- | --- | --- | --- | --- | --- |
|  |  |  |  | Nr. of patients (%F) | Age (years) | Tumor height (cm) | Oncological therapy | Nr. of patients (%F) | Age | Tumor height (cm) | Oncological therapy |
| 1. | Aggarwal(26) | 2018 | India | 21 (38,1) | 50 (20-68)* | NA | NCRT –100% | 50 (NA) | NA | NA | NCRT – 100% |
| 2. | Asplund(27) | 2012 | Sweden | 79 (44) | 67 (35 – 89)* | 4(0-8)* | NRT – 75,9%  NCRT -18.9% | 79 (30) | 68 (38 – 85)* | 4 (0-10)* | NRT – 83,5%  NCRT – 6,3% |
| 3. | Bianco(28) | 2016 | International | 17 (52,4) | 60 (90)† | 2 (2-3)¶ | NCRT –100% | 17 (35,3) | 64 (12) † | 2 (1,75-4) ¶ | NCRT –100% |
| 4. | Carpelean (1) | 2018 | FInland | 42 (35,7) | 61 (2)§ | NA | NCRT – 66%  NRT – 26,1%  CT – 73,8% | 27 (25,6) | 67 (2) § | NA | NCRT -70,3%  NRT – 22,2%  CT – 62,96% |
| 5. | Cesar (29) | 2018 | Brazil | 22 (45) | 58 ‡ | 0 (from dentate line) | NCRT –100% | 67 (39) | 57 ‡ | 1 (from dentate line) | NCRT –100% |
| 6. | Colov (30) | 2016 | Denmark | 245 (37) | 68 (59-74) ¶ | 0-5:90%  6-10:10% | NCRT – 71% | 200 (37) | 69 (63-77) ¶ | 0-5:69%  6-10:31% | NCRT – 41% |
| 7. | Danilov (31) | 2020 | Russia | 62 (42) | 60,3 (7,8)† | 3,7 (1,2) † | NCRT – 66%  CT – 38,7% | 30 (40) | 62,4 (8,2)† | 3,6 (1,2) † | NCRT -63,3%  CT – 40% |
| 8. | Dsouza (32) | 2020 | India | 57 (40,4) | 46,37‡ | NA | NCRT -96,5% | 138 (31,9) | 46,29‡ | NA | NCRT -97,8% |
| 9. | Güven (33) | 2019 | Turkey | 42(41) | 58 (7,1) † | 3,4 (1,1) † | NCRT –100% | 62(45) | 59,4 (4,2) † | 3,2 (0,7) † | NCRT-100% |
| 10. | Han (34) | 2012 | China | 35 ( 42,8) | 63 (44-81)* | 3 (2-7)* | NCRT -28,5% | 32 (34,3) | 68 (32-84)* | 3(2-5)* | NCRT -28,1% |
| 11. | Hanif (35) | 2016 | UK | 24 (38) | 68 (37-87) * | NA | NRT – 20,8% | 48 (31) | 69 (41-82) * | NA | NRT – 18,7% |
| 12. | How(36) | 2013 | Ireland | 10 (40) | 58 (48-83)* | 4,4 ‡ | NCRT – 80%  NRT – 20% | 10(20) | 66 (51-79)* | 4,7 ‡ | NCRT – 90%  NRT – 10% |
| 13. | Kamali (37) | 2017 | UK | 27 (33,3) | 67,8‡ | NA | NCRT -81,5% | 20 (14,2) | 66,9‡ | NA | NCRT -71,4% |
| 14.  15. | Klein (24,38) | 2015  2016 | Denmark | 301 (36) | 68 (60-75) ¶ | 0-5:88,7%  6-10:8,6%  >10:0,6% | NCRT – 70% | 253 (36) | 69 (63-77) ¶ | 0-5:62,8%  6-10:32%  >10:2,7% | NCRT – 45% |
| 16. | Liu (39) | 2020 | UK | 30 (26,6) | 68 ‡ | NA | NCRT -53,3% | 30(43,3) | 67,8 ‡ | NA | NCRT -46,6% |
| 17. | Murashko (7) | 2017 | Russia | 42 (57) | 60,3(44-73) * | NA | NRT – 59,5%  NCRT – 7% | 48 (31) | 69 (41-82) * | NA | NRT – 45,8%  NRCT – 8% |
| 18. | Nessar (40) | 2016 | Turkey | 25 (32) | 60,3 (13,%)† | NA | NCRT – 36% | 56 (34) | 56,8 (14,1) † | NA | NCRT -10,7% |
| 19. | Ortiz (2) | 2014 | Spain | 457 (28,4) | <65:35%  65-75:30%  >75:35% | 0-5:96%  6:4% | NCRT – 76% | 457 (29,9) | <65:34%  65-75:30%  >75:35% | 0-5:95%  6:5% | NCRT -75,7% |
| 20. | Othman (5) | 2020 | Egypt | 20 | NA | 2 (0.1-4)* | NCRT – 85% | 20 | NA | 3 (1-4)* | NCRT – 85% |
| 21. | Pai (3) | 2016 | India | 42(11,9) | 46 ¥ | NA | NCRT – 88% | 78 (32) | 47 ¥ | NA | NCRT -79,5% |
| 22. | Papp (41) | 2020 | Hungary | 38 (34,2) | 66,5(33-85)* | 2(0-8)* | NCRT – 60% | 35(42,9) | 68 (39-82)* | 2 (0-10)* | NCRT -68,5% |
| 23. | Park (42) | 2016 | Korea | 13 (30,7) | 55 (48-58) ¶ | 0-5:92,3%  >5:7,7% | NCRT -76,9% | 26 (46,15) | 65(58-76) ¶ | 0-5:92,3%  >5:7,7% | NCRT -96,1% |
| 24. | Perdawood (43) | 2014 | Denmark | 68 (34) | 68 (42-85)* | 4(1-6)* | NCRT -85,3% | 39 (31) | 69 (58-88)* | 3(1-6)* | NCRT -48,7% |
| 25.  26. | Prytz (25,44) | 2014  2016 | Sweden | 518 (40) | 68 (61-76) ¶ | 3,4 ‡ | NCRT -30,6%  NRT – 88% | 209 (44) | 71 (63-79) ¶ | 6,6 ‡ | NCRT -15,3%  NRT – 68,9% |
| 27. | Qi (45) | 2019 | China | 53(39,6) | 60,2 (7,96) † | 3,6 (1,1) † | NCRT –100%  CT – 37,7% | 58(43,1) | 57,3 (11,1) † | 3,2 (1,06) † | NCRT -17,2%  CT – 43,1% |
| 28. | Seshadri (4) | 2017 | India | 10 (30) | 55,8 (11,7) † | 1,4 (1,07) † | NCRT -100% | 10 (30) | 56,6 (11,9) † | 1,3 (0,8) † | NCRT -100% |
| 29. | Shen (46) | 2019 | China | 106 (36,8) | 60,7(52-70) ¶ | 3,1 (2-4) ¶ | NCRT-42,4%  CT – 43,4%  RT – 8,49% | 88 (42) | 63,1(56-73) ¶ | 3,7(2,8-4,9) ¶ | NCRT -28,4%  CT – 31,8%  RT – 6,8% |
| 30. | Stelzner (47) | 2011 | Germany | 28 (32,1) | 66 (44-79)* | 2,5(0-8)* | NCRT –100% | 46 (19,6) | 64 (44-82)* | 3(0-9)* | NCRT – 100% |
| 31. | Stelzner (48) | 2016 | Germany | 36 (36,1) | 65 (44-79)* | 2(0-5)* | NCRT -88,8%  NRT – 8,3%  NCT -2,7%  CT – 36,1% | 36 (9) | 64 (45-82) | 4(0-6)* | NCRT -97,2%  NRT – 0%  NCT – 2,7%  CT – 33,3% |
| 32. | Ulusoy (49) | 2022 | Skopje | 15 (33,3) | 59,7  (17) † | NA | NA | 52 (36,5) | 65,5 (10) † | NA | NA |
| 33. | Van Oostendorp (50) | 2020 | International | 17(35,3) | 62  (14) † | NA | NA | 8 (12,5) | 70,3 (7,1) † | NA | NA |
| 34. | Vaughan-Shaw (6) | 2012 | UK | 16 (56,25) | 71 (49-88)* | NA | NCRT -56,2%  NRT - 43,8% | 10 (50)  10 (20) | 72 (52-87)*  72,5(46-89)* | NA | NCRT – 35%  NRT – 45% |
| 35. | Wang (34) | 2015 | China | 23 (46,8) | 57,3 (12,2) † | 3  (1,3) † | NCRT -21,7%  RT – 39,13% | 25 (52) | 56,8 (11,9) † | 3  (1,45) † | NCRT – 20%  RT – 44% |
| 36. | Wang (51) | 2022 | China | 94 (44,6) | 59,6 (11,9) † | NA | NCRT -47,8% | 83 (55,4) | 58,3 (10,8) † | NA | NCRT -50,6% |
| 37. | West (52) | 2010 | International | 176 (31,8) | 66 (58-73) ¶ | 1,5(0,2-2) ¶ | RT – 73,86%  CT – 47,72% | 124 (29,8) | 68 (57 – 75) ¶ | 2(0,3-3) ¶ | RT – 72,58%  CT – 38,7% |
| 38. | Zhang (53) | 2022 | China | 68 (36,8) | 61,3 (11,5) † | 3 (2-3,5)* | NCRT -45,6%  CT- 70,5% | 46 (34,8) | 64,8 (11,1) † | 3 (2-4)* | NCRT -19,5%  CT – 60,8% |

Grey filling means a study is a randomized controlled study; ELAPE: extralevator abdominoperineal excision; APE: abdominoperineal excision; NA: not available; %F: percentage of female patients; NCRT: neoadjuvant chemoradiotherapy; NRT: neoadjuvant radiotherapy; NCT: neoadjuvant chemotherapy; RT: adjuvant radiotherapy; CT: adjuvant chemotherapy; * median (range min-max); † mean (SD); ¶ median (IQR min-max); § mean (SEM); ‡ mean; ¥ median

**Table S3:** TNM Staging in Studies Included in the OS Analysis

| Study | **pTNM** | | | | | **cTNM** | | | | |
| --- | --- | --- | --- | --- | --- | --- | --- | --- | --- | --- |
|  |  | Nr. of events ELAPE | Nr. of patients ELAPE | Nr. of events APE | Nr. of patients APE |  | Nr. of events ELAPE | Nr. of patients ELAPE | Nr. of events APE | Nr. of patients APE |
| **Han et al, 2012** | T3 N0 M0 | 10 | 35 | 10 | 32 | T3 N0 M0 | 5 | 35 | 6 | 32 |
|  | T3 N1-2 M0 | 20 | 35 | 14 | 32 | T3 N1-2 M0 | 19 | 35 | 14 | 32 |
|  | T4 N1-2 M0 | 5 | 35 | 7 | 32 | T4 N1-2 M0 | 11 | 35 | 12 | 32 |
| **Klein et at, 2016** (NA, extracted from Klein et al 2015) | T0 | 23 | 301 | 15 | 253 | NA | | | | |
|  | T1 | 18 | 301 | 20 | 253 |  |  |  |  |  |
|  | T2 | 82 | 301 | 94 | 253 |  |  |  |  |  |
|  | T3 | 148 | 301 | 103 | 253 |  |  |  |  |  |
|  | T4 | 28 | 301 | 20 | 253 |  |  |  |  |  |
|  | Unknown | 2 | 301 | 1 | 253 |  |  |  |  |  |
|  | N0 | 212 | 301 | 178 | 253 |  |  |  |  |  |
|  | N1 | 53 | 301 | 43 | 253 |  |  |  |  |  |
|  | N2 | 36 | 301 | 32 | 253 |  |  |  |  |  |
| **Qi et al, 2019** | T0 | 7 | 53 | 0 | 58 | T1 | 3 | 53 | 2 | 58 |
|  | T1 | 1 | 53 | 1 | 58 | T2 | 23 | 53 | 20 | 58 |
|  | T2 | 19 | 53 | 19 | 58 | T3 | 20 | 53 | 25 | 58 |
|  | T3 | 17 | 53 | 26 | 58 | T4 | 7 | 53 | 11 | 58 |
|  | T4 | 9 | 53 | 12 | 58 | N0 | 25 | 53 | 21 | 58 |
|  | N0 | 33 | 53 | 31 | 58 | N1 | 16 | 53 | 24 | 58 |
|  | N1 | 11 | 53 | 12 | 58 | N2 | 12 | 53 | 13 | 58 |
|  | N2 | 9 | 53 | 15 | 58 |  |  |  |  |  |
| **Shen et al, 2019** | T0-T2 | 39 | 106 | 30 | 88 | T1-T2 | 23 | 106 | 23 | 88 |
|  | T3-T4 | 67 | 106 | 58 | 88 | T3-T4 | 79 | 106 | 57 | 88 |
|  | N0 | 60 | 106 | 56 | 88 | N0 | 35 | 106 | 39 | 88 |
|  | N+ | 46 | 106 | 32 | 88 | N+ | 65 | 106 | 39 | 88 |
|  | M0 | 94 | 106 | 83 | 88 | M0 | 96 | 106 | 84 | 88 |
|  | M+ | 12 | 106 | 5 | 88 | M+ | 10 | 106 | 4 | 88 |
| **Stelzner et al, 2011** | ypN0 | 19 | 28 | 32 | 46 | T1-3 | 3 | 28 | 10 | 46 |
|  | ypN+ | 9 | 28 | 14 | 46 | T4 | 25 | 28 | 36 | 46 |
|  |  |  |  |  |  | M0 | 27 | 28 | 36 | 46 |
|  |  |  |  |  |  | M1 | 1 | 28 | 10 | 46 |
| **Wang et al, 2015** | T3N0M0 | 4 | 23 | 5 | 25 | T3N0M0 | 7 | 23 | 8 | 25 |
|  | T4N0M0 | 1 | 23 | 2 | 25 | T4N0M0 | 3 | 23 | 5 | 25 |
|  | T3N1-2M0 | 12 | 23 | 11 | 25 | T3N1-2M0 | 9 | 23 | 9 | 25 |
|  | T4N1-2M0 | 6 | 23 | 7 | 25 | T4N1-2M0 | 4 | 23 | 3 | 25 |
| **Danilov et al, 2020** | T0 | 3 | 62 | 1 | 30 | T1 | 2 | 62 | 1 | 30 |
|  | T1 | 1 | 62 | 1 | 30 | T2 | 26 | 62 | 12 | 30 |
|  | T2 | 24 | 62 | 11 | 30 | T3 | 24 | 62 | 13 | 30 |
|  | T3 | 22 | 62 | 10 | 30 | T4 | 10 | 62 | 5 | 30 |
|  | T4 | 12 | 62 | 7 | 30 | N0 | 31 | 62 | 15 | 30 |
|  | N0 | 38 | 62 | 19 | 30 | N1 | 23 | 62 | 11 | 30 |
|  | N1 | 18 | 62 | 9 | 30 | N2 | 8 | 62 | 4 | 30 |
|  | N2 | 6 | 62 | 2 | 30 |  |  |  |  |  |

**Figure S1:** Forest plots representing overall survival following ELAPE vs. APE using “classical” analysis.


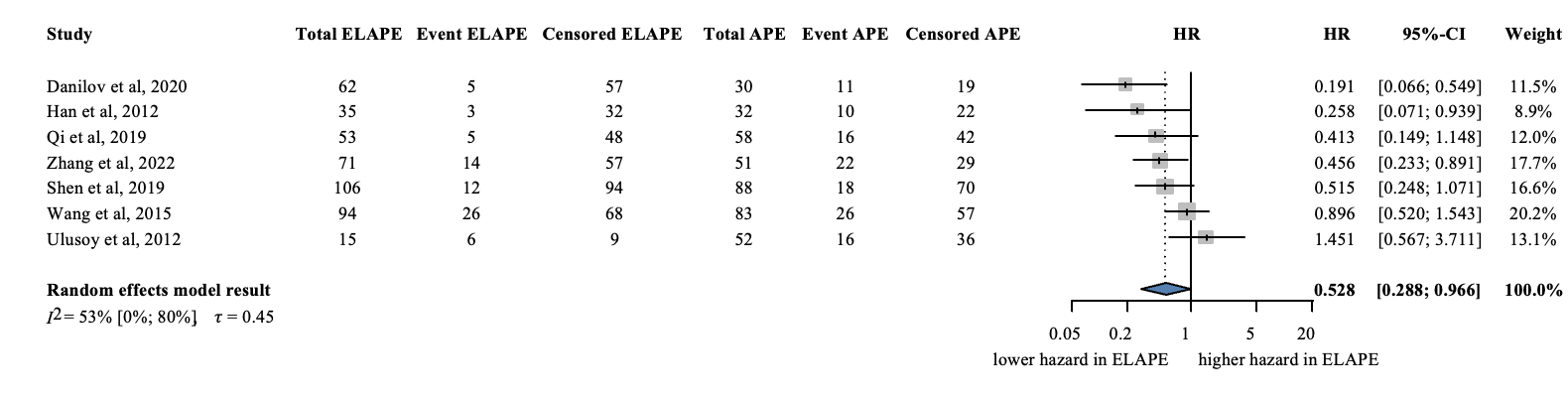


**Figure S2.** Estimated survival probabilities at given times following ELAPE vs. APE

**
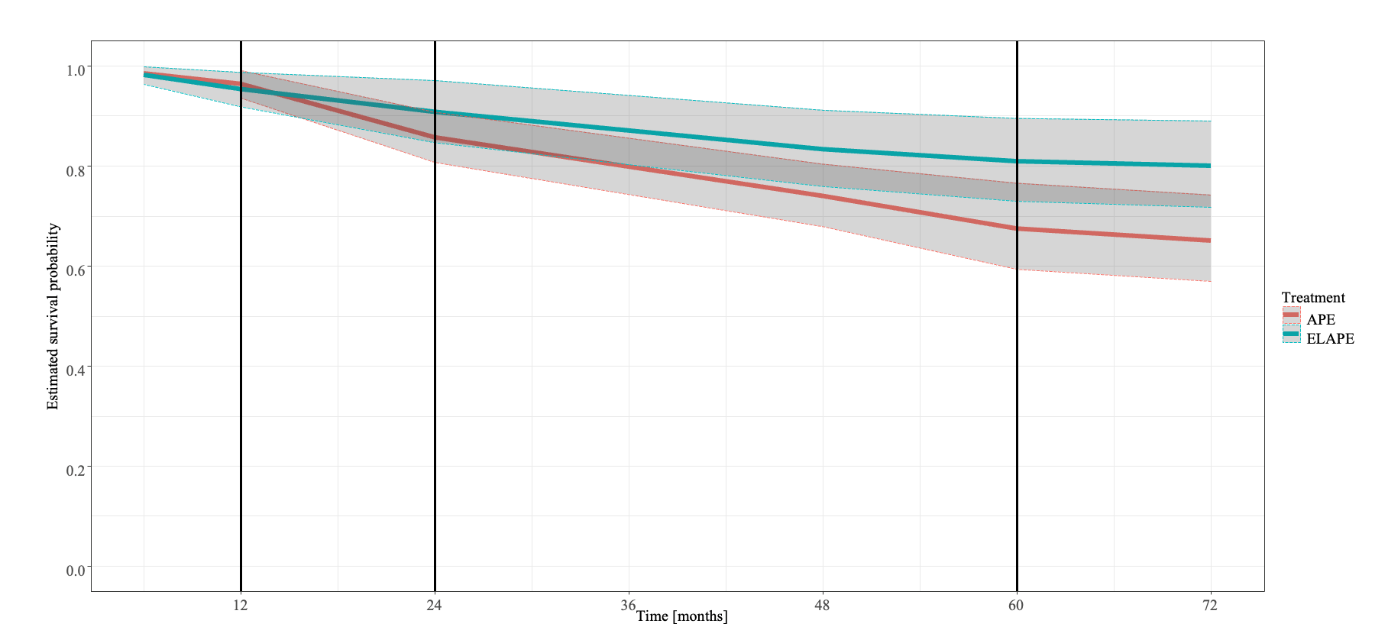
**

**Figure S3:** Estimated survival probability at 12 months following extralevator abdominoperineal excision vs. standard abdominoperineal excision


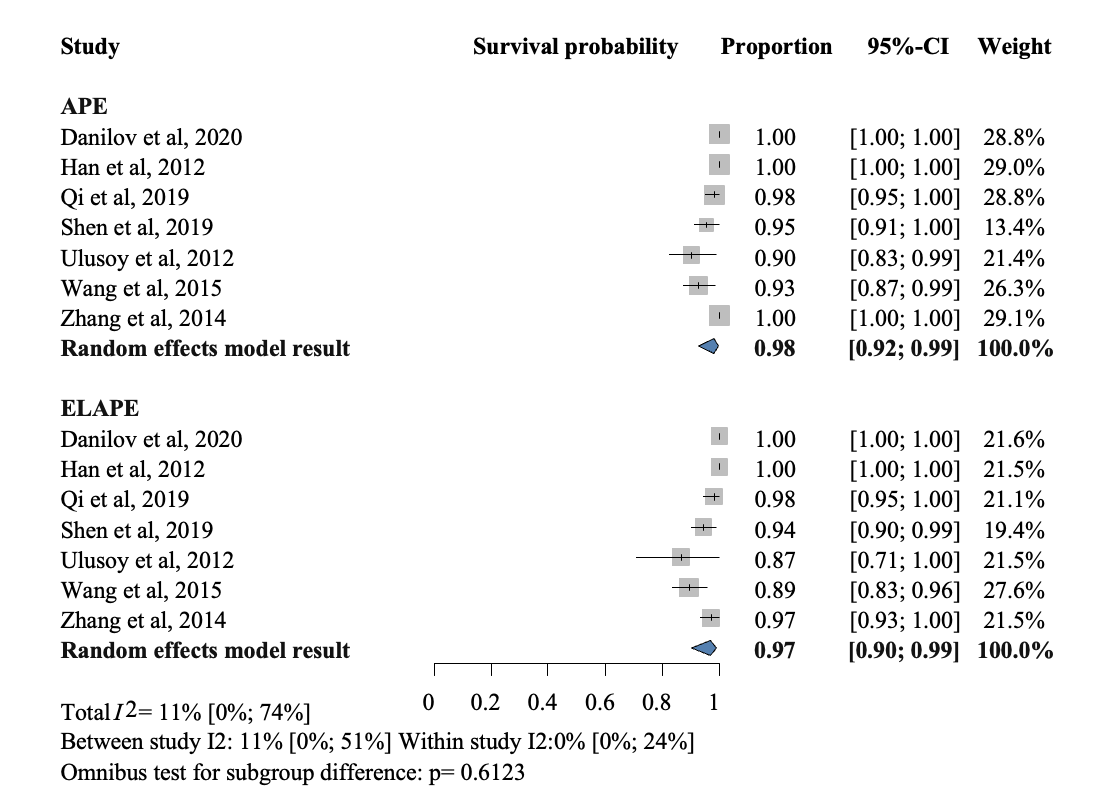
**Figure S4:** Estimated survival probability at 24 months following extralevator abdominoperineal excision vs. standard abdominoperineal excision
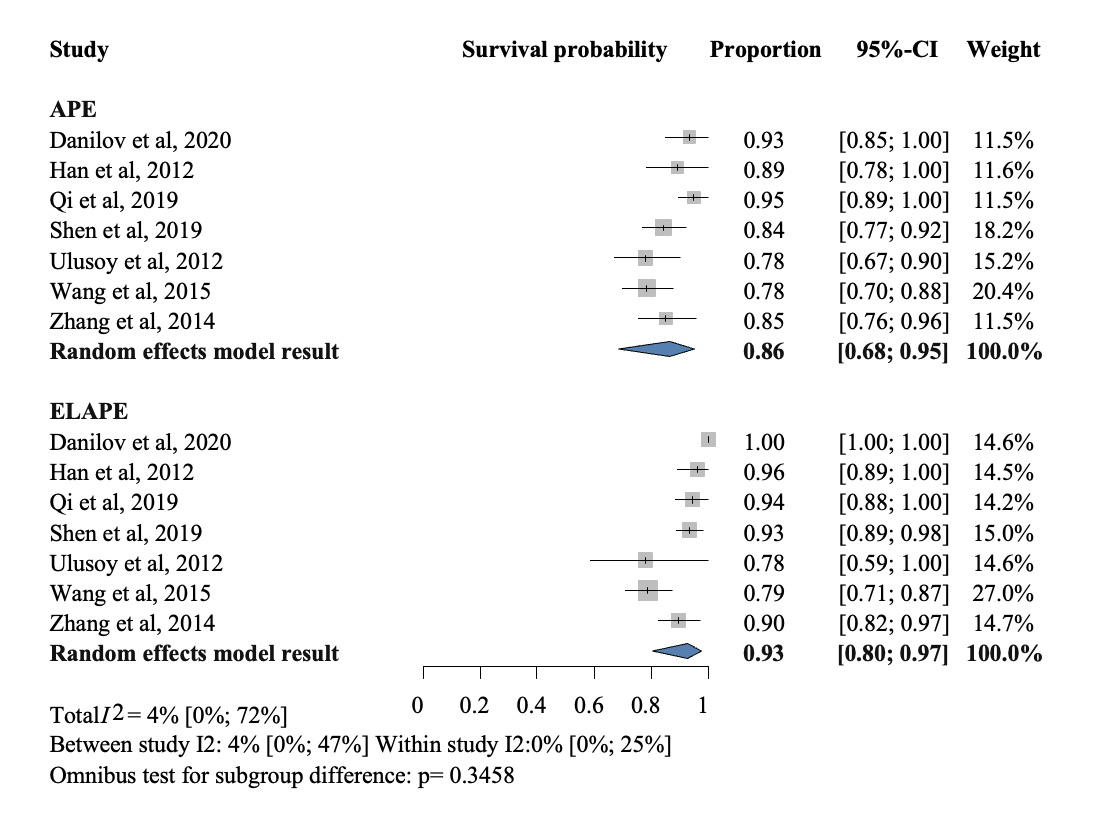
**Figure S5:** Estimated survival probability at 60 months following extralevator abdominoperineal excision vs. standard abdominoperineal excision
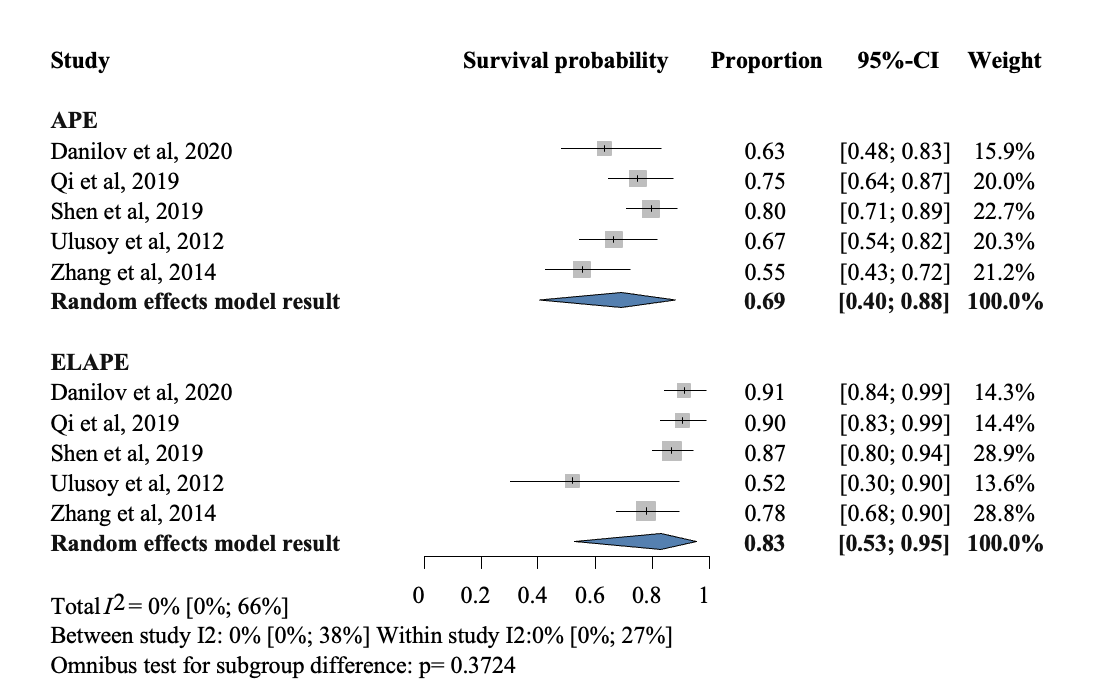


**Figure S6:** Kaplan-Meier curves for overall survival, showing individual study-specific survival probabilities for extralevator abdominoperineal excision and standard abdominoperineal excision.


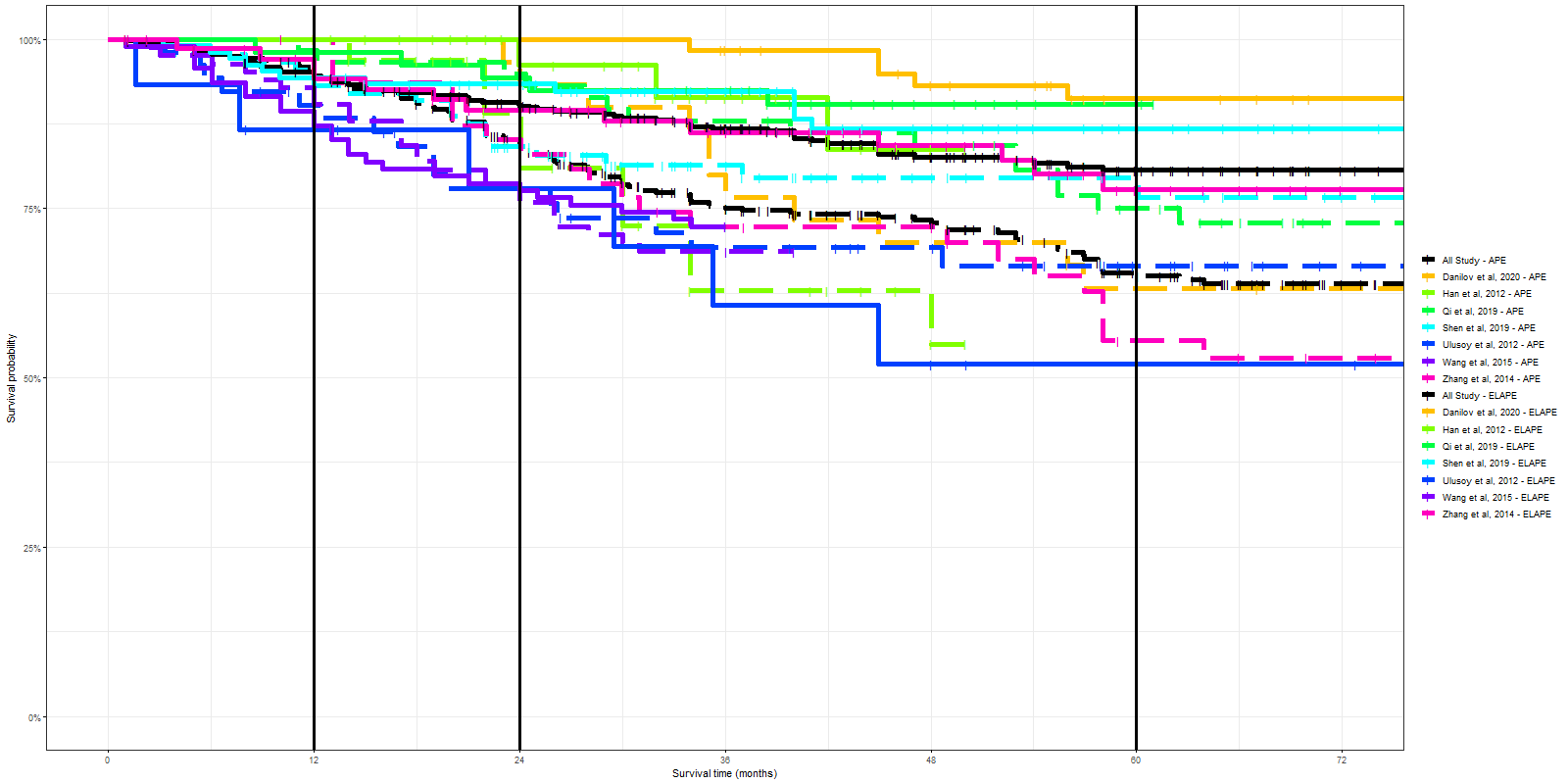


**Figure S7:** Forest plot representing exploratory pooled overall survival effects for ELAPE vs APE after integrating the two registry studies with the previously analyzed data **
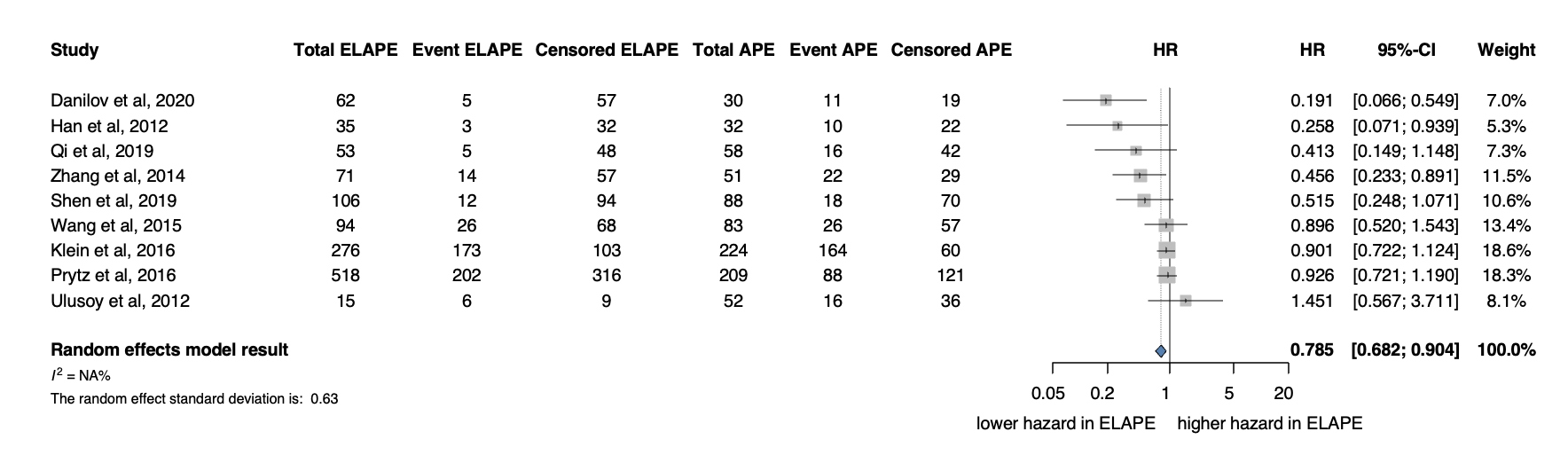
**

**Figure S8:** Forest plots representing disease-free survival following ELAPE vs. APE using “classical” analysis.

**
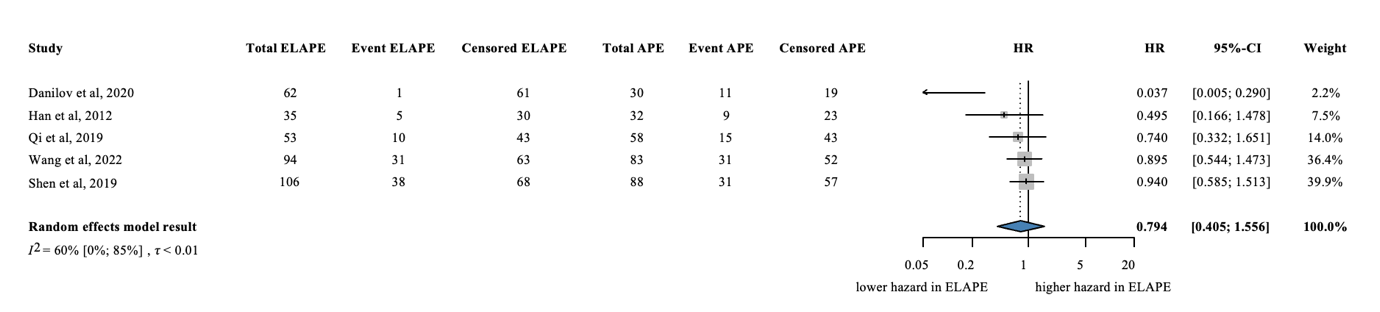
**

**Figure S9.** Estimated DFS probabilities at given times following ELAPE vs. APE **
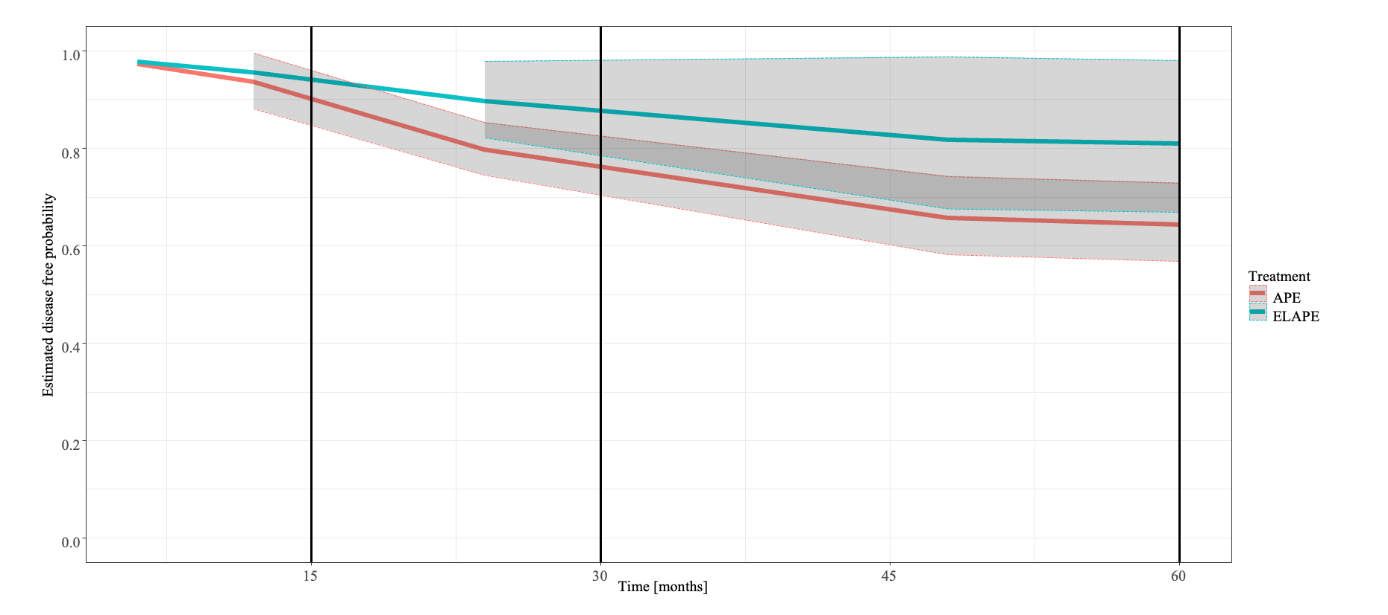
**

**Figure S10:** Estimated disease-free survival probability at 12 months following extralevator abdominoperineal excision vs. standard abdominoperineal excision


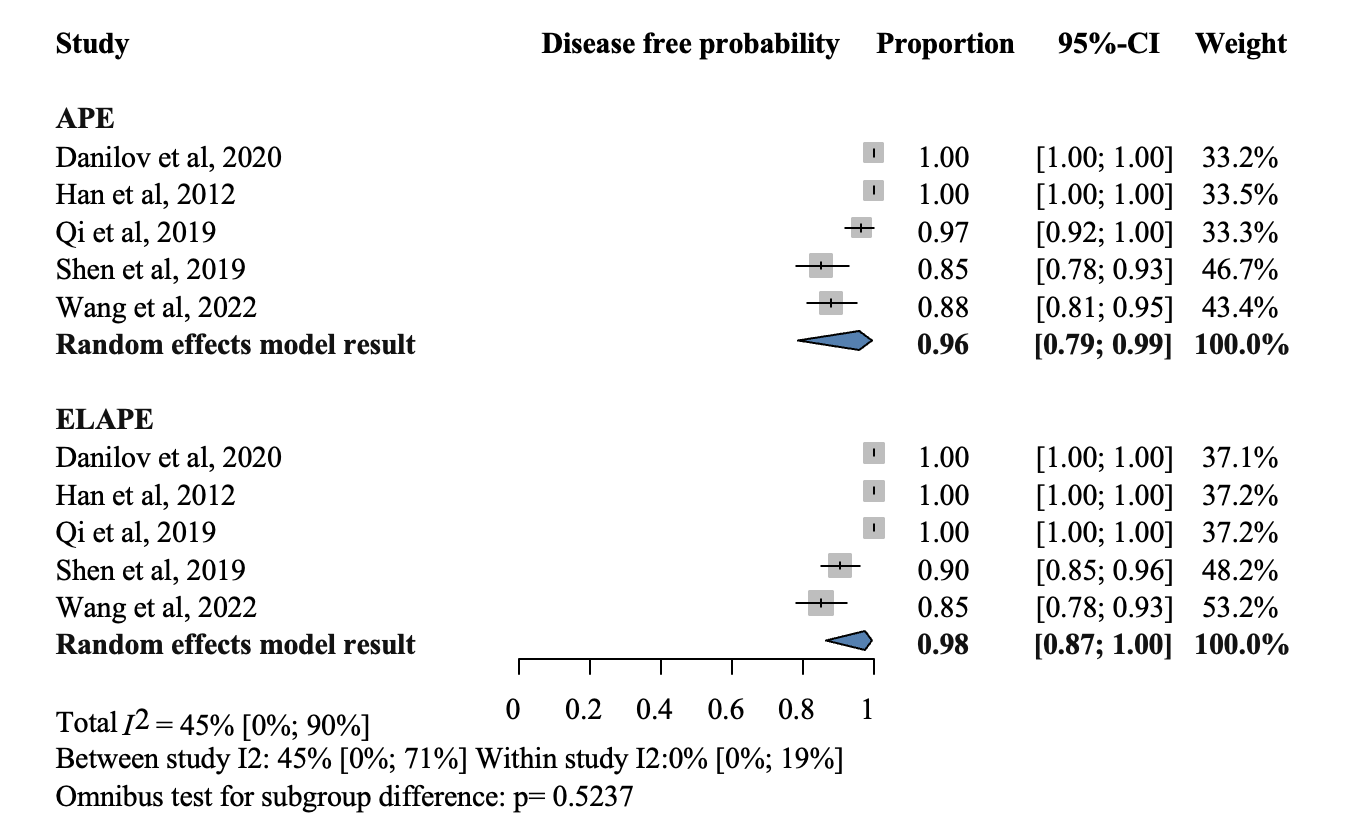


**Figure S11:** Estimated disease-free survival probability at 24 months following extralevator abdominoperineal excision vs. standard abdominoperineal excision


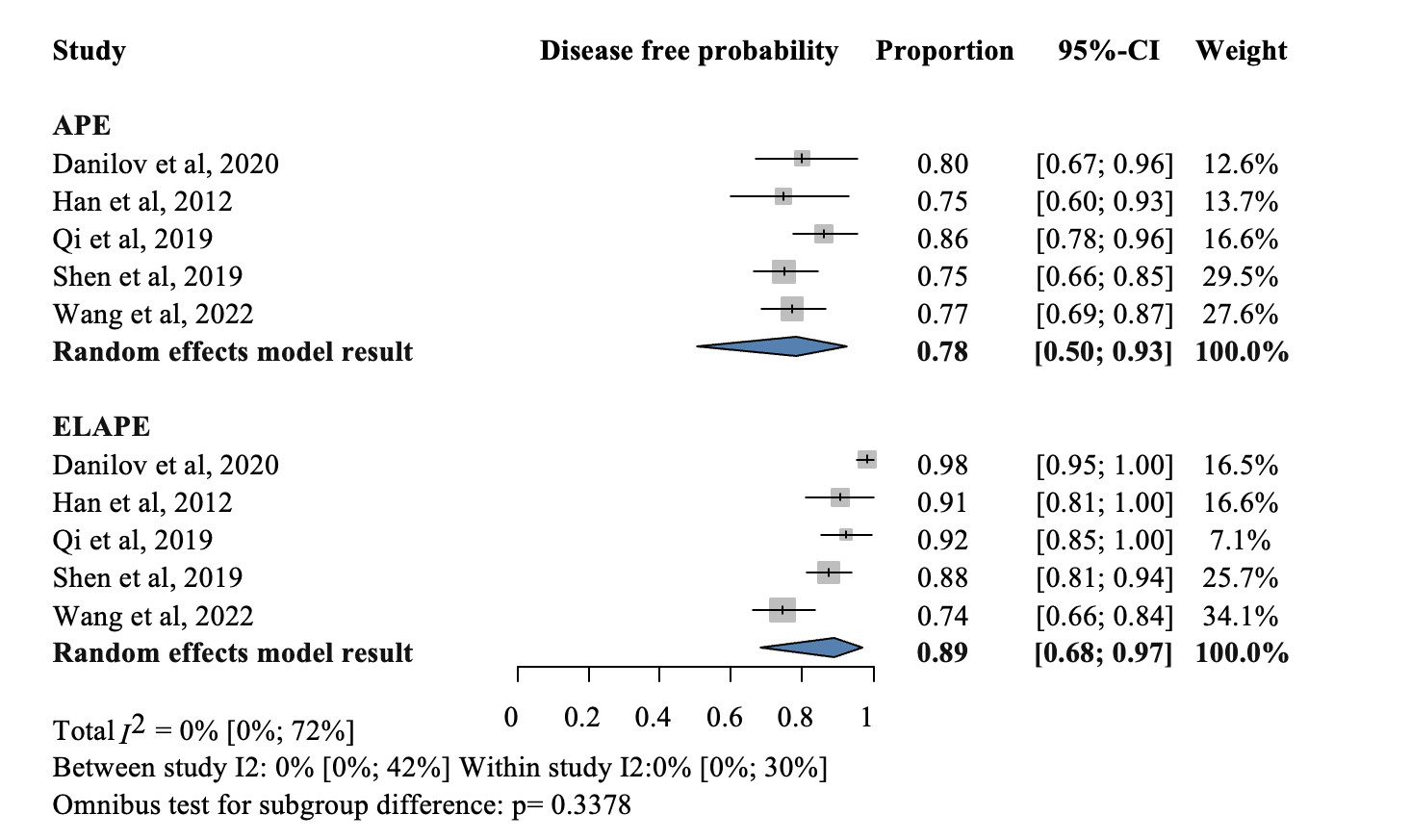


**Figure S12:** Estimated disease-free survival probability at 60 months following extralevator abdominoperineal excision vs. standard abdominoperineal excision


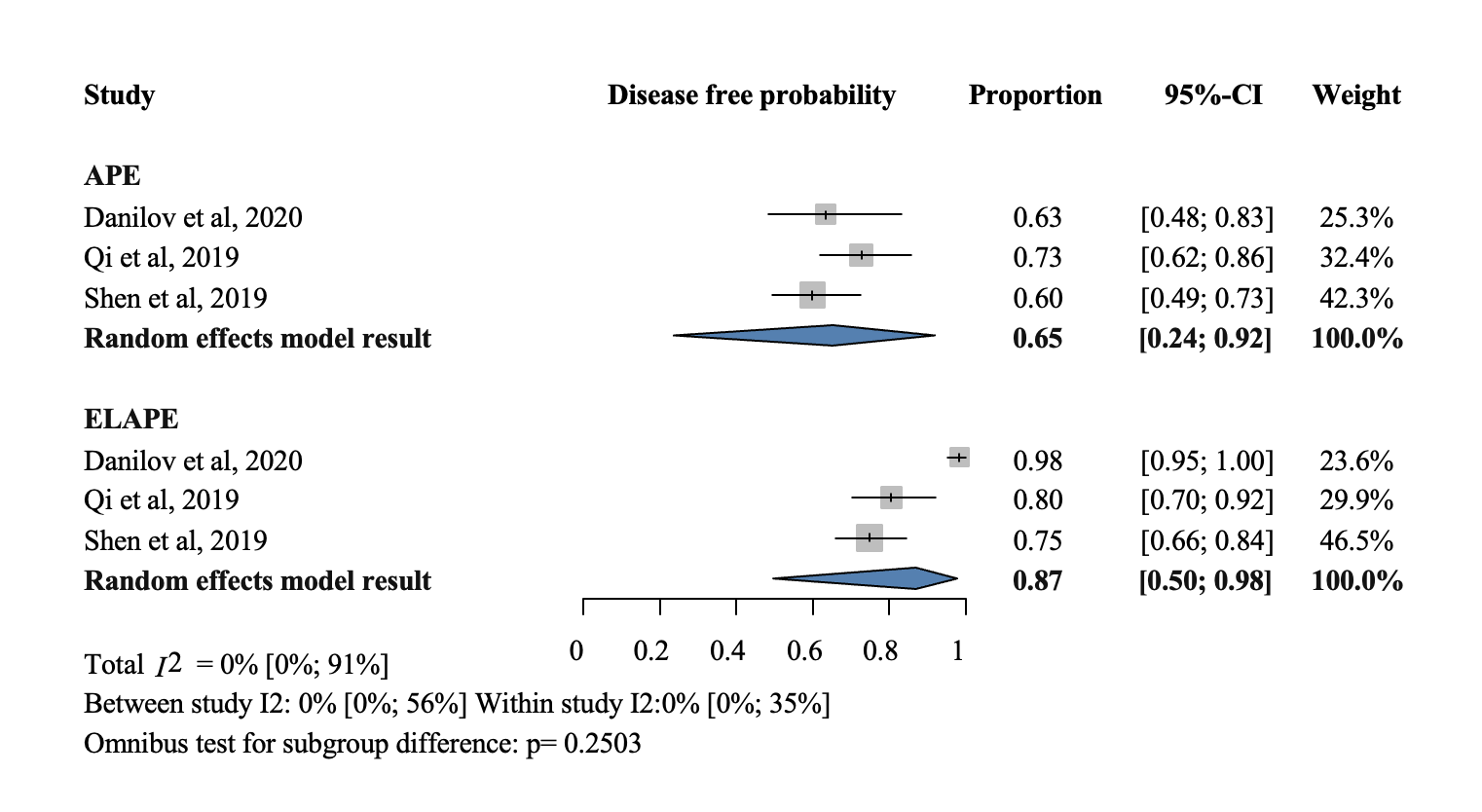


**Figure S13:** Kaplan-Meier curves for disease-free survival, showing individual study-specific survival probabilities for extralevator abdominoperineal excision and standard abdominoperineal excision.


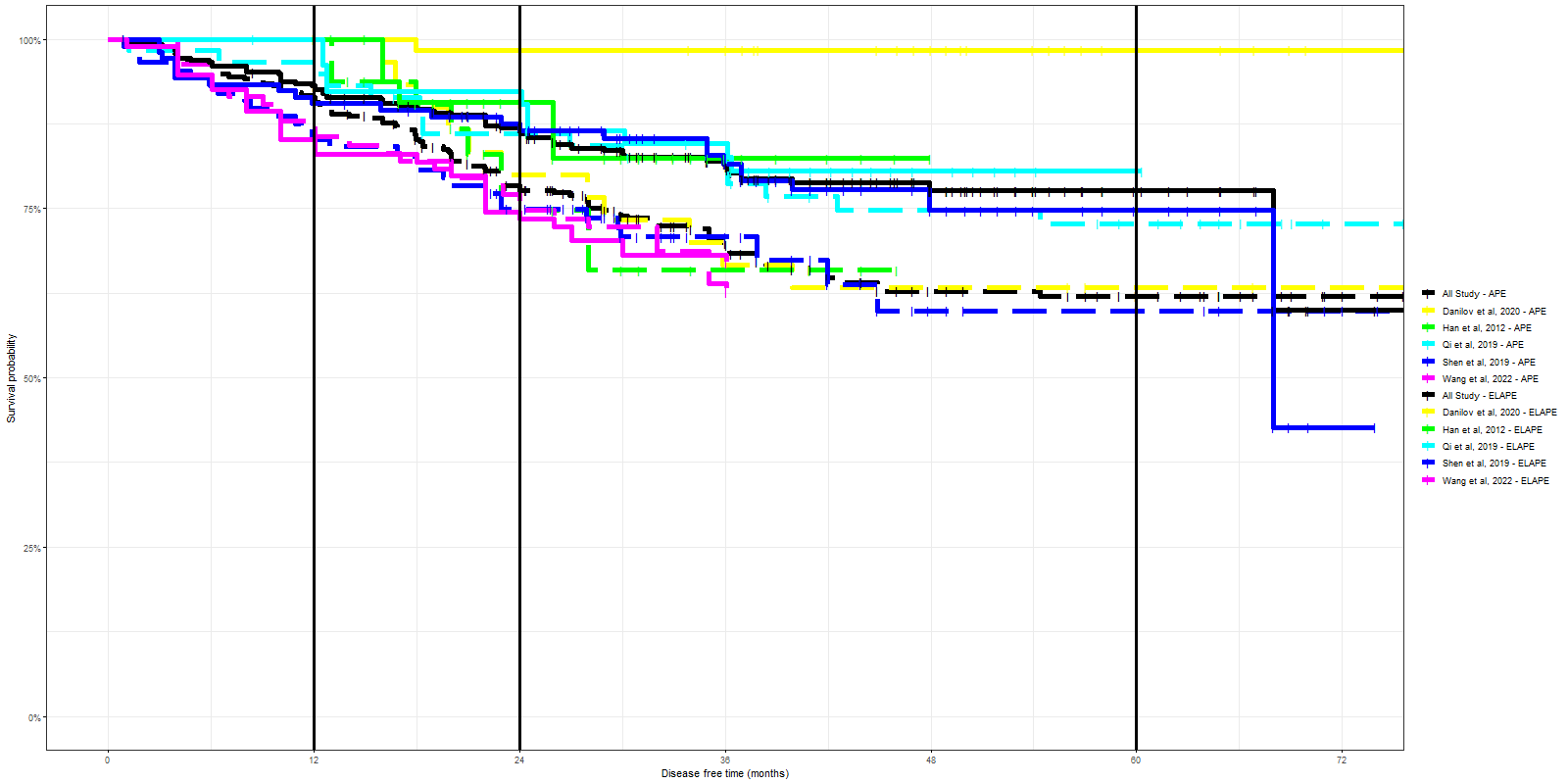


**Figure S14:** Forest plot representing exploratory pooled disease-free survival effects for ELAPE vs APE after integrating the two registry studies with the previously analyzed data

**
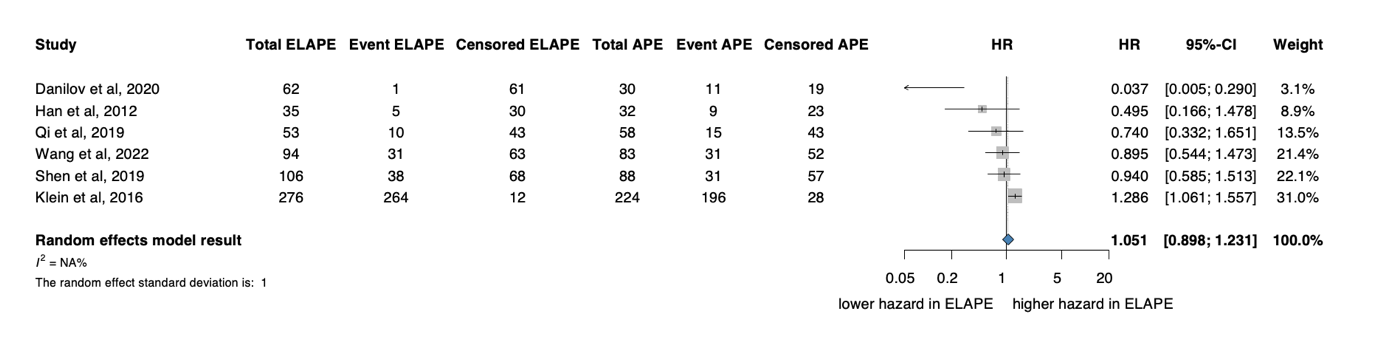
**

**Figure S15:** Forest plots representing local recurrence free survival following ELAPE vs. APE using “classical” analysis.


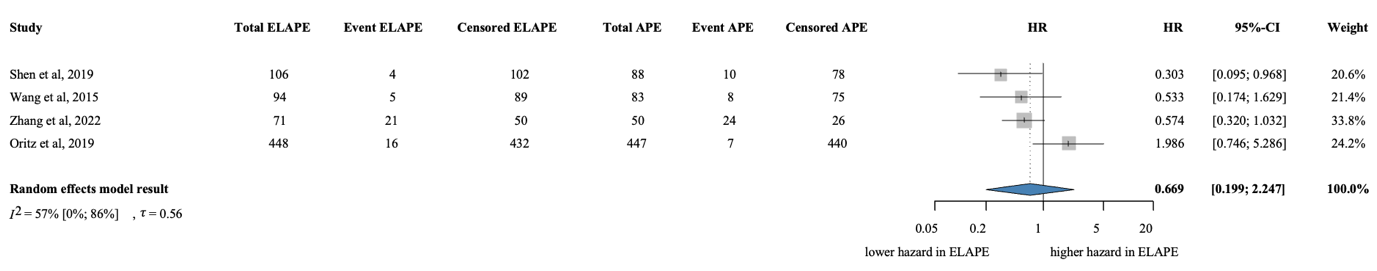


**Figure S16:** Estimated recurrence-free survival probability at 12 months following extralevator abdominoperineal excision vs. standard abdominoperineal excision
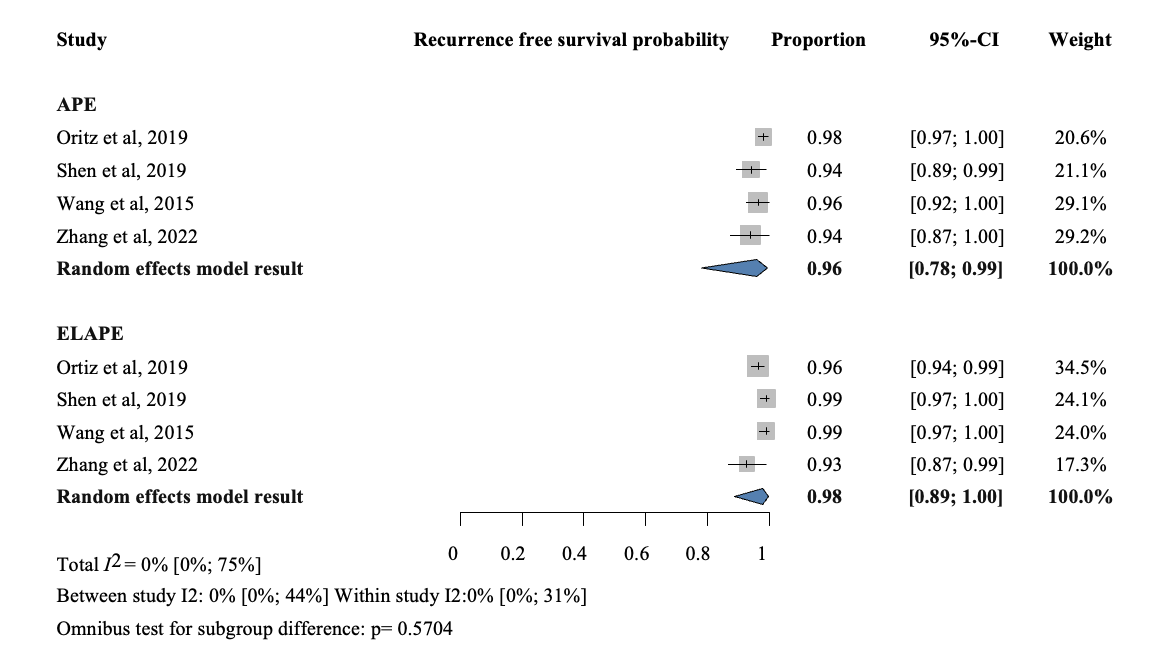


**Figure S17:** Estimated recurrence-free survival probability at 12 months following extralevator abdominoperineal excision vs. standard abdominoperineal excision


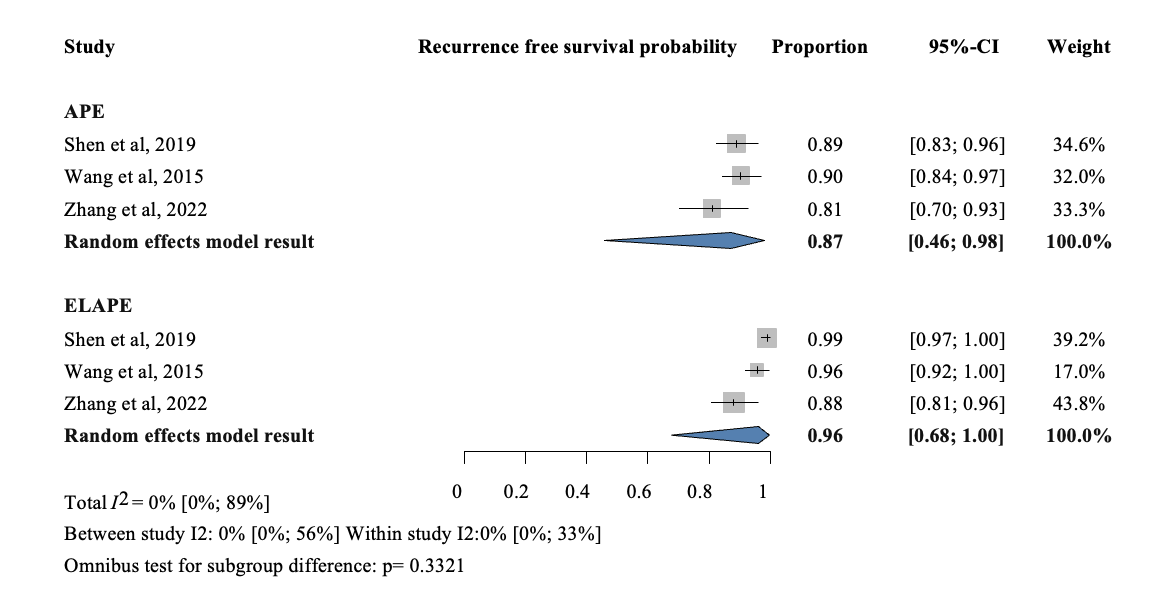


**Figure S18:** Estimated recurrence-free survival probability at 60 months following extralevator abdominoperineal excision vs. standard abdominoperineal excision


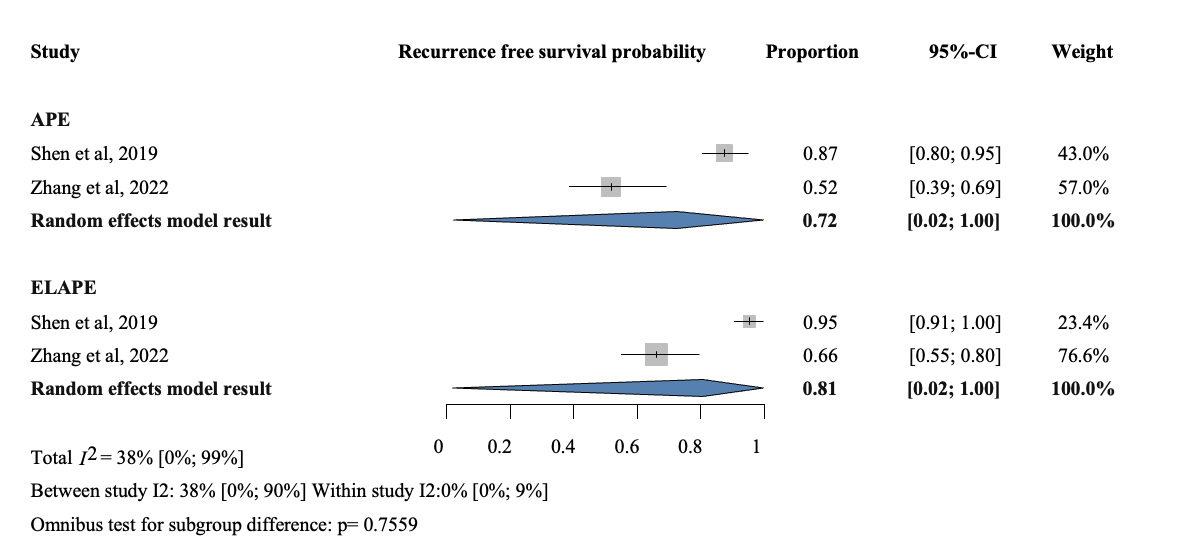


**Figure S19:** Kaplan-Meier curves for local-recurrence-free survival, showing individual study-specific survival probabilities for extralevator abdominoperineal excision and standard abdominoperineal excision.


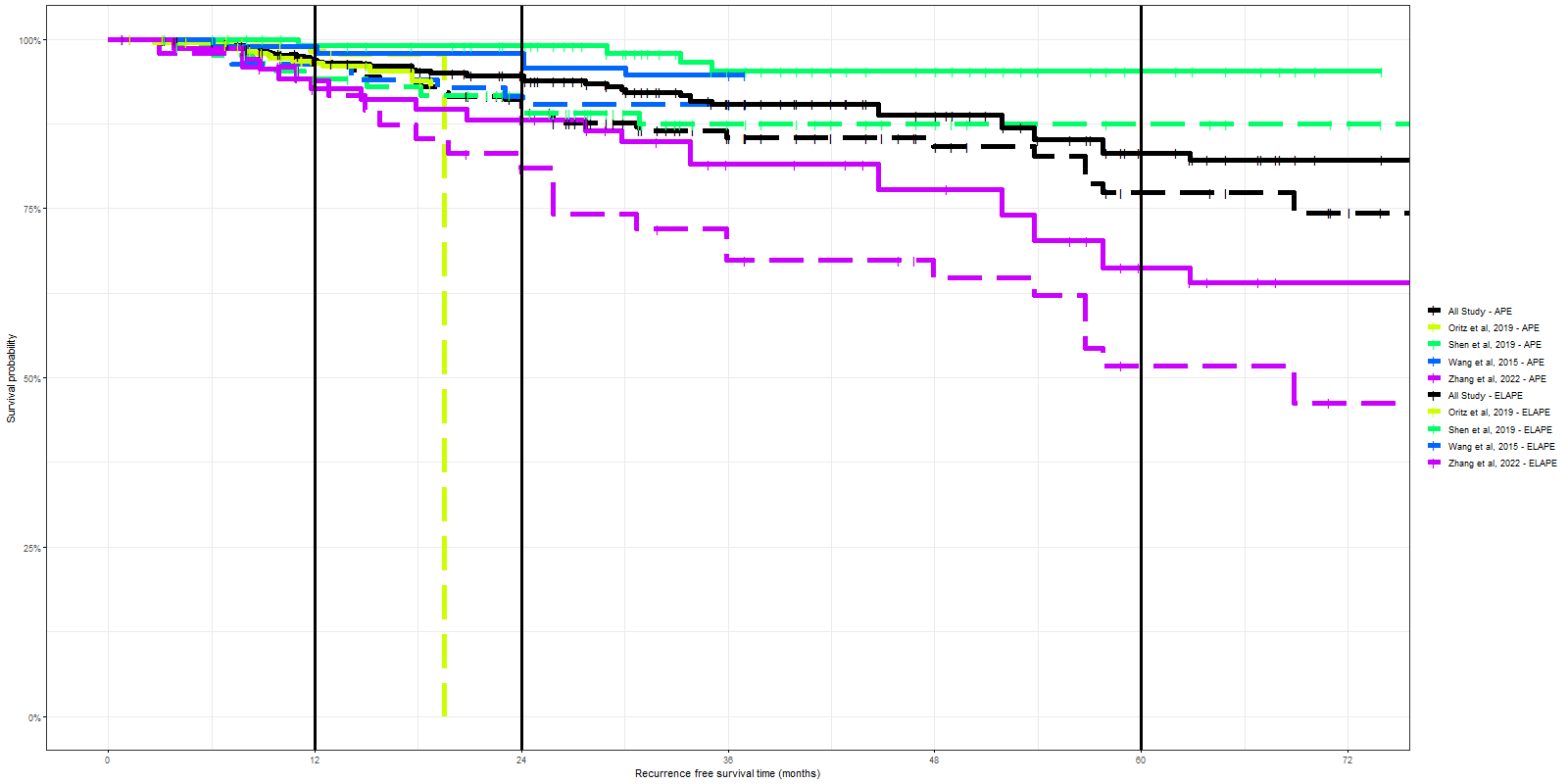


**Figure S20:** Forest plot comparing the risk of intraoperative perforation following extralevator abdominoperineal excision to standard abdominoperineal excision **
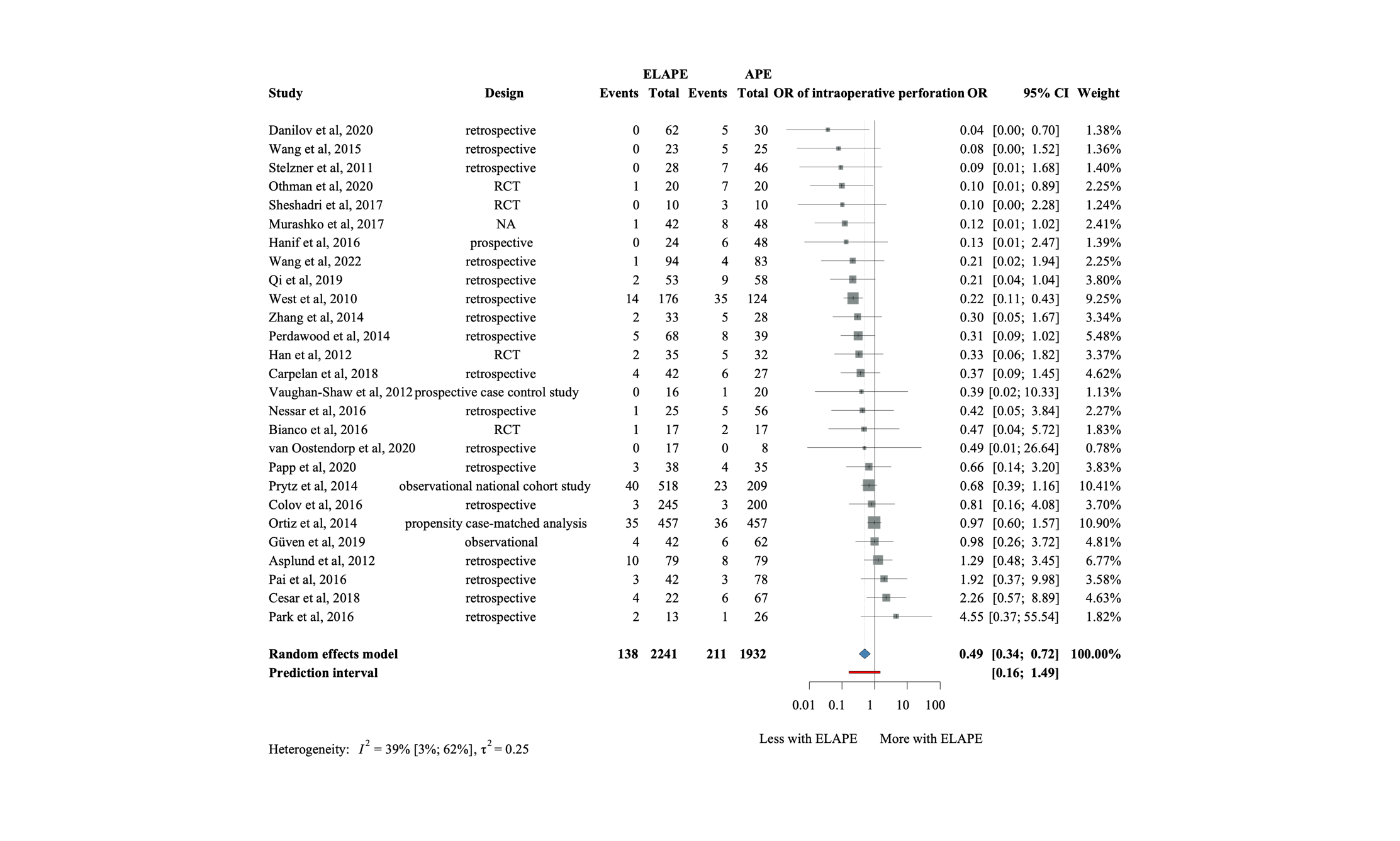
**

**Figure S21:** Forest plot comparing the risk of intraoperative perforation following extralevator abdominoperineal excision to standard abdominoperineal excision – subgroup of RCT’s

**
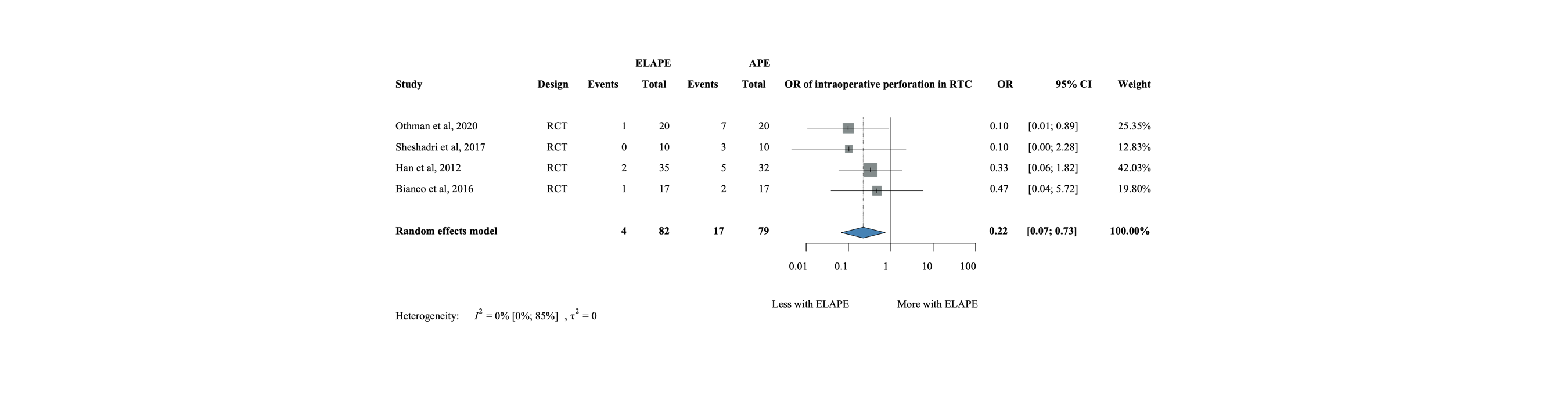
**

**Figure S22:** Forest plot comparing the risk of positive CRM following extralevator abdominoperineal excision to standard abdominoperineal excision

**
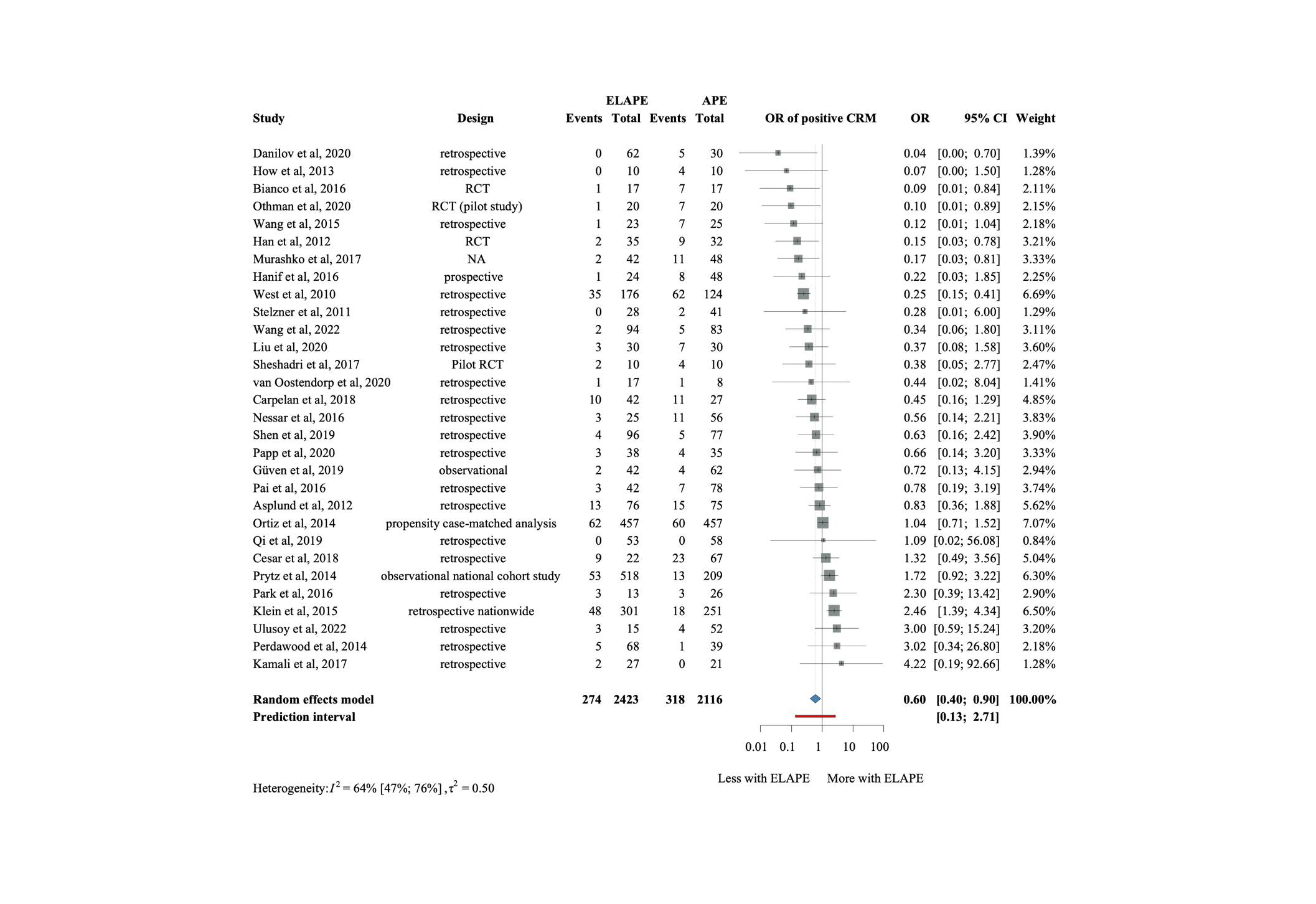
**

**Figure S23:** Forest plot comparing the risk of positive CRM following extralevator abdominoperineal excision to standard abdominoperineal excision - subgroup of RCT’s


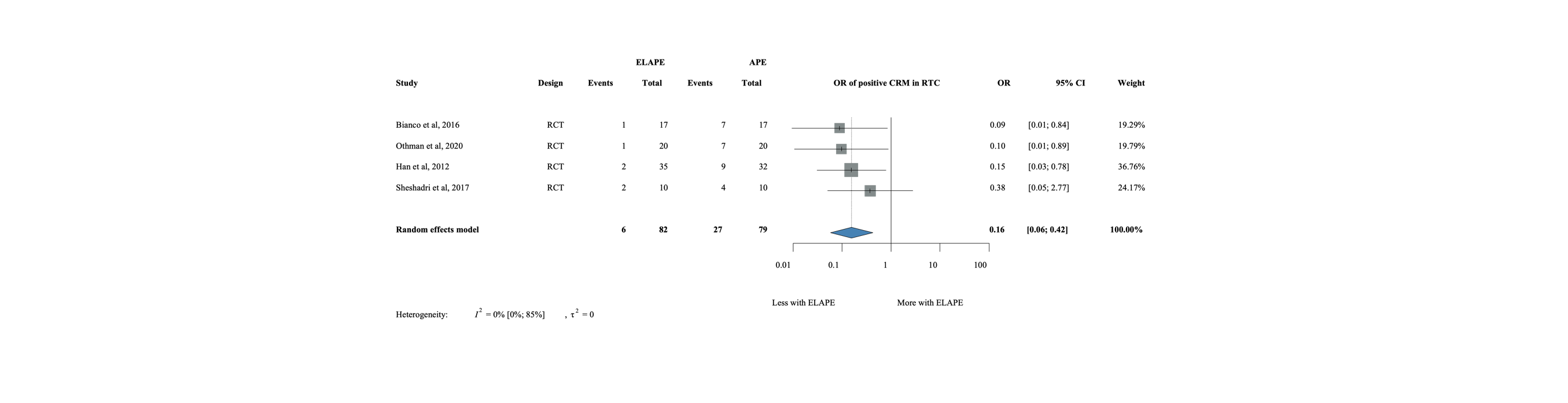


**Figure S24:** Forest plot comparing the blood loss during extralevator abdominoperineal excision to standard abdominoperineal excision


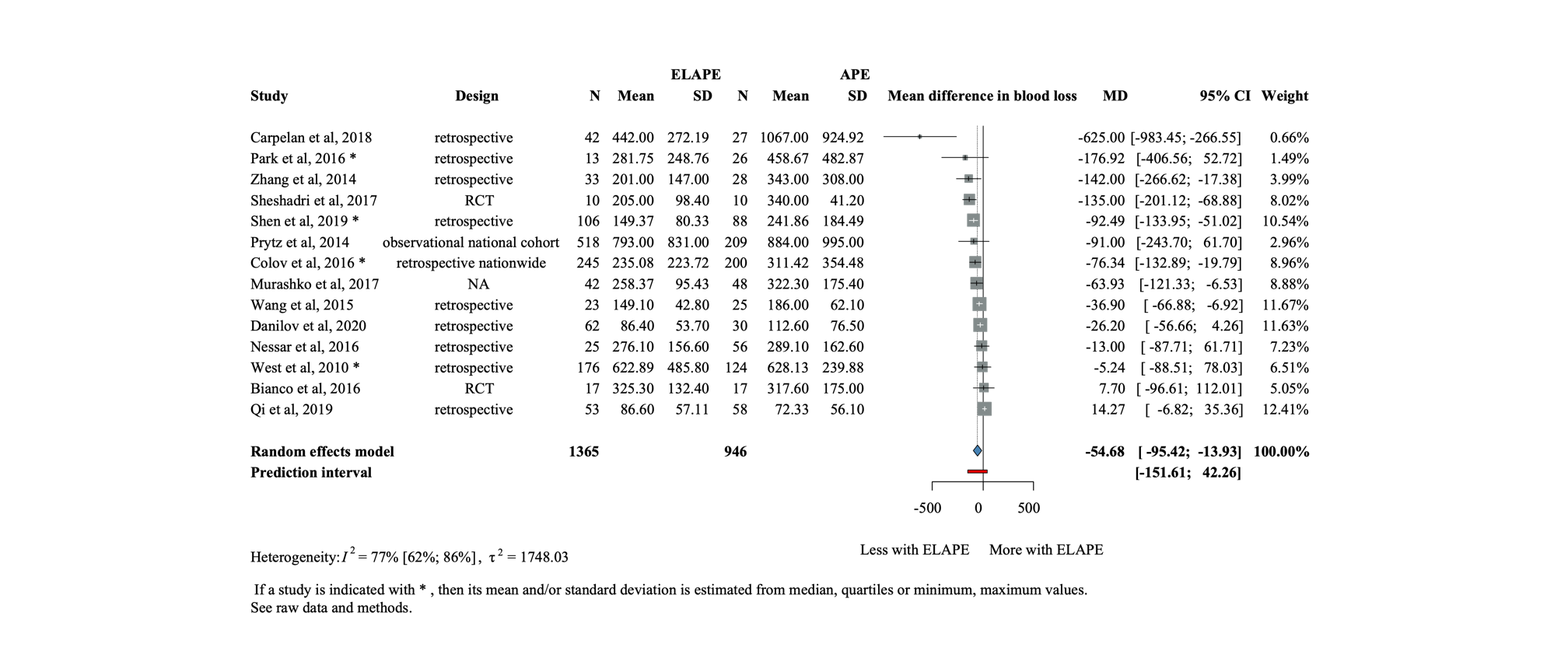


**Figure S25:** Forest plot comparing the operative time of extralevator abdominoperineal excision to standard abdominoperineal excision


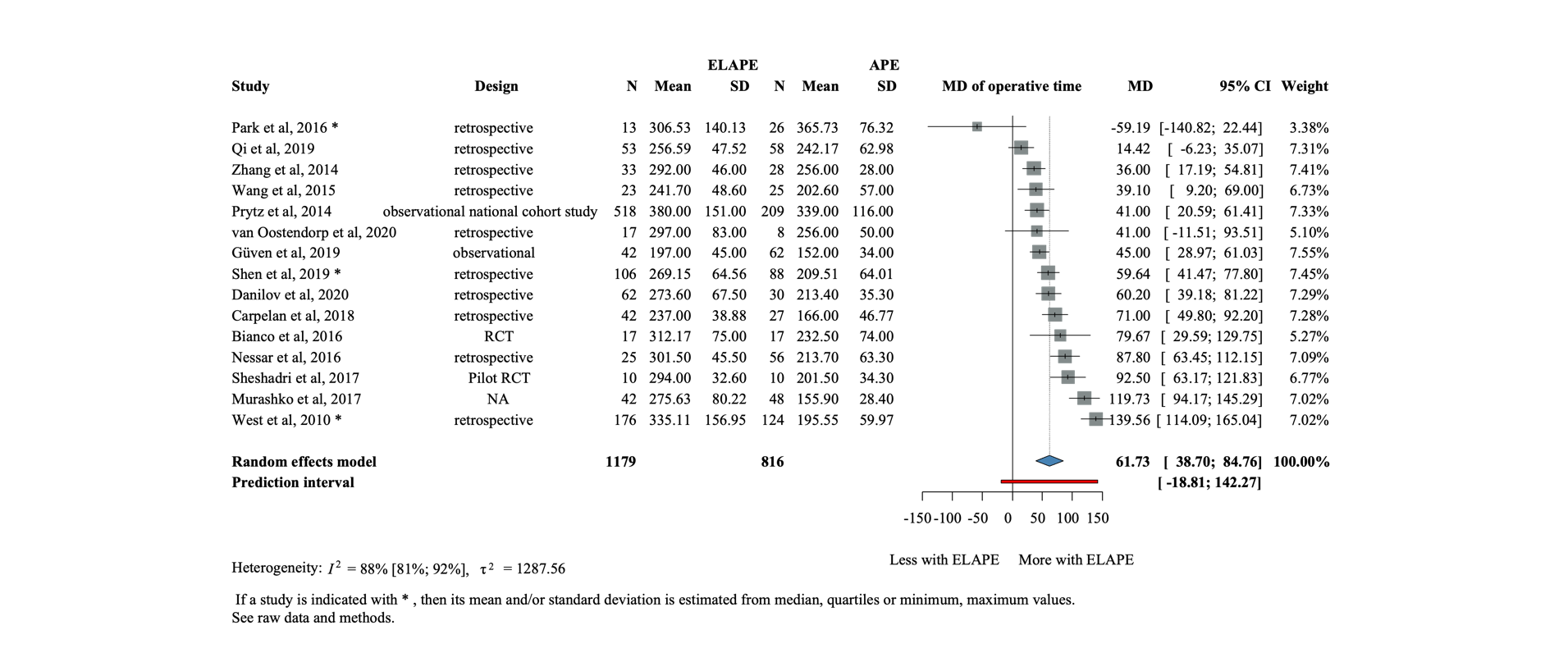


**Figure S26:** Forest plot comparing the mortality following extralevator abdominoperineal excision to standard abdominoperineal excision


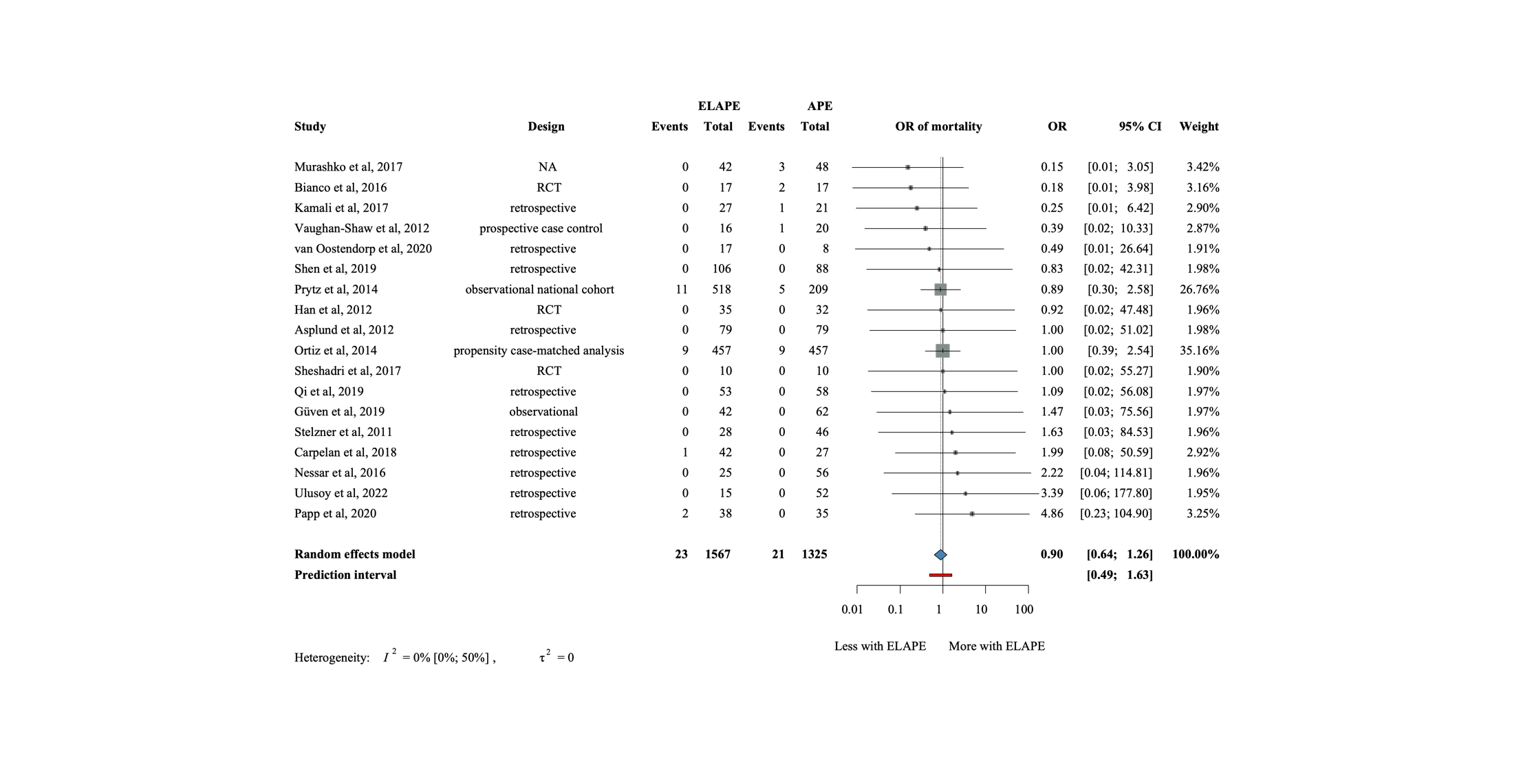


**Figure S27:** Forest plot comparing the mortality following extralevator abdominoperineal excision to standard abdominoperineal excision - subgroup of RCT’s


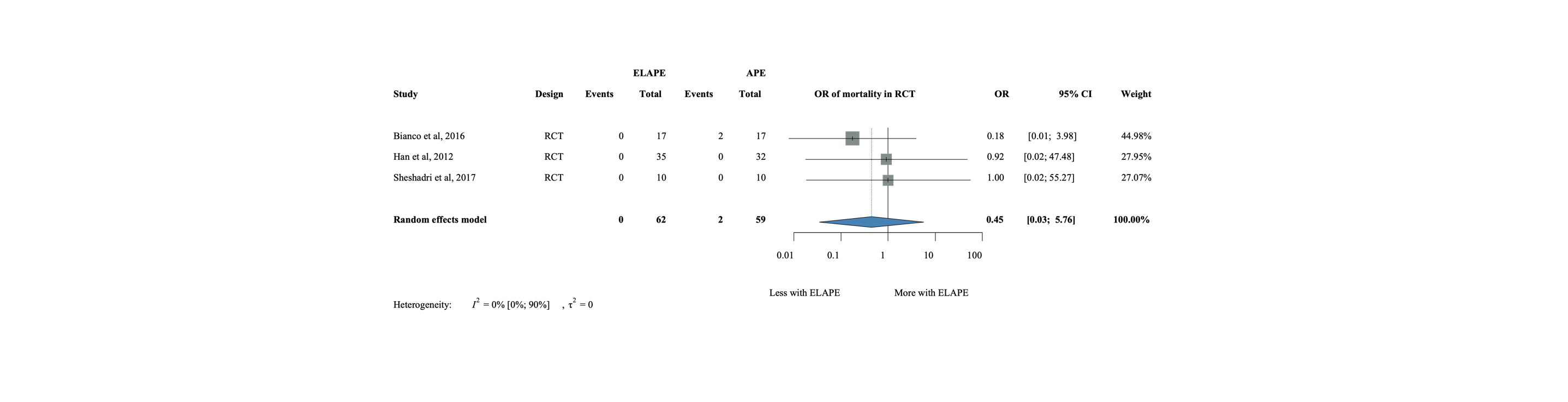


**Figure S28:** Forest plot comparing the overall complications following extralevator abdominoperineal excision to standard abdominoperineal excision


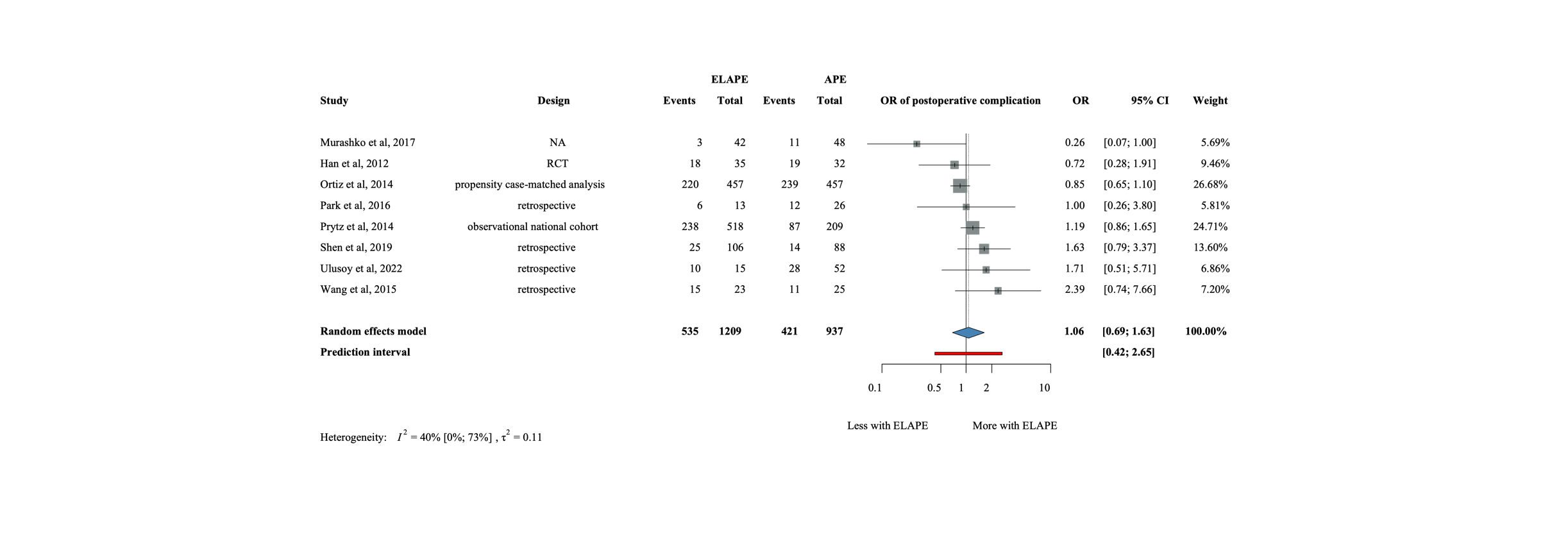


**Figure S29:** Forest plot comparing the reoperation rates following extralevator abdominoperineal excision to standard abdominoperineal excision


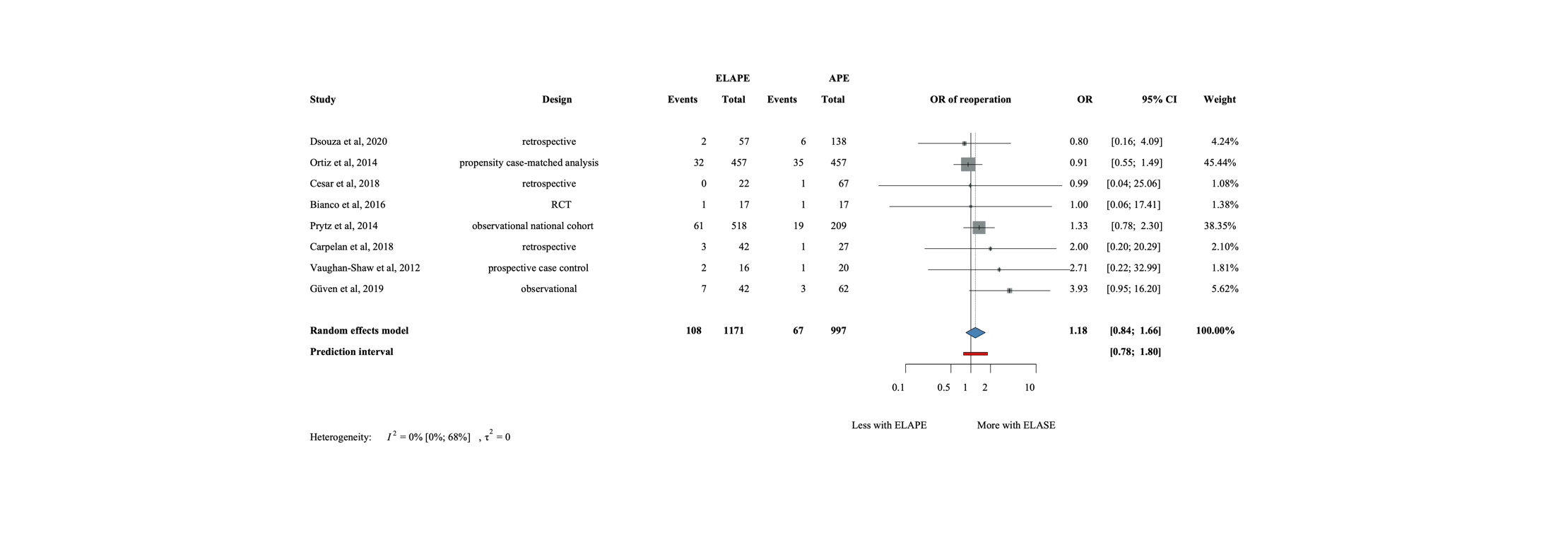


**Figure S30:** Forest plot comparing the hospital stay following extralevator abdominoperineal excision to standard abdominoperineal excision


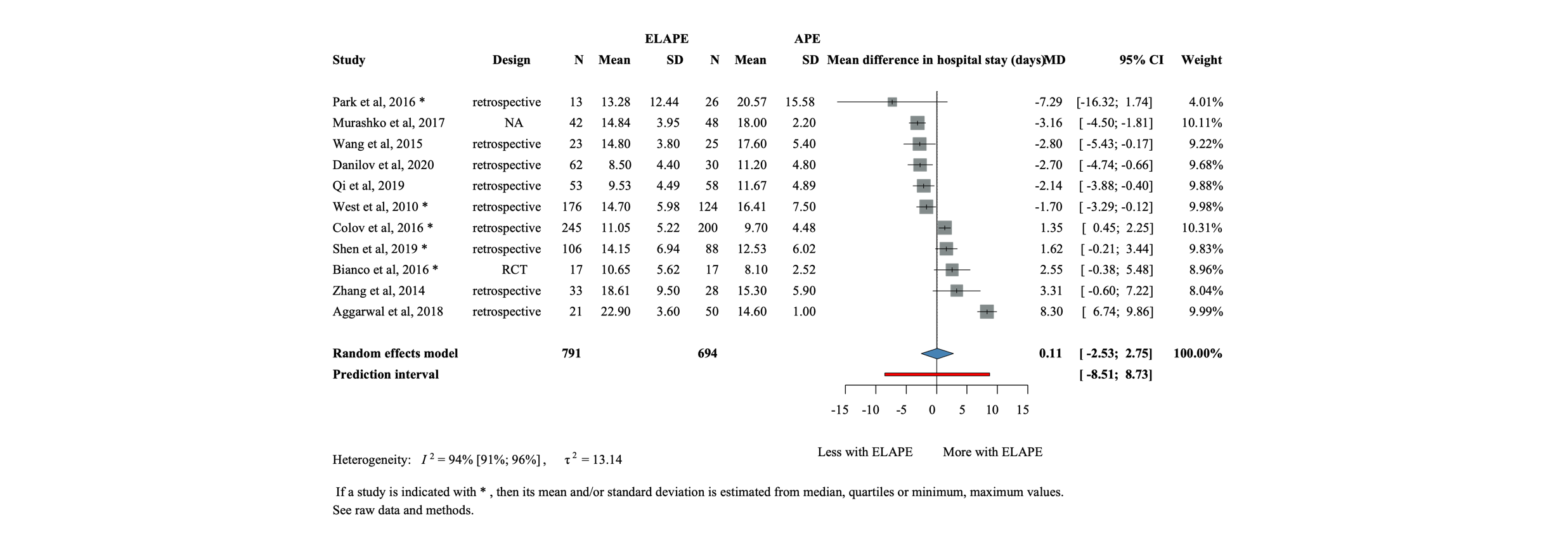


**Figure S31:** Forest plot comparing the perineal wound complication rates following extralevator abdominoperineal excision to standard abdominoperineal excision


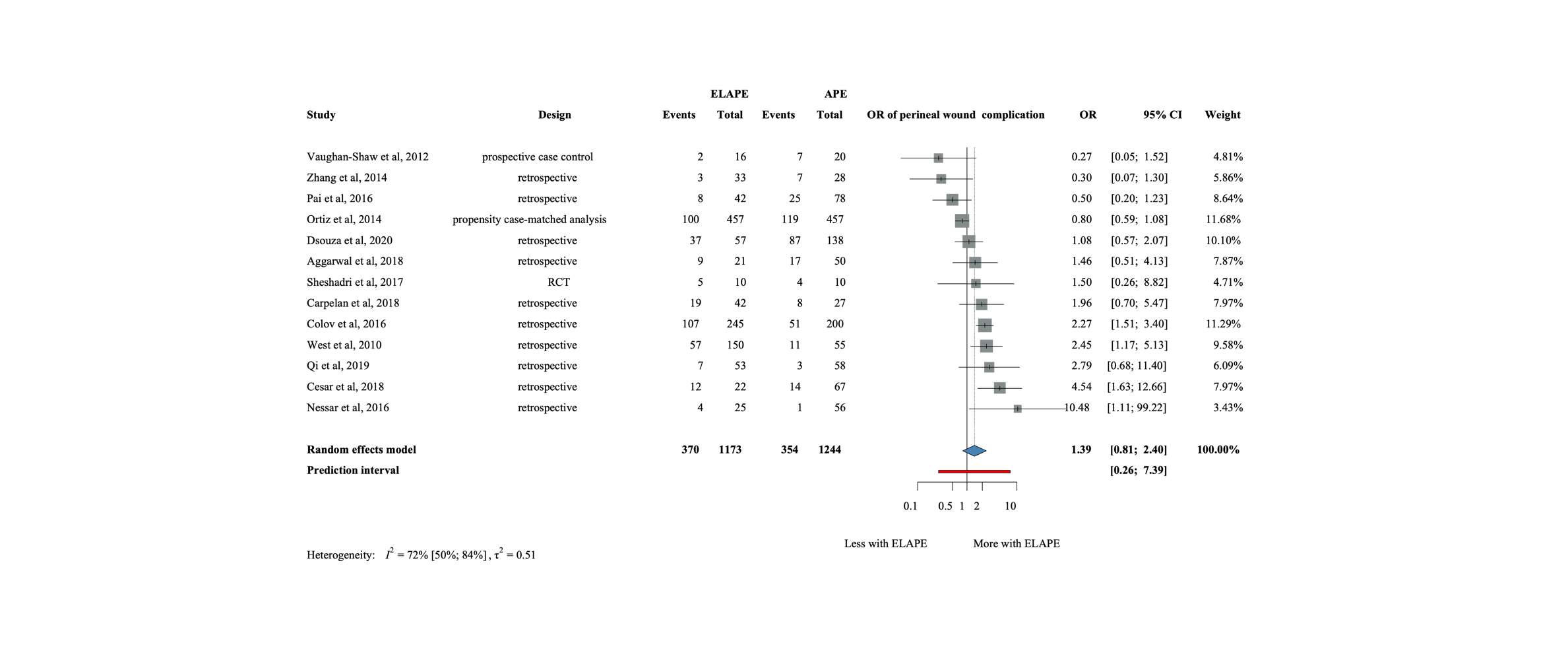


**Figure S32:** Forest plot comparing the perineal wound infection rates following extralevator abdominoperineal excision to standard abdominoperineal excision


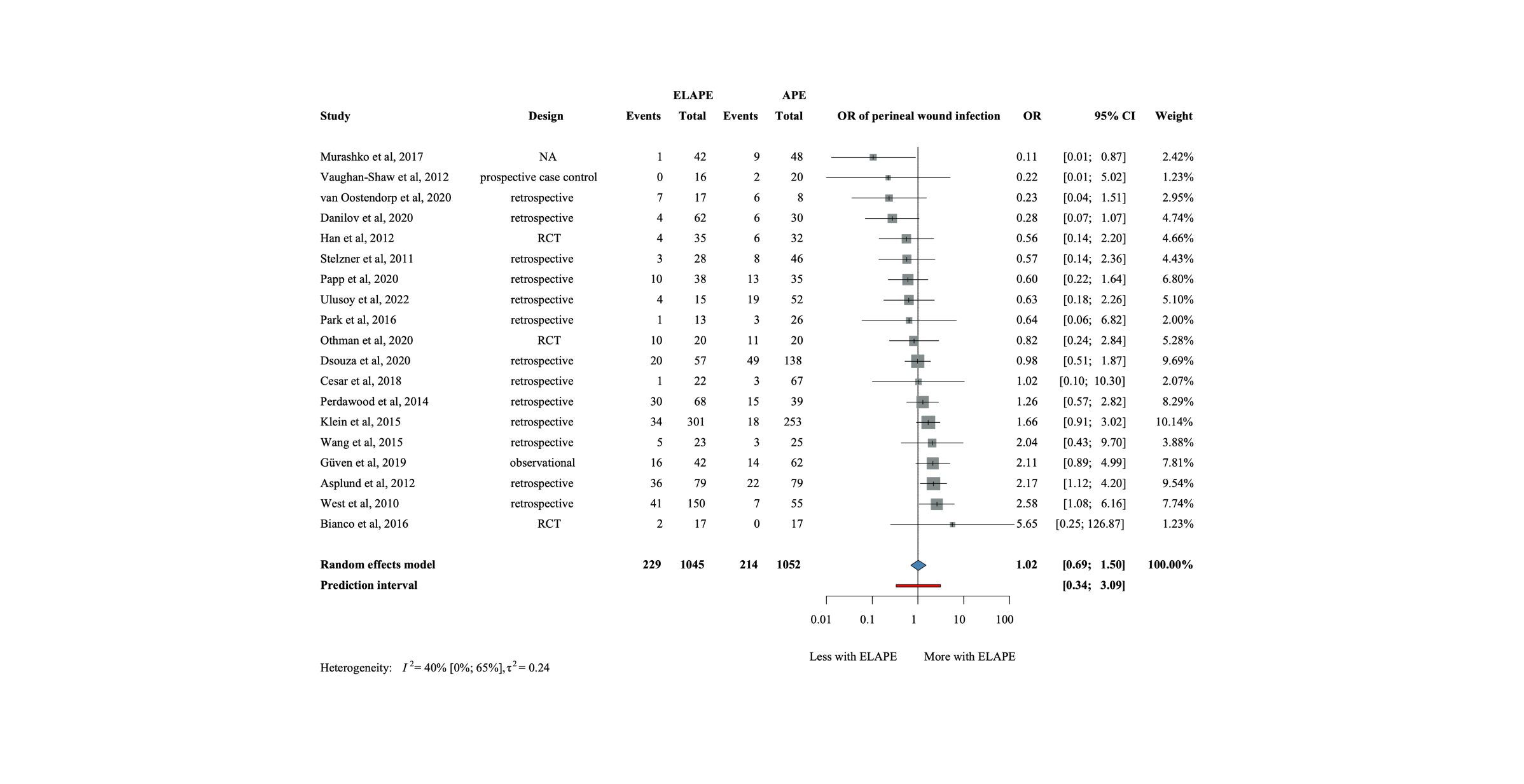


**Figure S33:** Forest plot comparing the perineal wound infection rates following extralevator abdominoperineal excision to standard abdominoperineal excision - subgroup of RCT’s


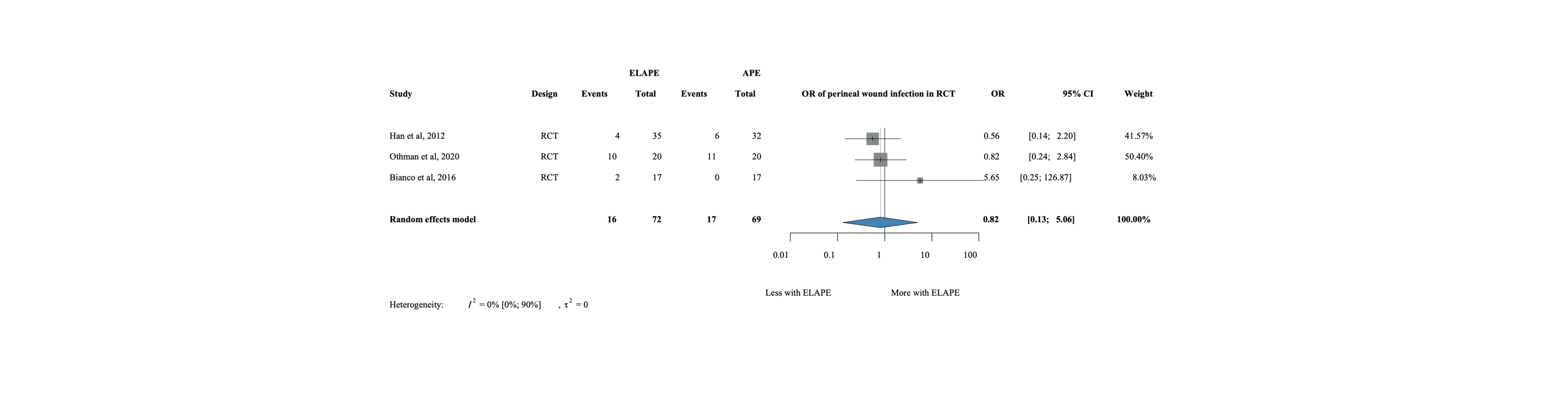


**Figure S34:** Forest plot comparing the perineal wound dehiscence rates following extralevator abdominoperineal excision to standard abdominoperineal excision


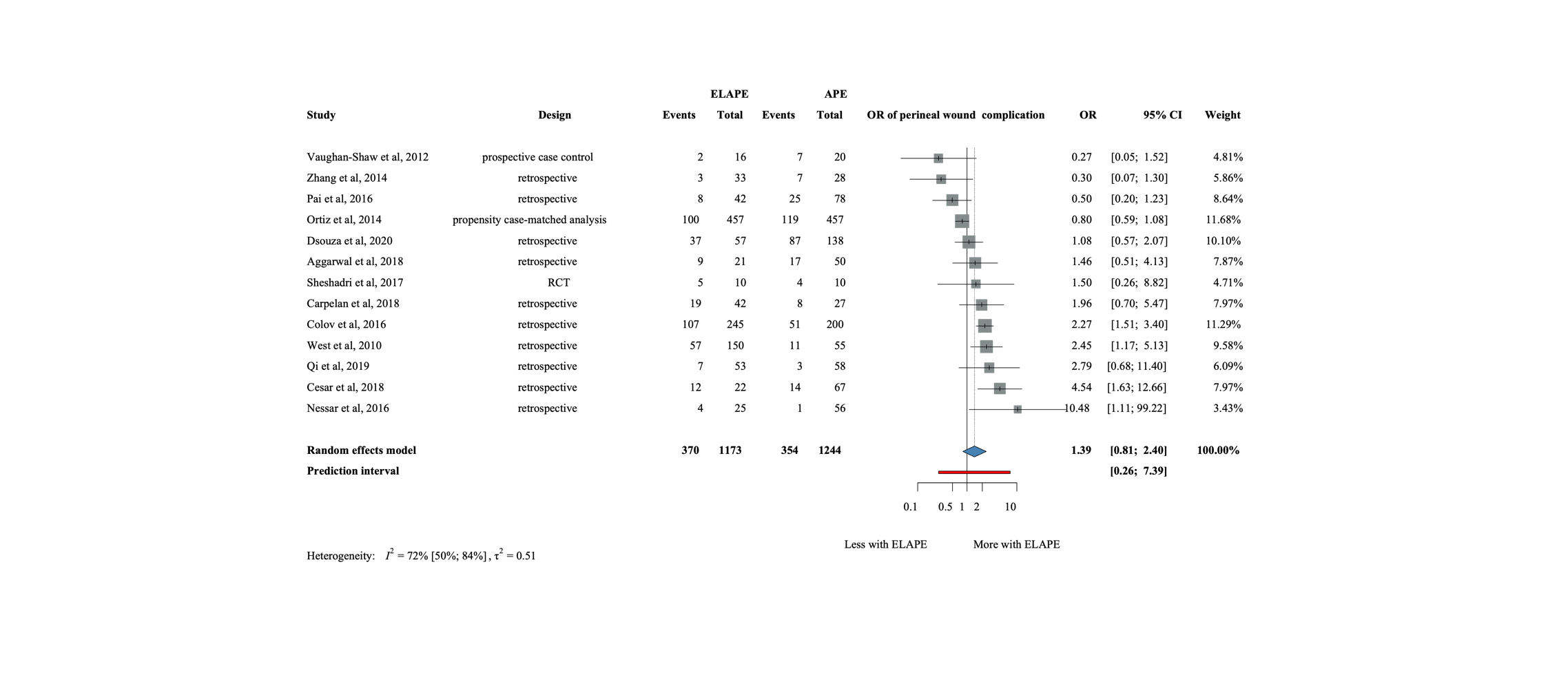


**Figure S35:** Forest plot comparing the perineal hernia rates following extralevator abdominoperineal excision to standard abdominoperineal excision


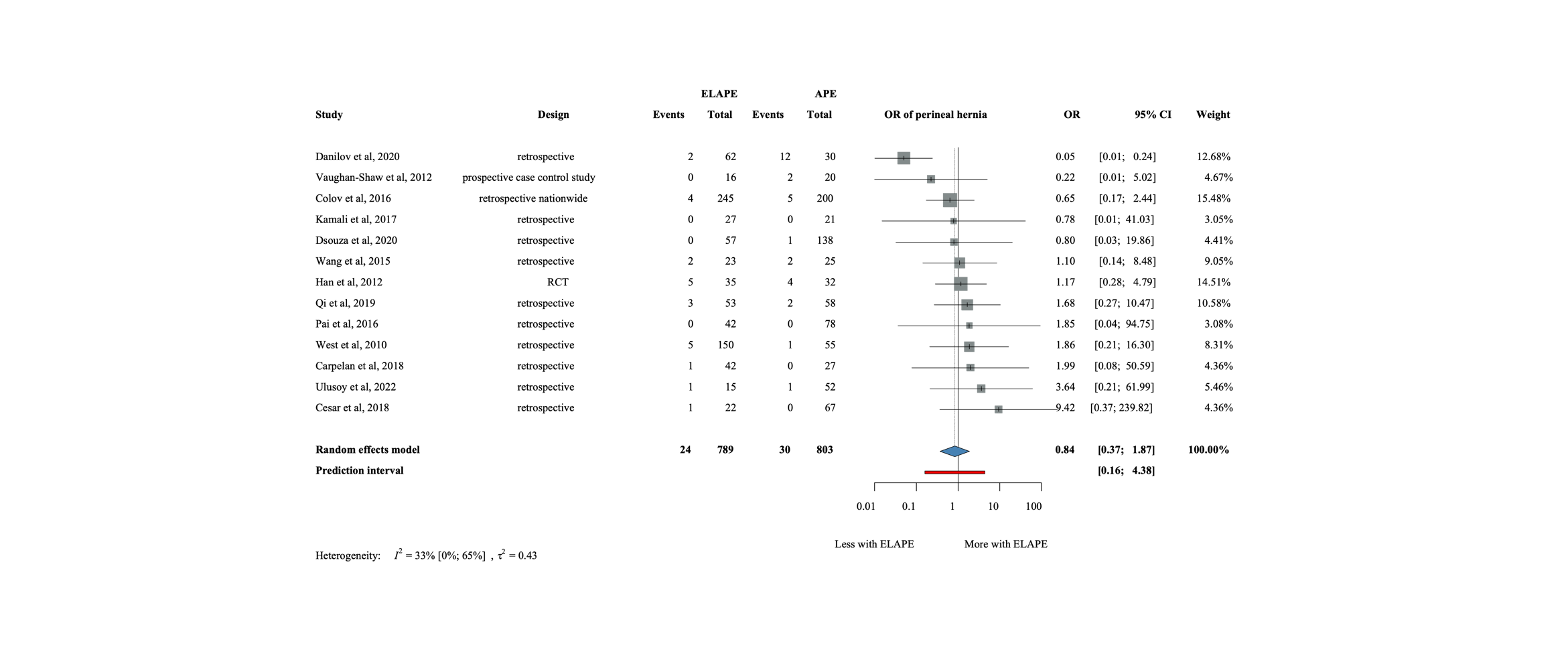


**Figure S36:** Forest plot comparing the existence of perineal pain following extralevator abdominoperineal excision to standard abdominoperineal excision


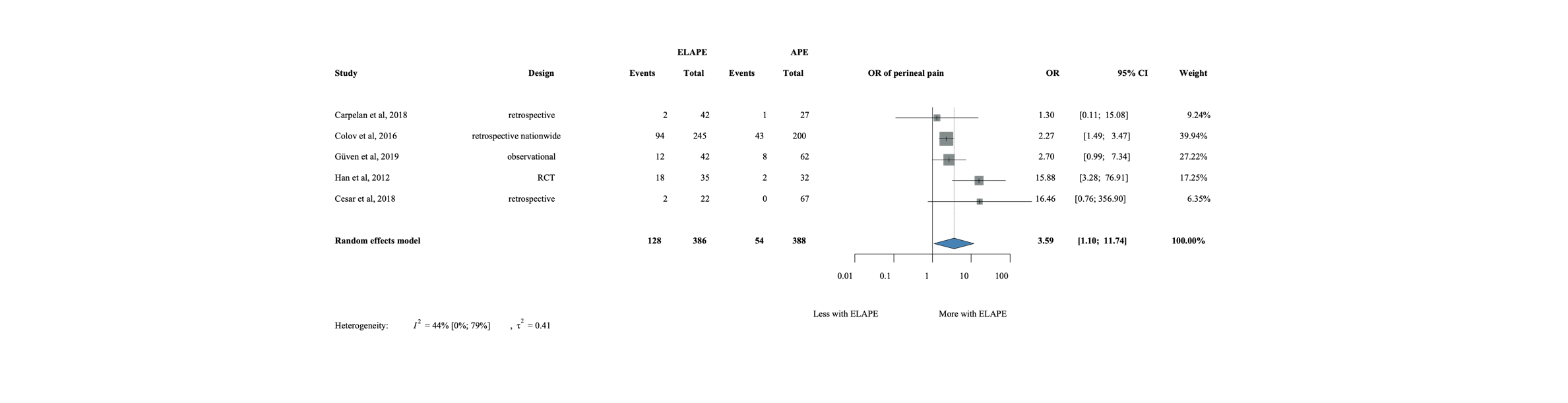


**Table S4**: Risk of bias assessment for the non-randomized studies by ROBINS tool

| **Study** | **Pre-intervention** | | **At-intervention** | **Post-intervention** | | | | **Overall risk of bias** |
| --- | --- | --- | --- | --- | --- | --- | --- | --- |
|  | **Bias due to confounding** | **Selection of participants** | **Classification of interventions** | **Deviation from intended interventions** | **Missing data** | **Measurement of Outcomes** | **Selection of reported results** |  |
|  | **D1** | **D2** | **D3** | **D4** | **D5** | **D6** | **D7** |  |
| Aggarwal et al, 2018 | Moderate | Low | **Low** | Low | Low | Low | Low | Moderate |
| Asplund et al, 2012 | Moderate | Low | **Low** | Low | Moderate | Moderate | Low | Moderate |
| Carpelean et al, 2018 | Moderate | Low | **Low** | Low | Low | Moderate | Low | Moderate |
| Cesar et al, 2018 | Serious | Moderate | **Low** | Low | Moderate | Moderate | Low | Serious |
| Colov et al, 2016 | Moderate | Low | **Low** | Low | Moderate | Moderate | Moderate | Moderate |
| Danilov et al, 2020 | Moderate | Low | **Low** | Low | Low | Low | Low | Moderate |
| Dsouza et al, 2020 | Moderate | Low | **Low** | Low | Moderate | Moderate | Moderate | Moderate |
| Güven et al, 2019 | Moderate | Low | **Low** | Low | Low | Low | Low | Moderate |
| Hanif et al, 2016 | Serious | Moderate | **Low** | Low | Low | Moderate | Low | Serious |
| How et al, 2013 | Moderate | Low | **Low** | Low | Low | Low | Low | Moderate |
| Kamali et al, 2017 | Moderate | Low | **Low** | Low | Moderate | Moderate | Low | Moderate |
| Klein et al, 2015 | Moderate | Low | **Low** | Low | Moderate | Moderate | Low | Moderate |
| Klein et al, 2016 | Moderate | Low | **Low** | Low | Moderate | Moderate | Low | Moderate |
| Liu et al, 2020 | Moderate | Low | **Low** | Low | Moderate | Low | Low | Moderate |
| Murashko et al, 2017 | Moderate | Moderate | **Low** | Low | Low | Moderate | Low | Moderate |
| Nessar et al, 2016 | Serious | Moderate | **Low** | Low | Low | Moderate | Low | Serious |
| Ortiz et al, 2014 | Low | Low | **Low** | Low | Low | Low | Low | Low |
| Pai et al, 2016 | Moderate | Low | **Low** | Low | Low | Moderate | Low | Moderate |
| Papp et al, 2020 | Moderate | Low | **Low** | Low | Moderate | Moderate | Low | Moderate |
| Park et al, 2016 | Moderate | Low | **Low** | Low | Moderate | Moderate | Low | Moderate |
| Perdawood et al, 2014 | Moderate | Low | **Low** | Low | Moderate | Moderate | Low | Moderate |
| Prytz et al, 2014 | Moderate | Low | **Low** | Low | Moderate | Low | Low | Moderate |
| Prytz et al, 2016 | Moderate | Low | **Low** | Low | Low | Low | Low | Moderate |
| Qi et al, 2019 | Moderate | Low | **Low** | Low | Moderate | Low | Low | Moderate |
| Shen et al, 2019 | Moderate | Low | **Low** | Low | Moderate | Moderate | Low | Moderate |
| Stelzner et al, 2011 | Moderate | Low | **Low** | Low | Low | Low | Low | Moderate |
| Stelzner et al, 2016 | Moderate | Low | **Low** | Low | Moderate | Moderate | Low | Moderate |
| Ulusoy et al, 2022 | Moderate | Low | **Low** | Low | Moderate | Moderate | Low | Moderate |
| van Oostendorp et al, 2020 | Moderate | Low | **Low** | Low | Moderate | Moderate | Low | Moderate |
| Vaughan-Shaw et al, 2012 | Moderate | Low | **Low** | Low | Low | Low | Low | Moderate |
| Wang et al, 2015 | Moderate | Low | **Low** | Low | Low | Moderate | Low | Moderate |
| Wang et al, 2022 | Moderate | Low | **Low** | Low | Moderate | Moderate | Low | Moderate |
| West et al, 2010 | Moderate | Low | **Low** | Low | Moderate | Moderate | Low | Moderate |
| Wu et al, 2013 | Moderate | Low | **Low** | Low | Moderate | Moderate | Low | Moderate |
| Xiao et al, 2014 | Moderate | Low | **Low** | Low | Moderate | Moderate | Low | Moderate |
| Zhang et al, 2014 | Moderate | Low | **Low** | Low | Moderate | Moderate | Low | Moderate |
| Zhang et al, 2022 | Moderate | Low | **Low** | Low | Moderate | Moderate | Low | Moderate |

**Table S5:** Risk of bias assessment for the randomized studies by ROB-2 tool

| **Study** | **Randomization process** | **Deviations from intended interventions** | **Missing outcome data** | **Measurement of the outcome** | **Selection of the reported result** | **Overall Bias** |
| --- | --- | --- | --- | --- | --- | --- |
| Bianco et al, 2015 | Low | Low | Low | Low | Low | Low |
| Han et al, 2012 | Low | Low | Low | Low | Low | Low |
| Othman et al, 2020 | Low | Low | Low | Low | Low | Low |
| Seshadri et al, 2017 | Some concerns | Low | Low | Low | Low | Some concerns |

**Figure S37:** Funnel plot for overall survival following extralevator abdominoperineal excision compared to standard abdominoperineal excision


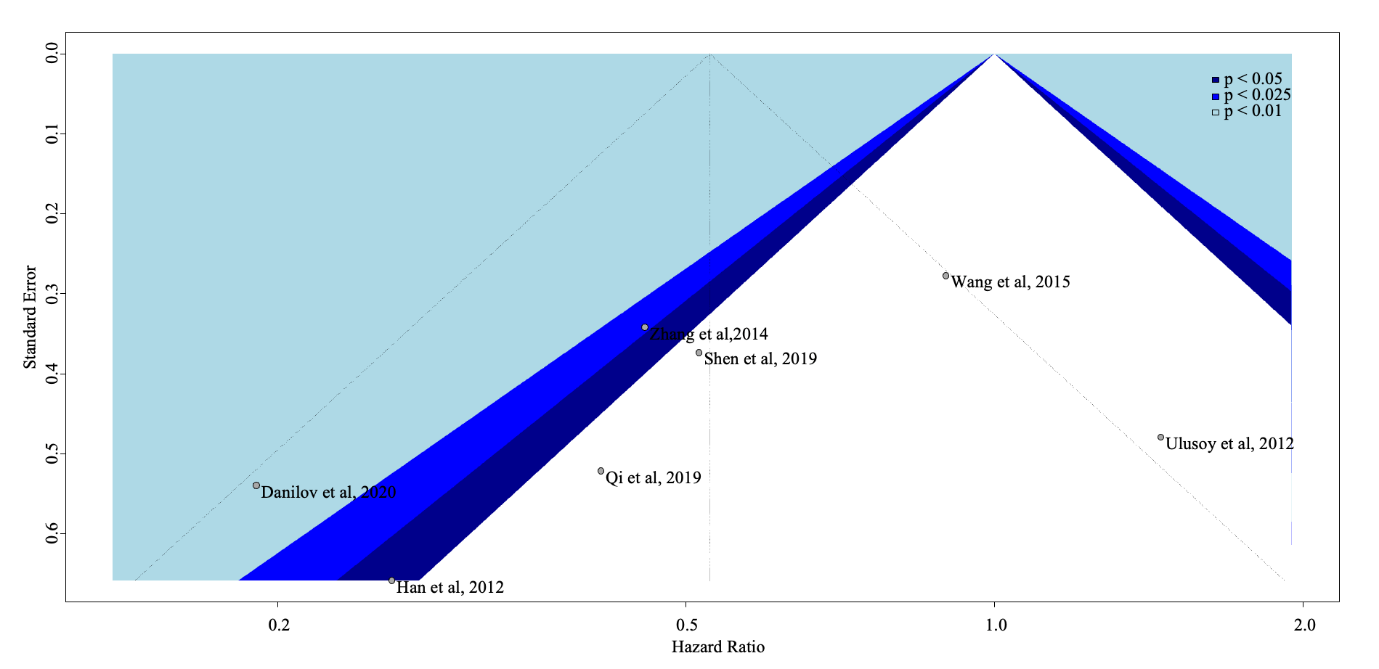


**Figure S38:** Funnel plot for disease-free survival following extralevator abdominoperineal excision compared to standard abdominoperineal excision


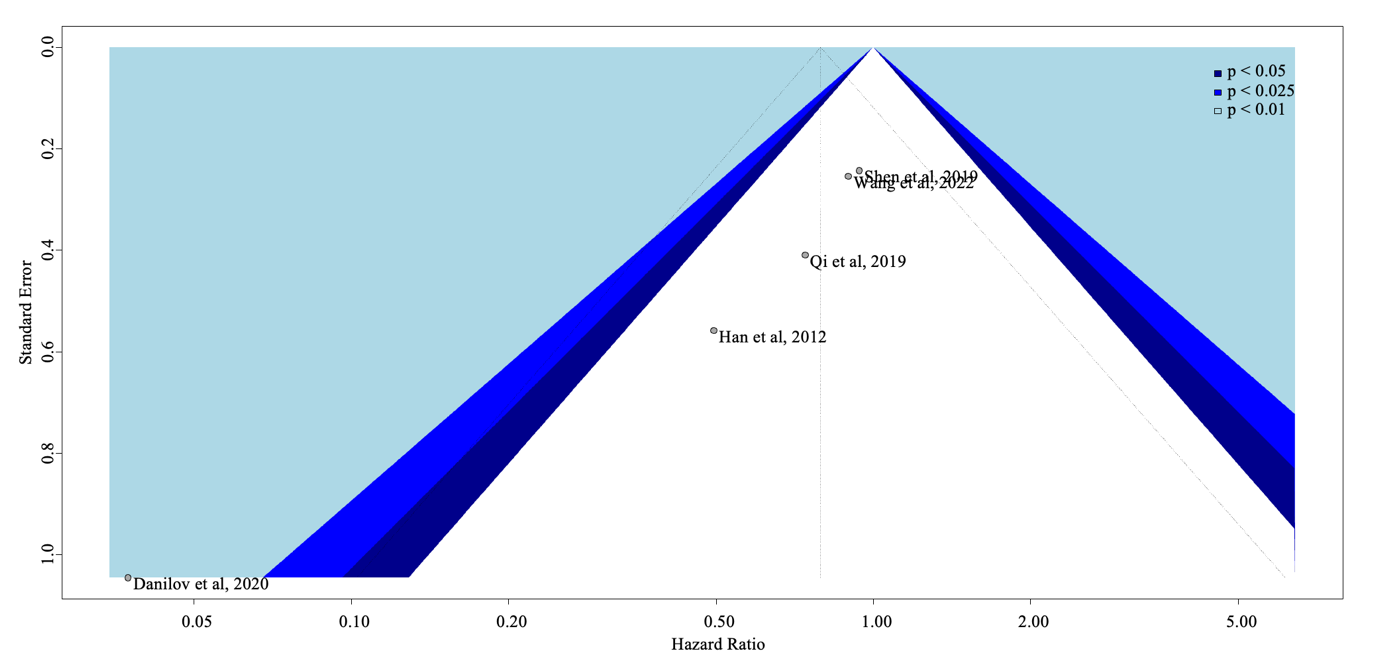


**Figure S39:** Funnel plot for local-recurrence-free survival following extralevator abdominoperineal excision compared to standard abdominoperineal excision

**
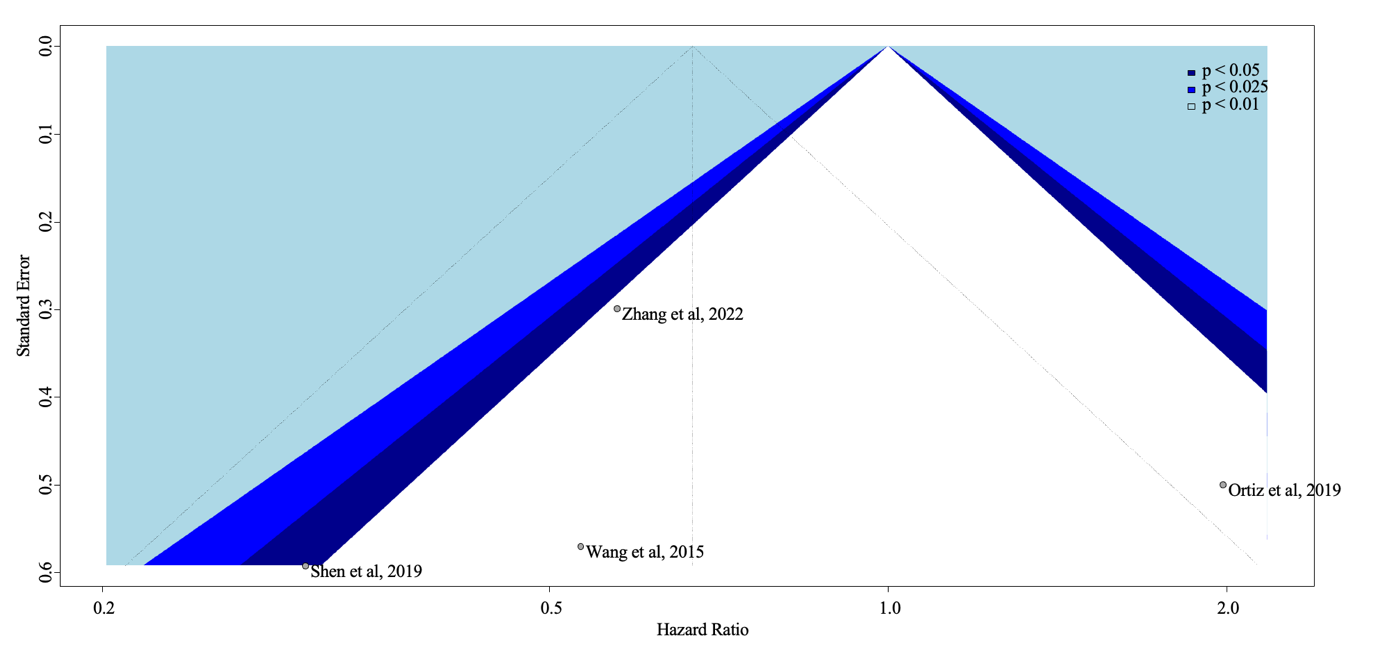
**

**Figure S40:** Funnel plot for IOP rates following extralevator abdominoperineal excision compared to standard abdominoperineal excision


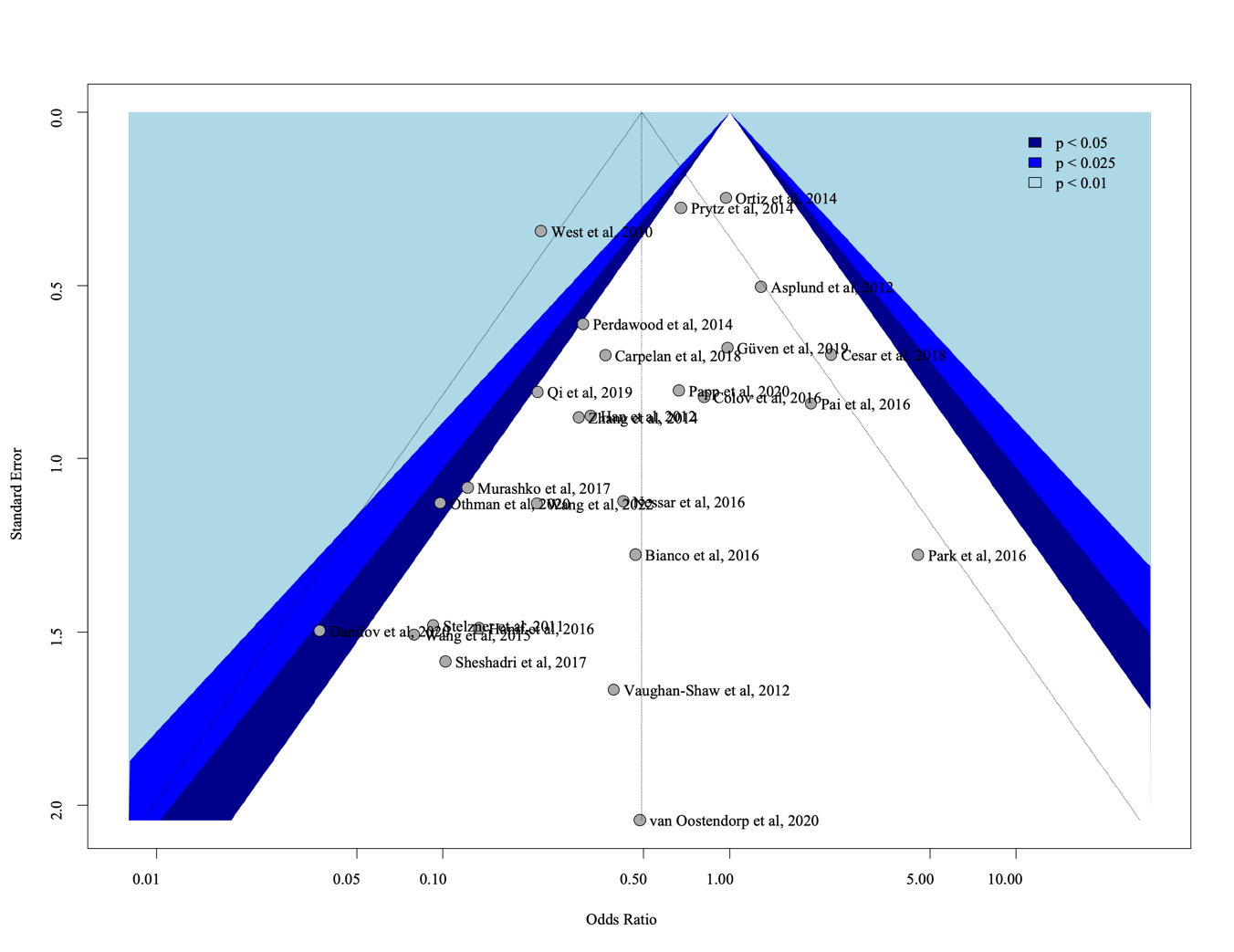


**Figure S41:** Funnel plot for IOP rates following extralevator abdominoperineal excision compared to standard abdominoperineal excision – subgroup of RCT’s


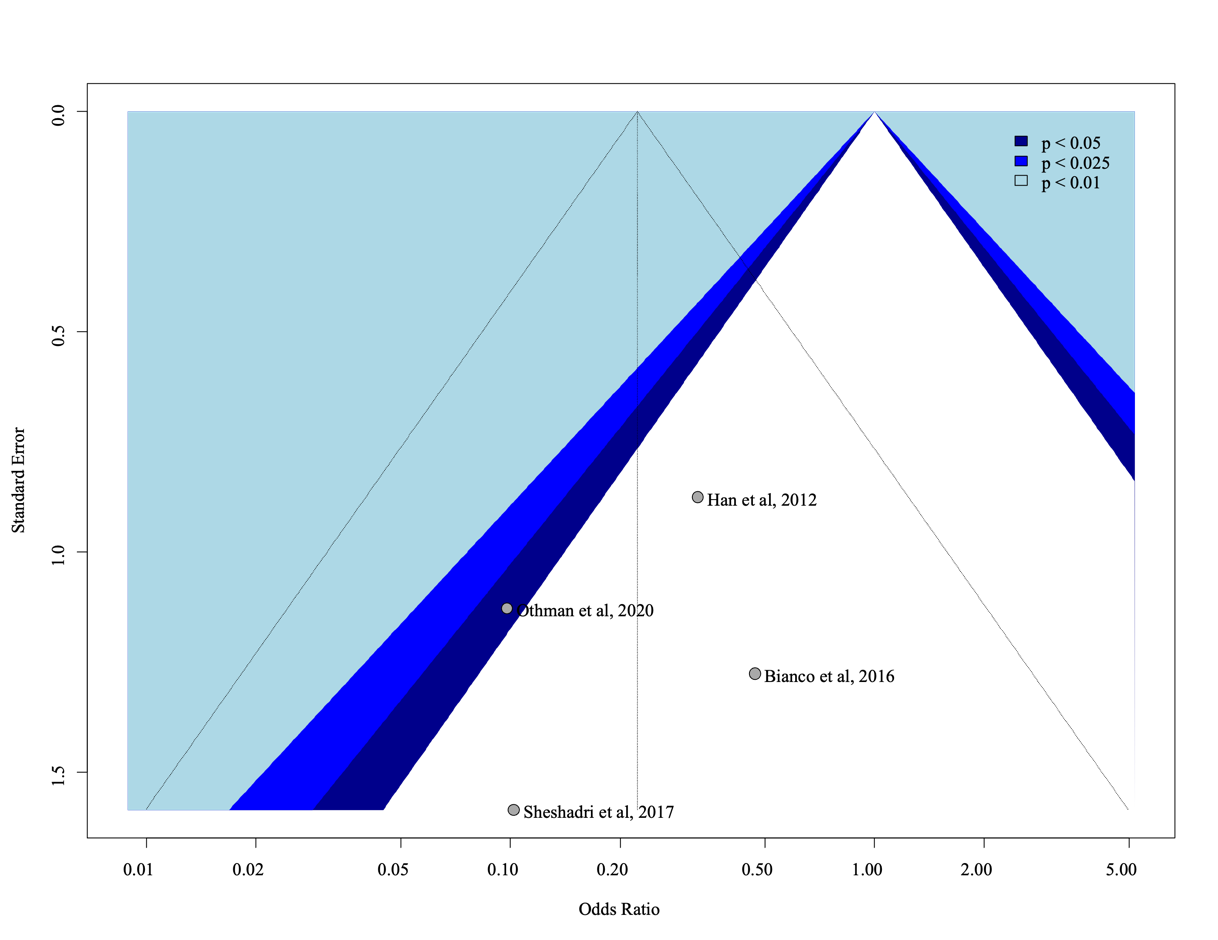


**Figure S42:** Funnel plot for positive CRM rates following extralevator abdominoperineal excision compared to standard abdominoperineal excision


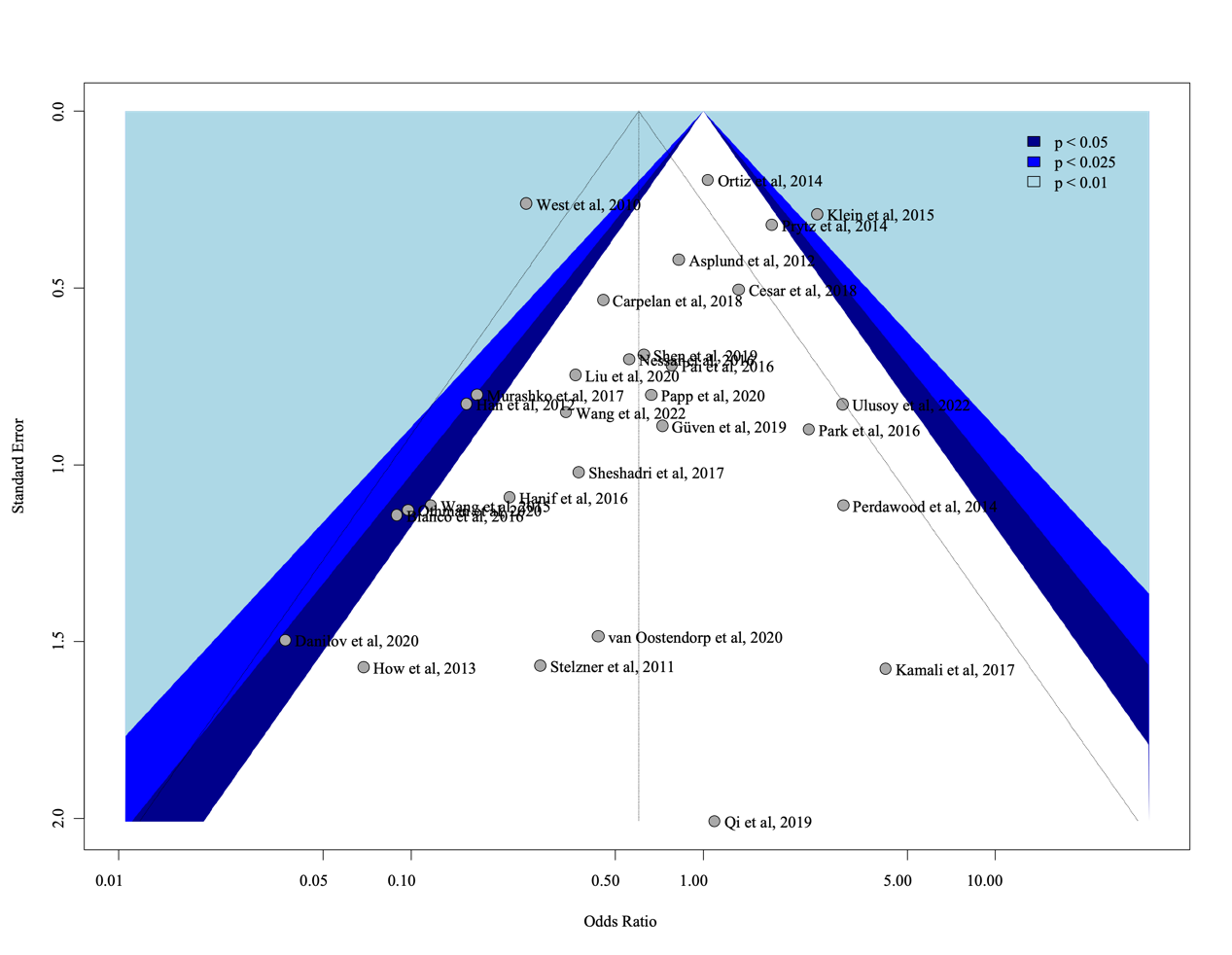


**Figure S43:** Funnel plot for positive CRM rates following extralevator abdominoperineal excision compared to standard abdominoperineal excision - subgroup of RCT’s


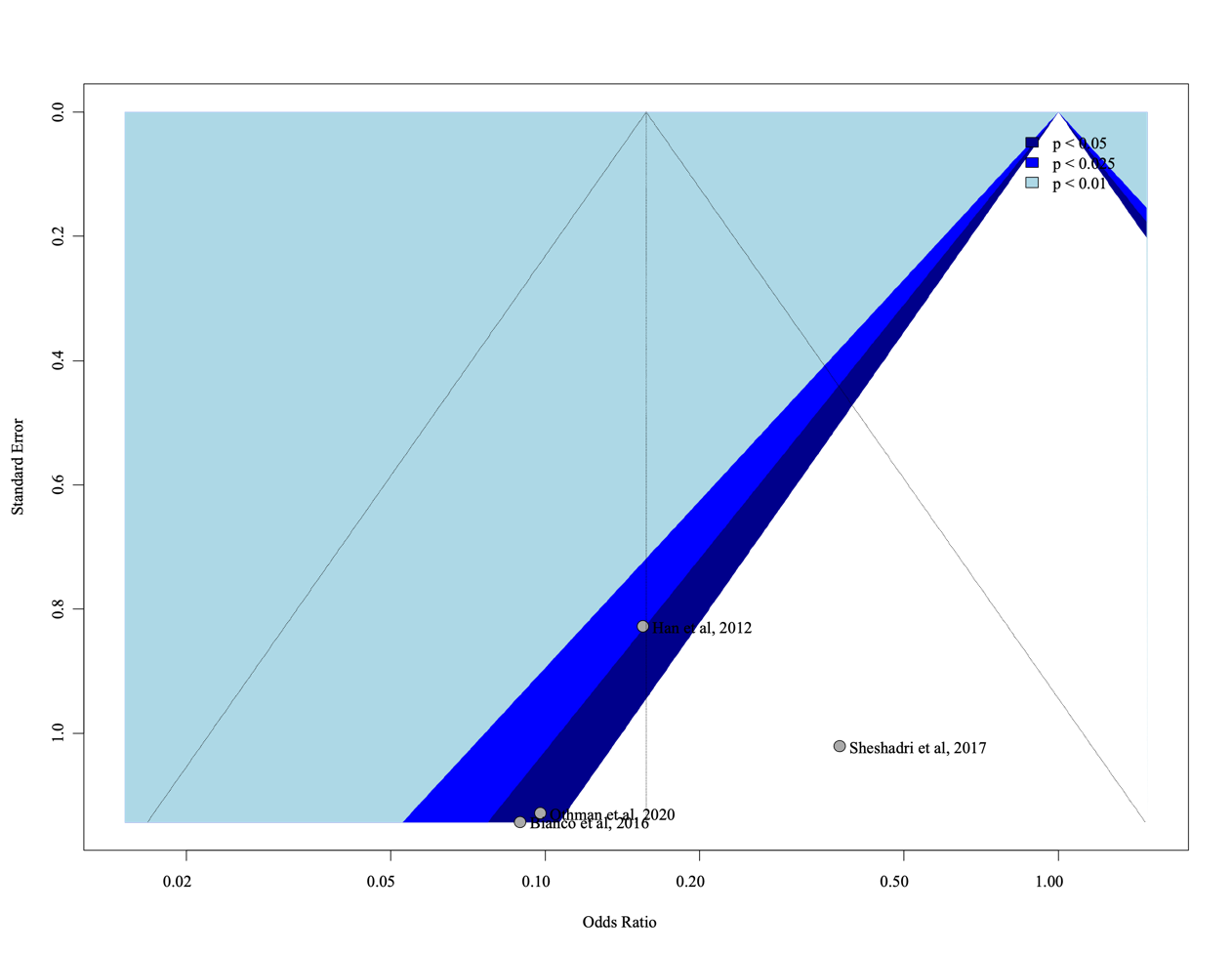


**Figure S44:** Funnel plot for the difference in blood loss during extralevator abdominoperineal excision compared to standard abdominoperineal excision


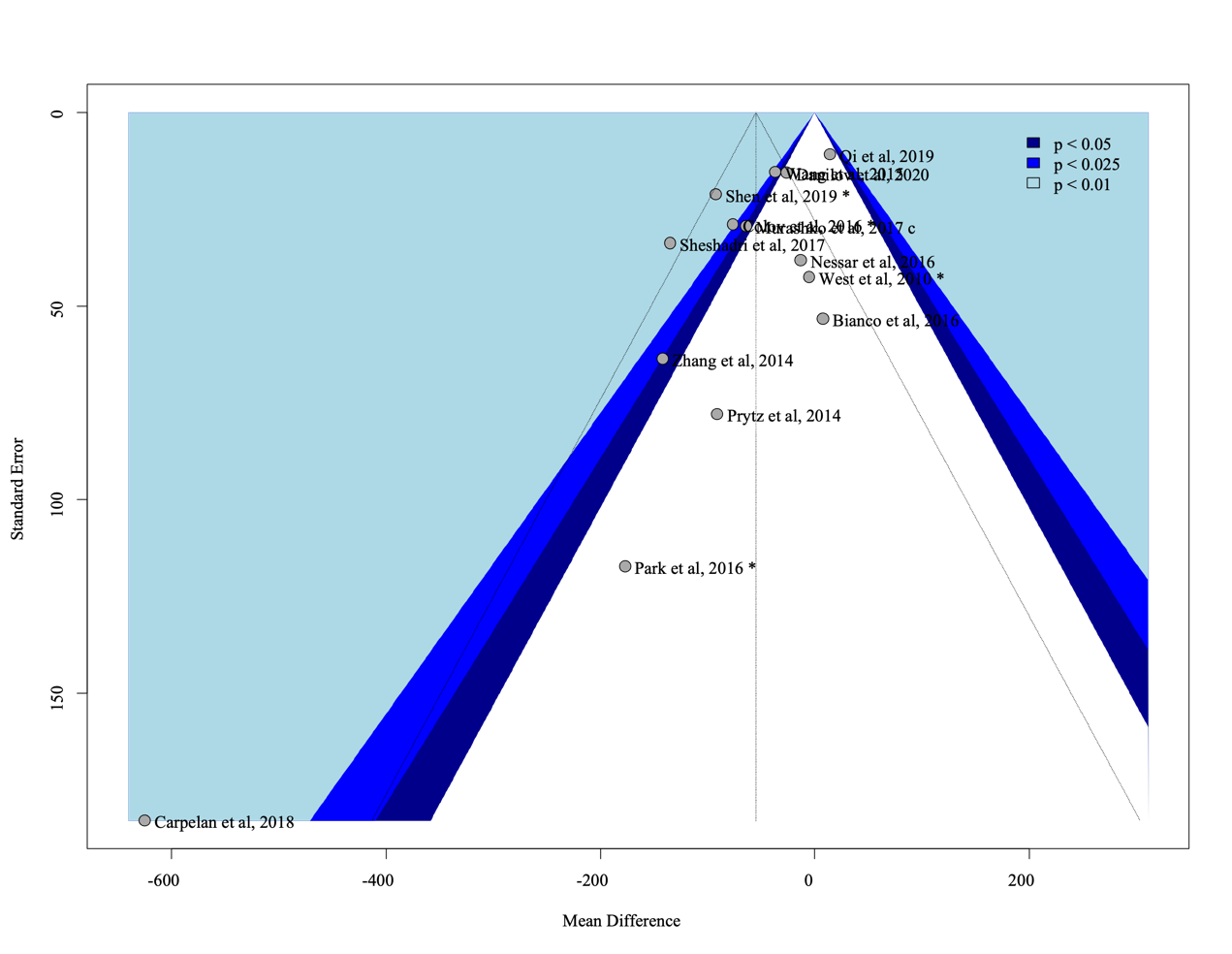


**Figure S45:** Funnel plot for the difference in operative time during extralevator abdominoperineal excision compared to standard abdominoperineal excision


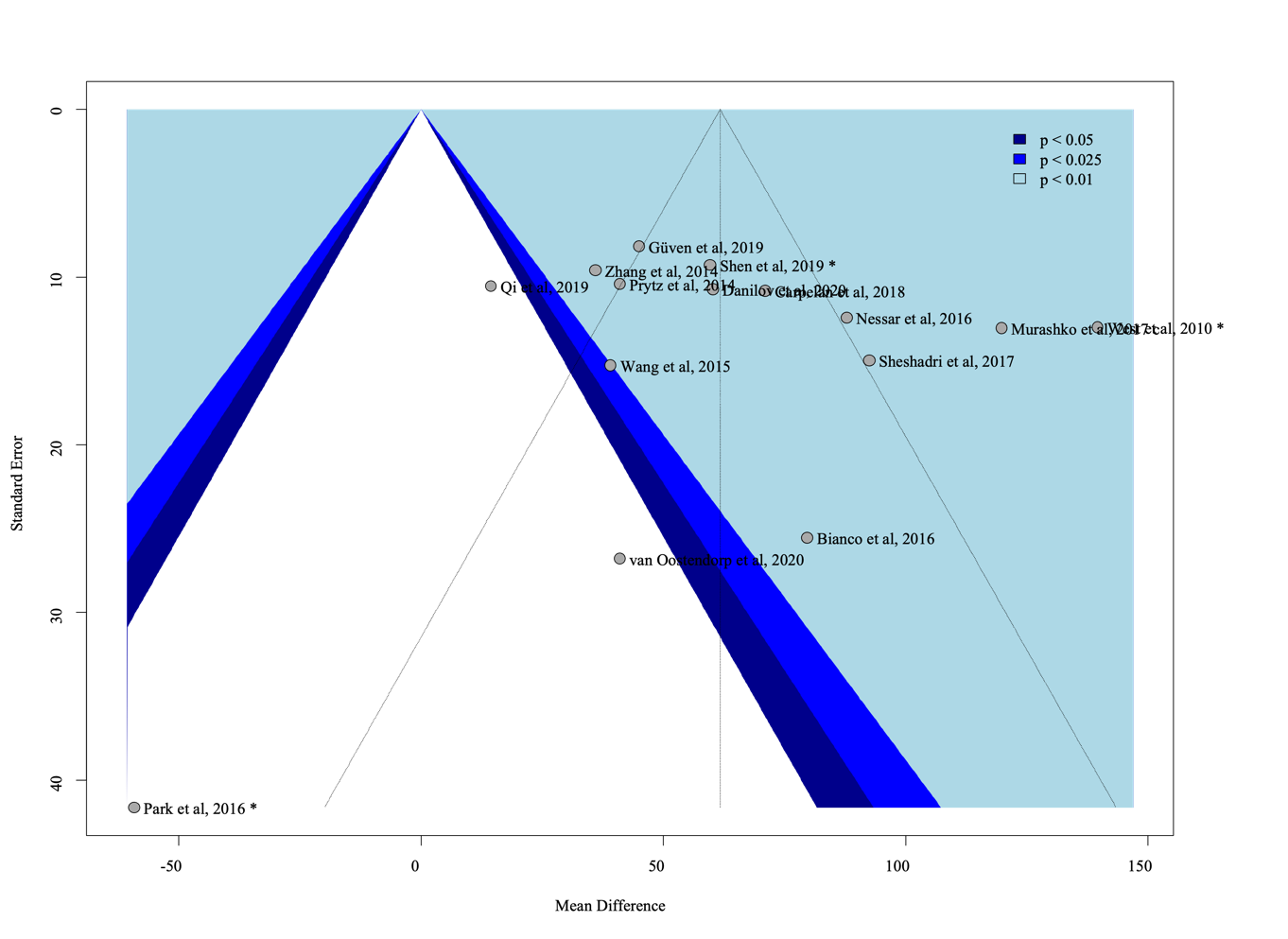


**Figure S46:** Funnel plot for mortality following extralevator abdominoperineal excision compared to standard abdominoperineal excision


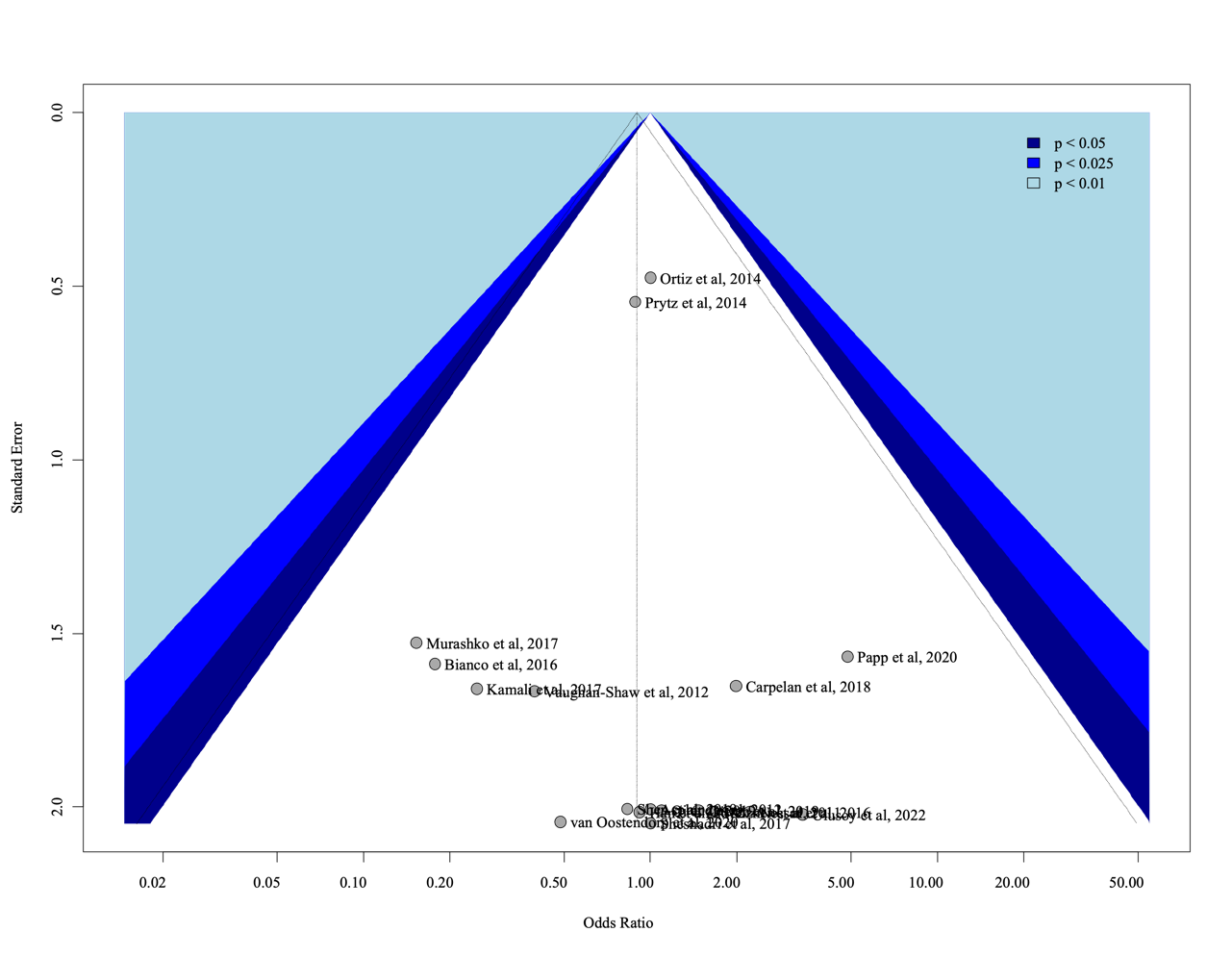


**Figure S47:** Funnel plot for mortality following extralevator abdominoperineal excision compared to standard abdominoperineal excision – subgroup of RCT’s


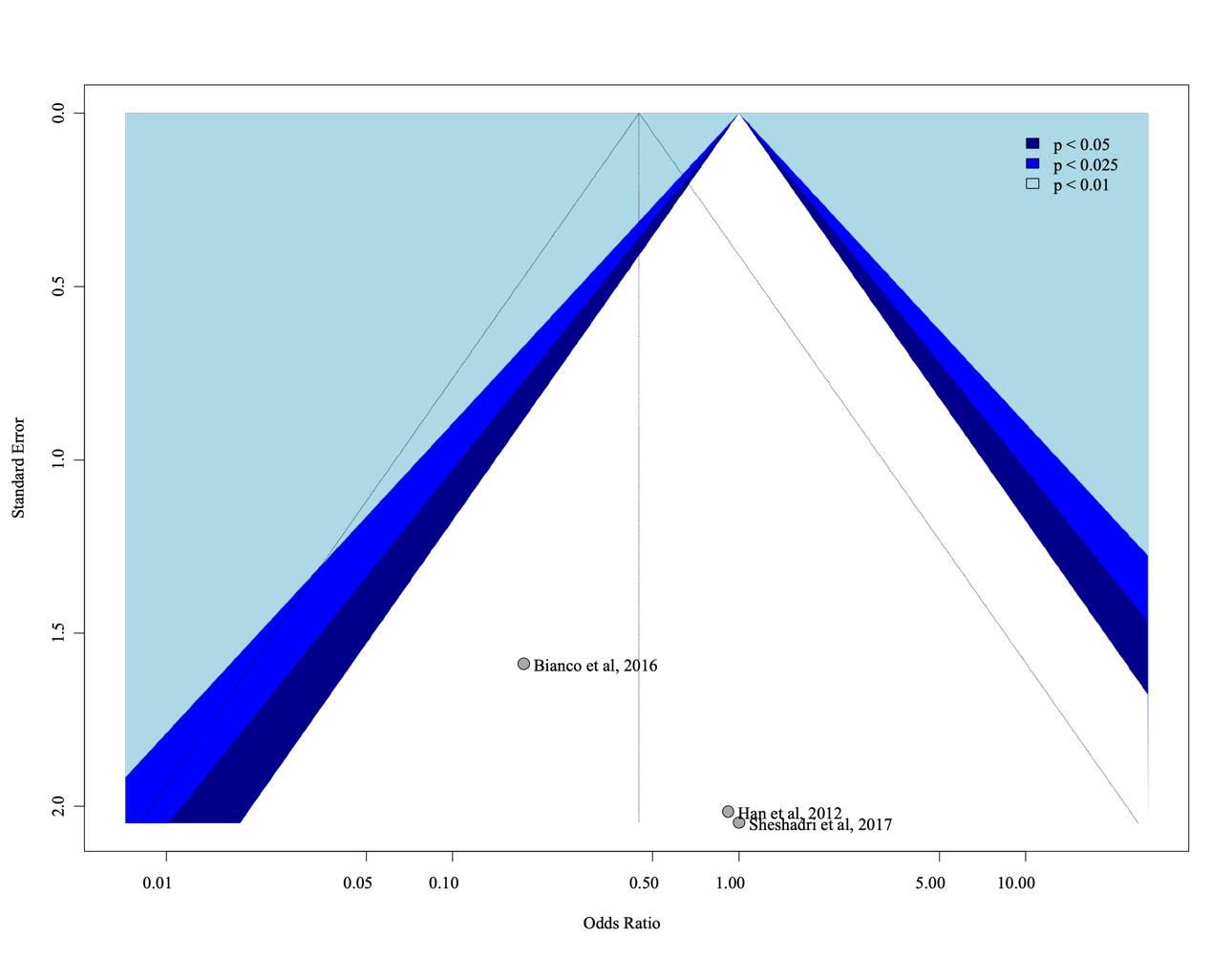


**Figure S48:** Funnel plot for overall complications following extralevator abdominoperineal excision compared to standard abdominoperineal excision


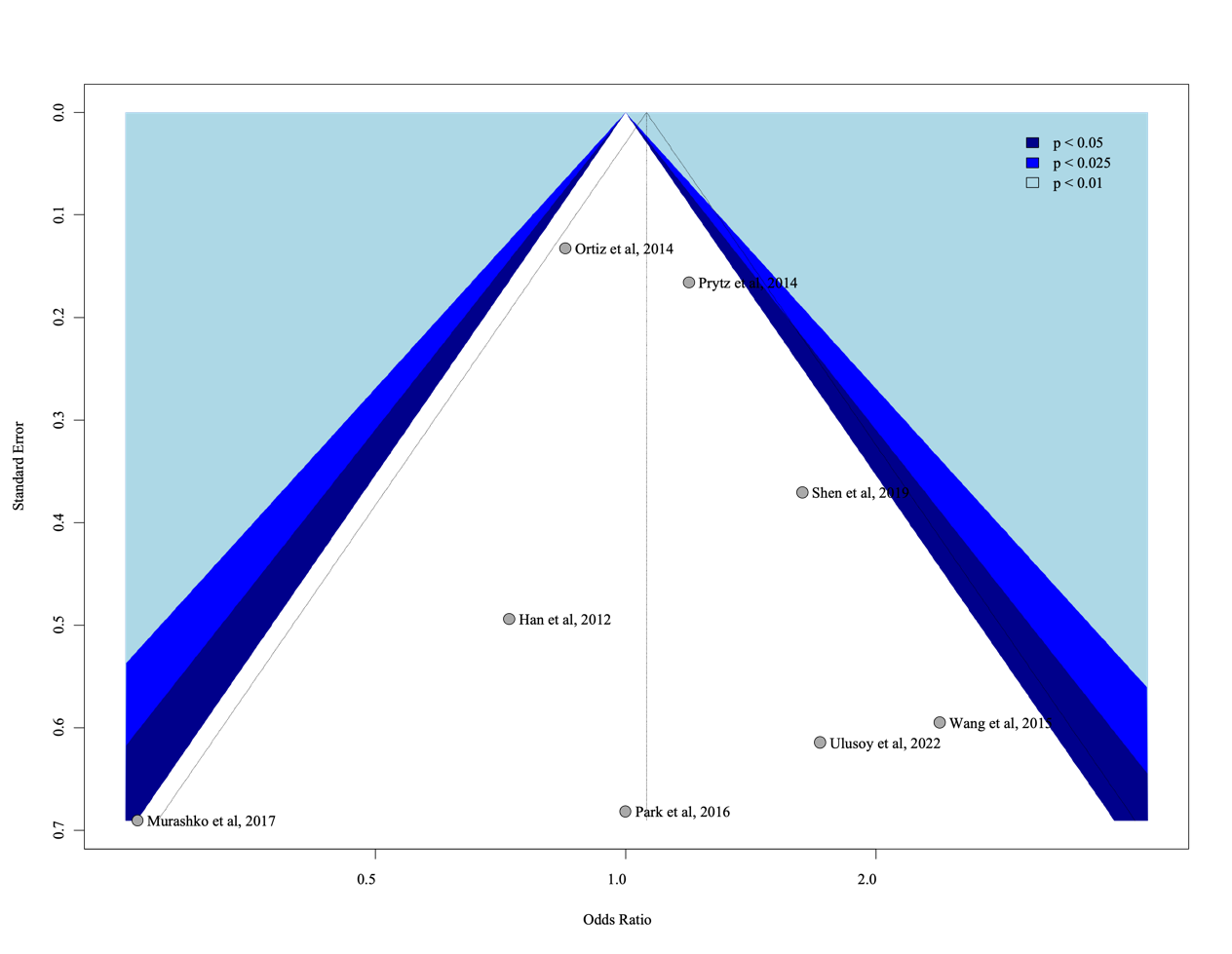


**Figure S49:** Funnel plot for reoperation rates following extralevator abdominoperineal excision compared to standard abdominoperineal excision


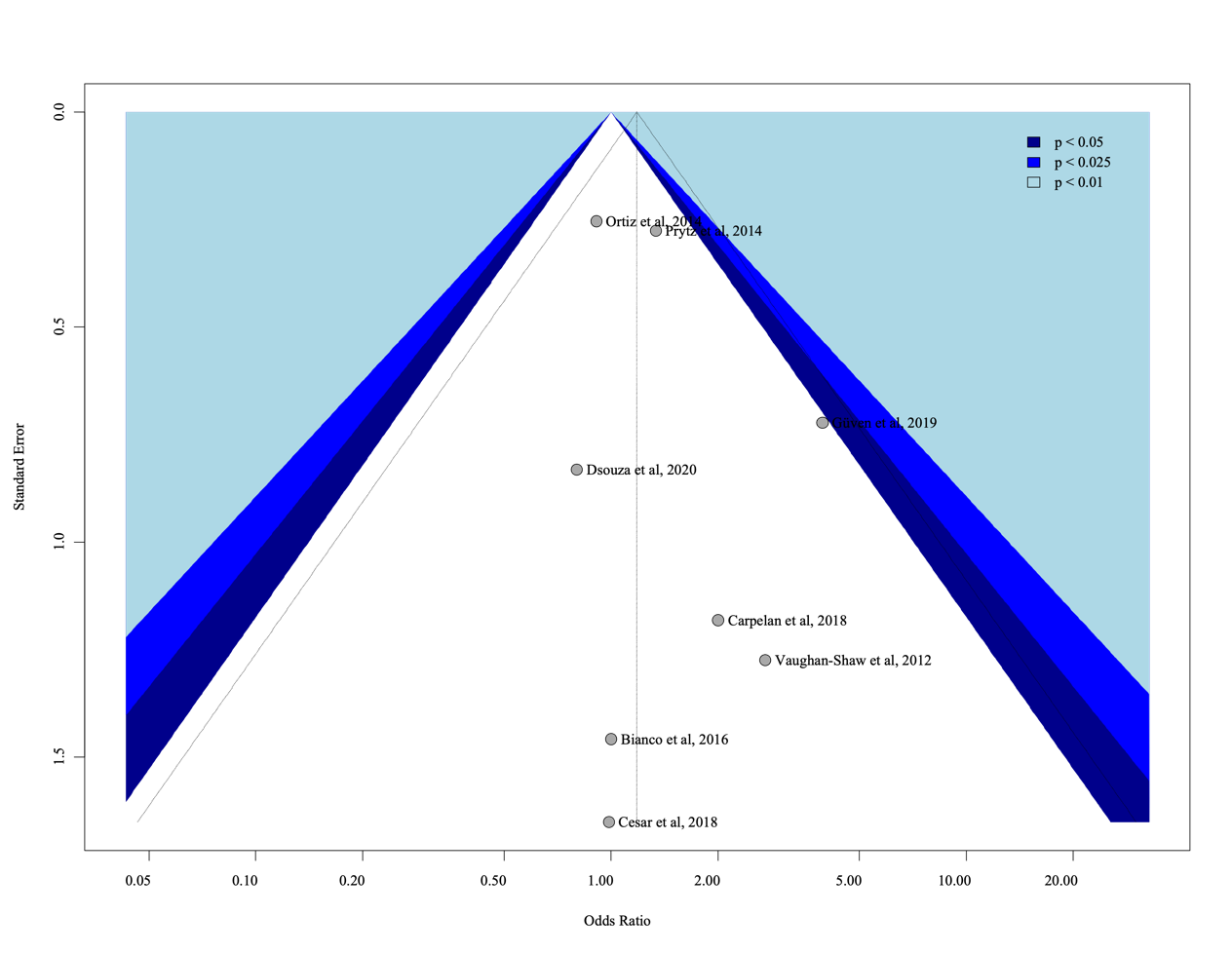


**Figure S50:** Funnel plot for hospital stay following extralevator abdominoperineal excision compared to standard abdominoperineal excision


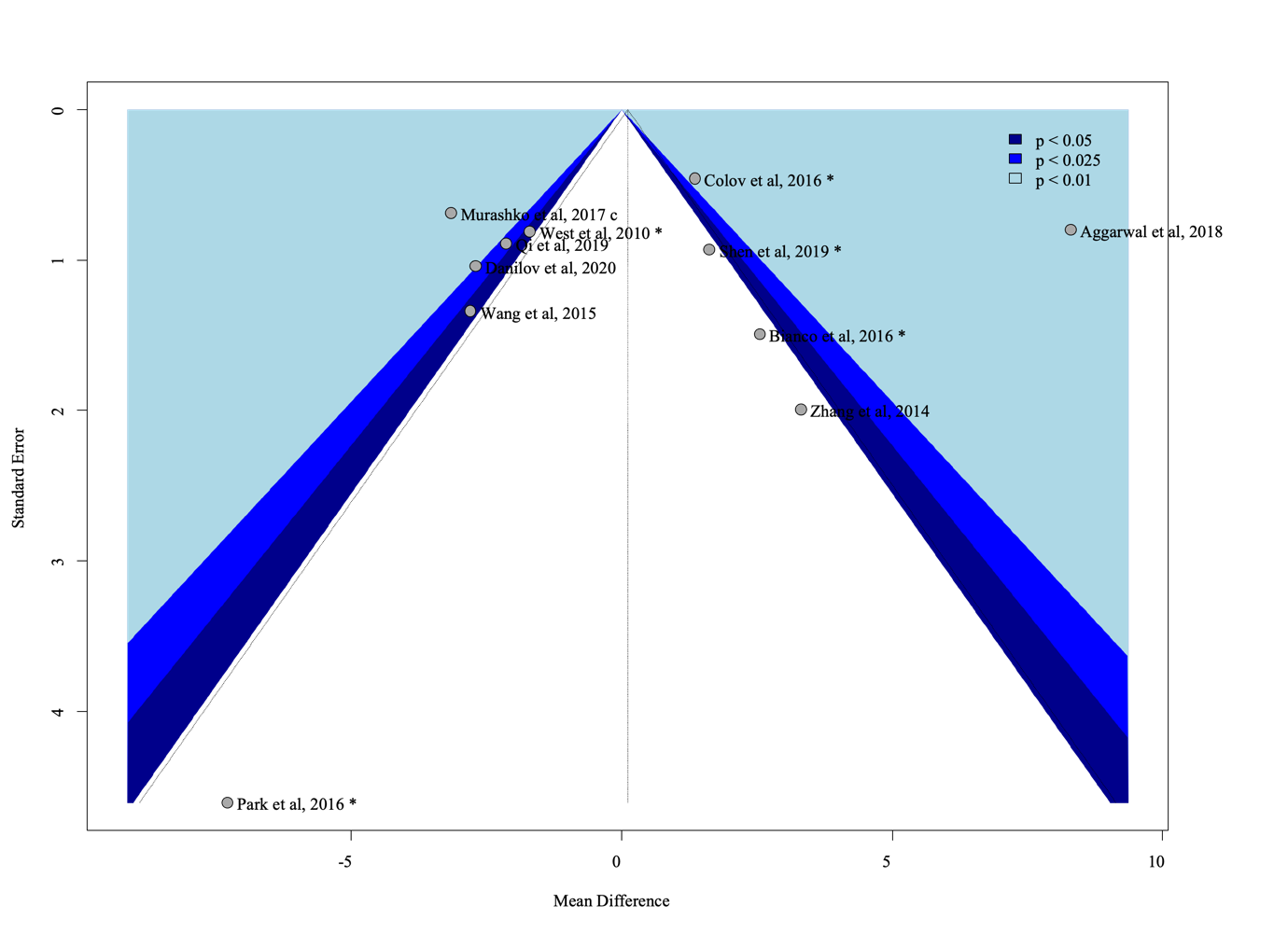


**Figure S51:** Funnel plot for perineal wound complications following extralevator abdominoperineal excision compared to standard abdominoperineal excision


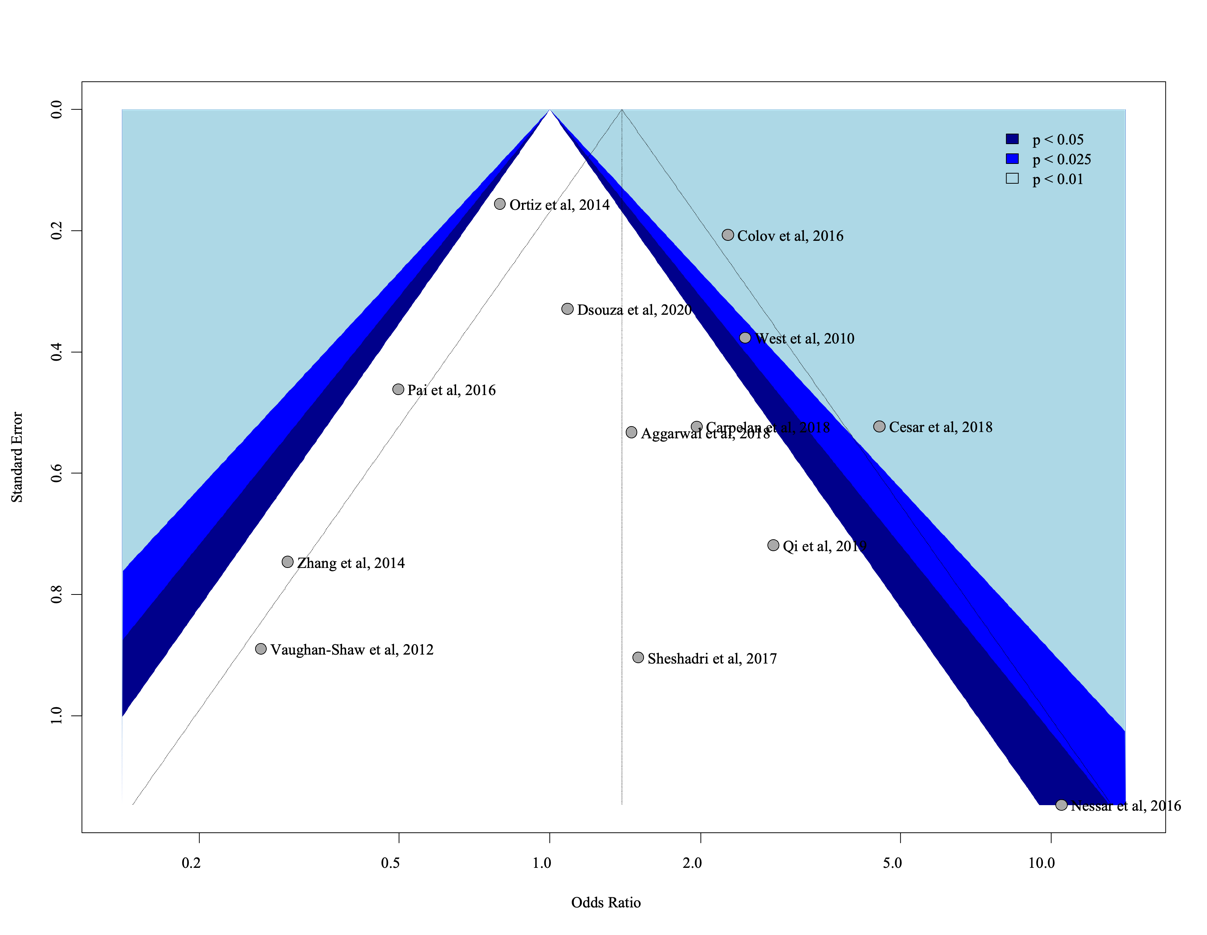


**Figure S52:** Funnel plot for perineal wound infections following extralevator abdominoperineal excision compared to standard abdominoperineal excision


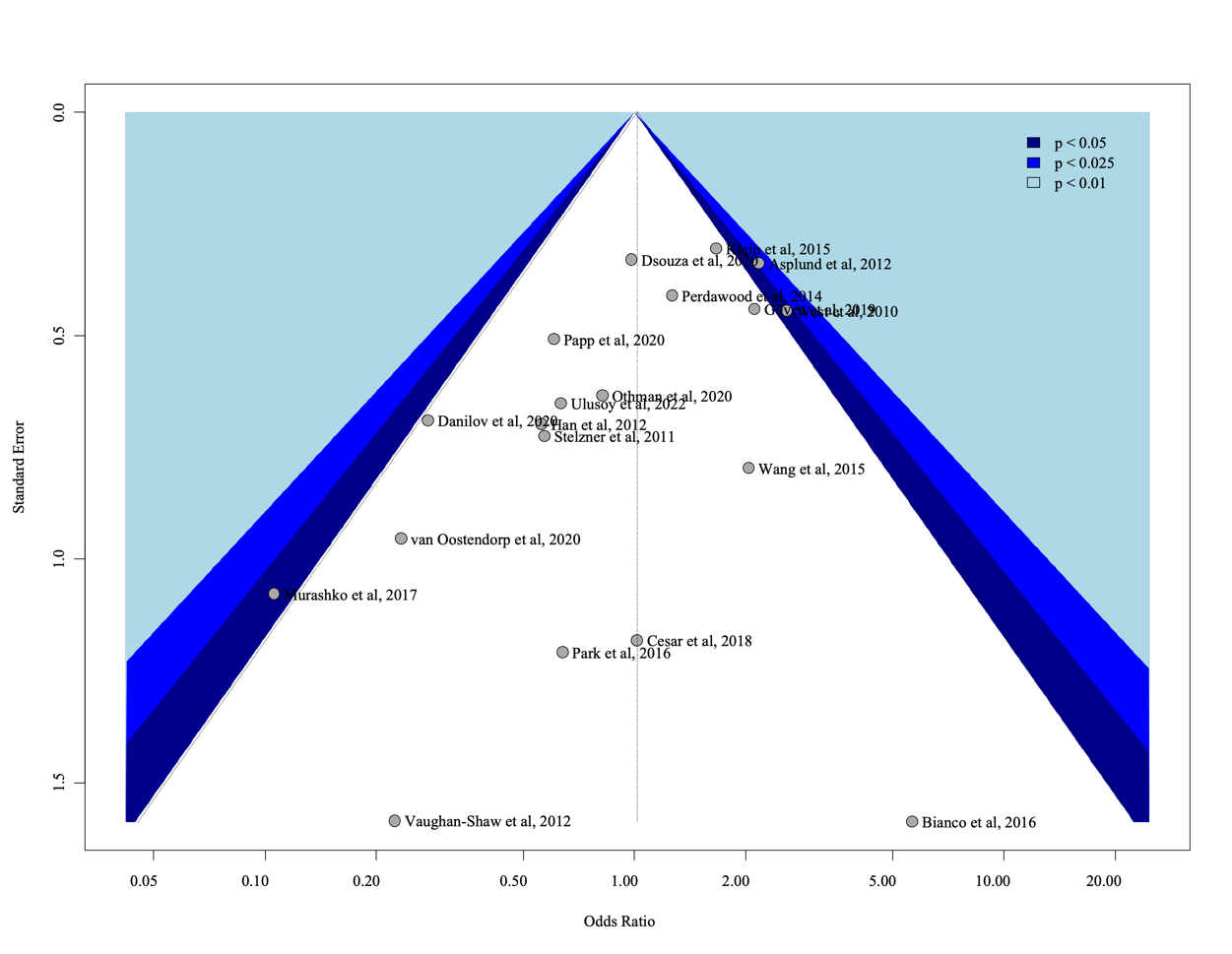


**Figure S53:** Funnel plot for perineal wound infections following extralevator abdominoperineal excision compared to standard abdominoperineal excision – subgroup for RCT’s


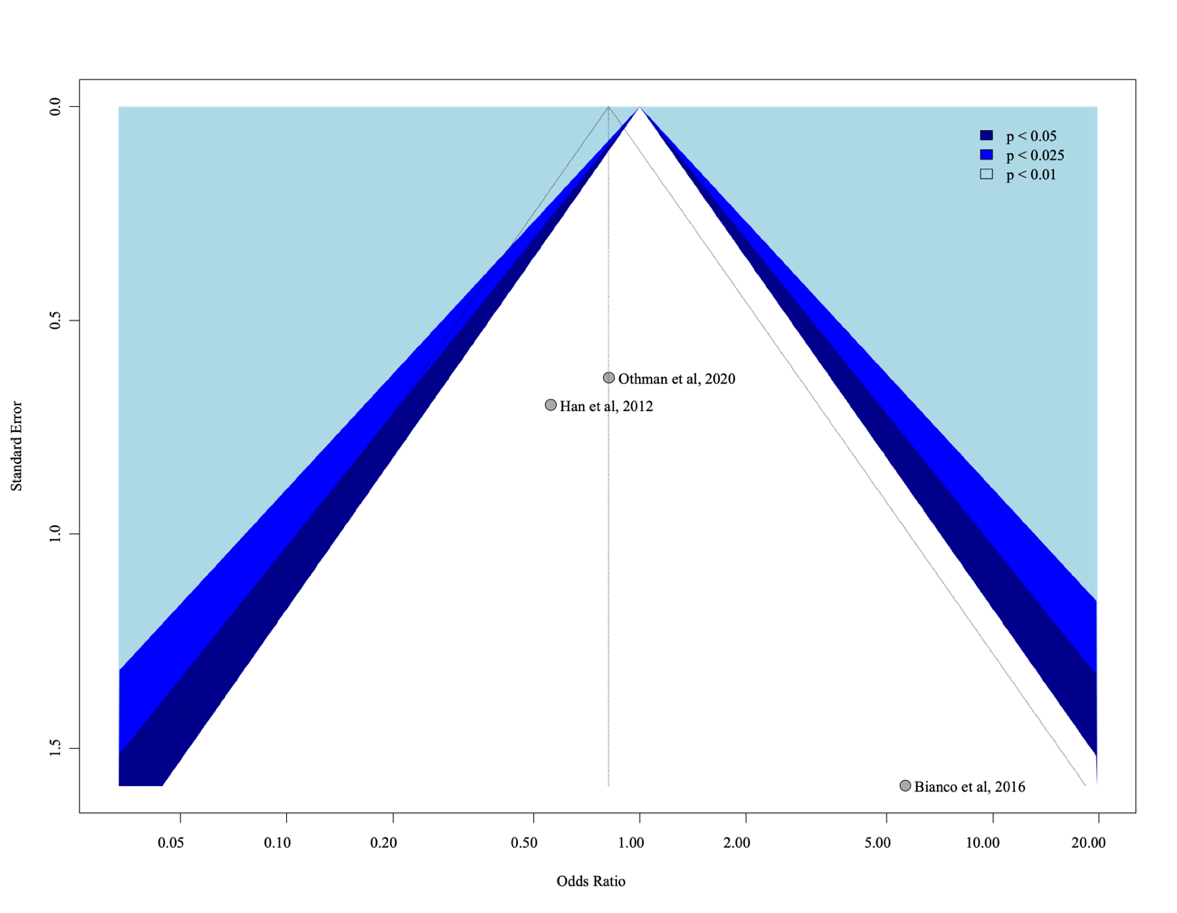


**Figure S54:** Funnel plot for perineal wound dehiscence following extralevator abdominoperineal excision compared to standard abdominoperineal excision


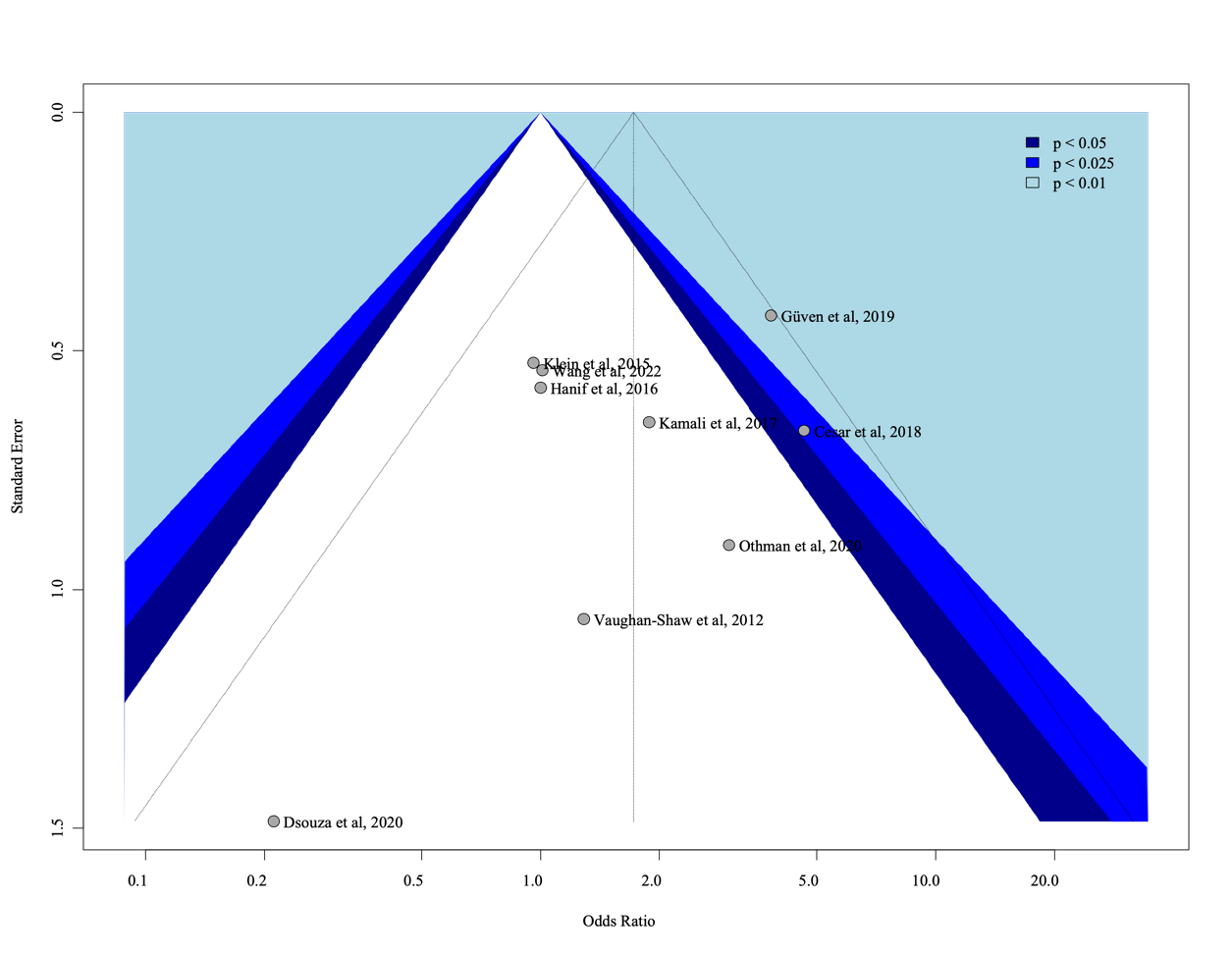


**Figure S55:** Funnel plot for perineal hernia following extralevator abdominoperineal excision compared to standard abdominoperineal excision
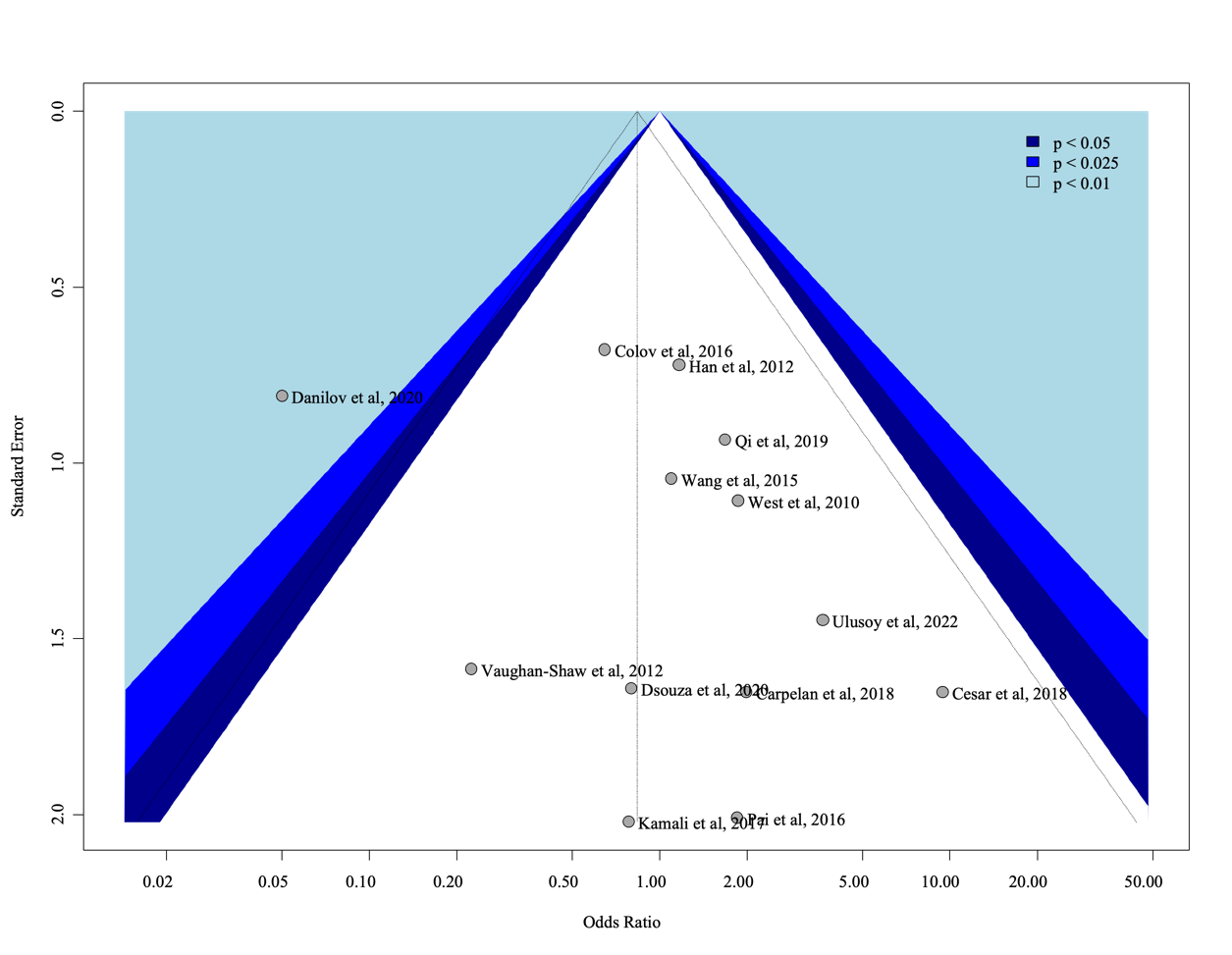


**Figure S56:** Funnel plot for the existence of perineal wound pain following extralevator abdominoperineal excision compared to standard abdominoperineal excision


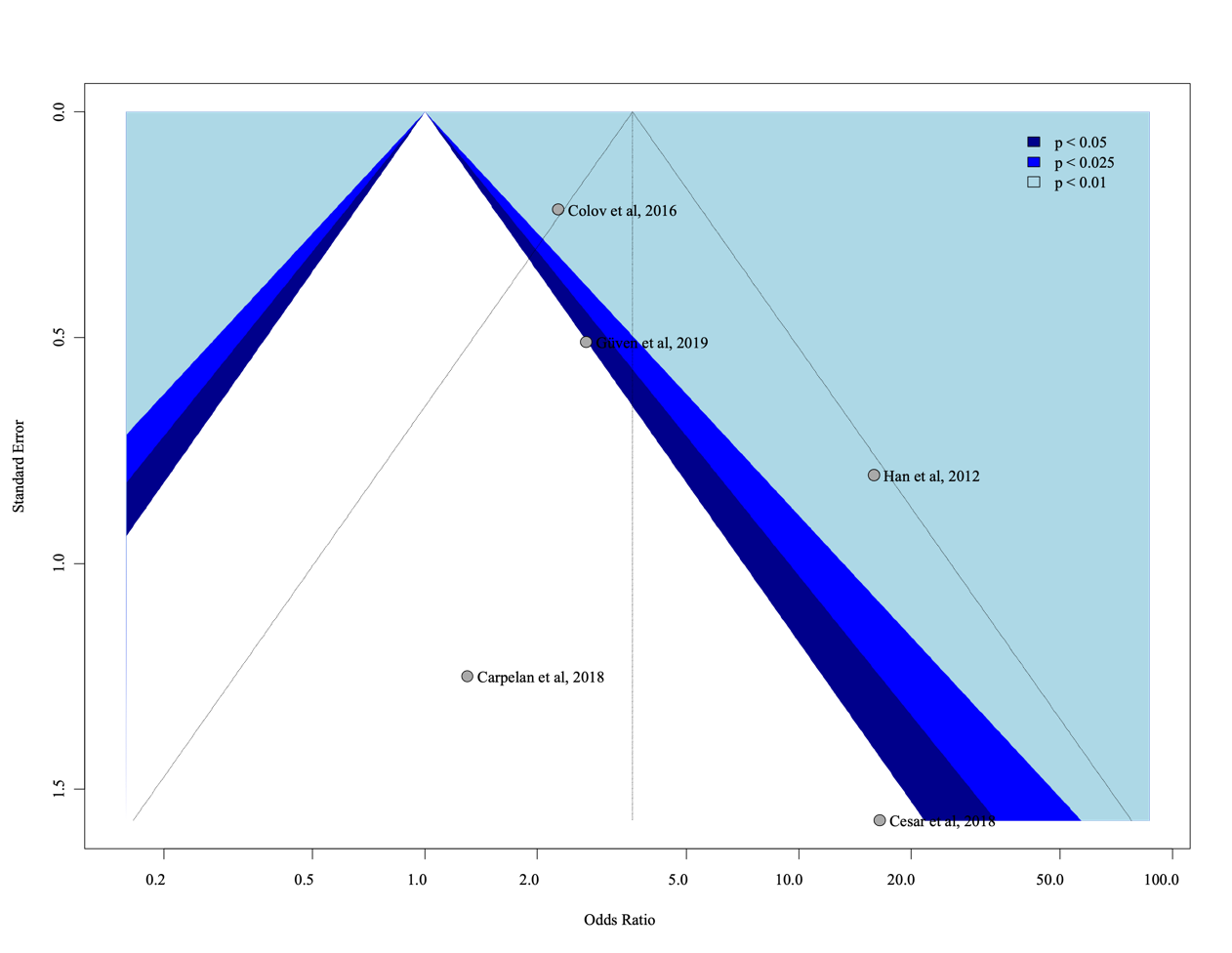


**Table S6:** Detailed assessment of the certainty of the evidence for the comparison of ELAPE to APE

| **Certainty assessment** | | | | | | | **№ of patients** | | **Effect** | | **Certainty** | **Importance** |
| --- | --- | --- | --- | --- | --- | --- | --- | --- | --- | --- | --- | --- |
| **№ of studies** | **Study design** | **Risk of bias** | **Inconsistency** | **Indirectness** | **Imprecision** | **Other considerations** | **ELAPE** | **APE** | **Relative (95% CI)** | **Absolute (95% CI)** |  |  |
| **Overall survival (assessed with: HR)** | | | | | | | | | | | | |
| 7 | non-randomised studies | serious | serious | not serious | serious | none | 437 participants | 394 participants | **HR 0.539** (0.400 to 0.728) [Overall survival] | **-- per 1,000** (from -- to --) | ⨁◯◯◯ Very low |  |
|  |  |  |  |  |  |  | - | 0.0% |  | **-- per 1,000** (from -- to --) |  |  |
| **Disease-free survival** | | | | | | | | | | | | |
| 5 | non-randomised studies | serious | serious | not serious | serious | none | 244 participants | 291 participants | **HR 0.738** (0.550 to 0.990) [survival] | **-- per 1,000** (from -- to --) | ⨁◯◯◯ Very low |  |
|  |  |  |  |  |  |  | - | 0.0% |  | **-- per 1,000** (from -- to --) |  |  |
| **Local recurrence-free survival** | | | | | | | | | | | | |
| 4 | non-randomised studies | serious | serious | not serious | serious | none | 719 participants | 668 participants | **HR 0.671** (0.447 to 1.006) [survival] | **-- per 1,000** (from -- to --) | ⨁◯◯◯ Very low |  |
|  |  |  |  |  |  |  | - | 0.0% |  | **-- per 1,000** (from -- to --) |  |  |
| **Intraoperative perforation** | | | | | | | | | | | | |
| 27 | observational studies | serious | serious | not serious | not serious | none | 138/2241 (6.2%) | 211/1932 (10.9%) | **OR 0.49** (0.34 to 0.72) | **53 fewer per 1,000** (from 69 fewer to 28 fewer) | ⨁⨁◯◯ Low |  |
| **Intraoperative perforation - subgroup RCT** | | | | | | | | | | | | |
| 4 | randomised trials | not serious | not serious | not serious | serious | none | 4/82 (4.9%) | 17/79 (21.5%) | **OR 0.22** (0.07 to 0.73) | **158 fewer per 1,000** (from 196 fewer to 48 fewer) | ⨁⨁⨁◯ Moderate |  |
| **Positive circumferential resection margin** | | | | | | | | | | | | |
| 30 | observational studies | serious | serious | not serious | not serious | none | 274/2423 (11.3%) | 318/2116 (15.0%) | **OR 0.6** (0.4 to 0.9) | **54 fewer per 1,000** (from 84 fewer to 13 fewer) | ⨁⨁◯◯ Low |  |
| **Positive circumferential resection margin - subgroup RCT** | | | | | | | | | | | | |
| 4 | randomised trials | not serious | not serious | not serious | serious | none | 6/82 (7.3%) | 27/79 (34.2%) | **OR 0.16** (0.06 to 0.42) | **265 fewer per 1,000** (from 312 fewer to 163 fewer) | ⨁⨁⨁◯ Moderate |  |
| **Blood loss** | | | | | | | | | | | | |
| 14 | observational studies | serious | serious | not serious | not serious | none | 1365 | 946 | - | MD **54.68 ml lower** (95.42 lower to 13.93 lower) | ⨁⨁◯◯ Low |  |
| **Operative time** | | | | | | | | | | | | |
| 15 | observational studies | serious | serious | not serious | not serious | none | 1179 | 816 | - | MD **61.73 minutes higher** (38.7 higher to 84.76 higher) | ⨁⨁◯◯ Low |  |
| **Mortality** | | | | | | | | | | | | |
| 18 | observational studies | serious | serious | not serious | not serious | none | 23/1567 (1.5%) | 21/1325 (1.6%) | **OR 0.90** (0.64 to 1.26) | **2 fewer per 1,000** (from 6 fewer to 4 more) | ⨁⨁◯◯ Low |  |
| **Mortality - subgroup RCT** | | | | | | | | | | | | |
| 3 | randomised trials | not serious | not serious | not serious | serious | none | 0/62 (0.0%) | 2/59 (3.4%) | **OR 0.45** (0.03 to 5.76) | **18 fewer per 1,000** (from 33 fewer to 134 more) | ⨁⨁⨁◯ Moderate |  |
| **Overall complications** | | | | | | | | | | | | |
| 8 | observational studies | serious | serious | not serious | serious | none | 535/1209 (44.3%) | 421/937 (44.9%) | **OR 1.06** (0.69 to 1.63) | **14 more per 1,000** (from 89 fewer to 121 more) | ⨁◯◯◯ Very low |  |
| **Reoperation** | | | | | | | | | | | | |
| 8 | observational studies | serious | serious | not serious | not serious | none | 108/1178 (9.2%) | 67/997 (6.7%) | **OR 1.18** (0.84 to 1.66) | **11 more per 1,000** (from 10 fewer to 40 more) | ⨁⨁◯◯ Low |  |
| **Hospital stay** | | | | | | | | | | | | |
| 11 | observational studies | serious | serious | not serious | serious | none | 791 | 694 | - | MD **0.11 days higher** (0 to 0 ) | ⨁◯◯◯ Very low |  |
| **Perineal wound complications - overall** | | | | | | | | | | | | |
| 13 | observational studies | serious | serious | not serious | serious | none | 370/1173 (31.5%) | 354/1244 (28.5%) | **OR 1.39** (0.81 to 2.40) | **71 more per 1,000** (from 41 fewer to 204 more) | ⨁◯◯◯ Very low |  |
| **Perineal wound infection** | | | | | | | | | | | | |
| 19 | observational studies | serious | serious | not serious | not serious | none | 229/1045 (21.9%) | 214/1052 (20.3%) | **OR 1.02** (0.69 to 1.50) | **3 more per 1,000** (from 54 fewer to 74 more) | ⨁⨁◯◯ Low |  |
| **Perineal wound infection - subgroup RCT** | | | | | | | | | | | | |
| 3 | randomised trials | not serious | not serious | not serious | serious | none | 16/72 (22.2%) | 17/69 (24.6%) | **OR 0.82** (0.13 to 5.06) | **35 fewer per 1,000** (from 206 fewer to 377 more) | ⨁⨁⨁◯ Moderate |  |
| **Perineal wound dehiscence** | | | | | | | | | | | | |
| 9 | observational studies | serious | serious | not serious | not serious | none | 370/1173 (31.5%) | 354/1244 (28.5%) | **OR 1.39** (0.81 to 2.40) | **71 more per 1,000** (from 41 fewer to 204 more) | ⨁⨁◯◯ Low |  |
| **Perineal hernia** | | | | | | | | | | | | |
| 13 | observational studies | serious | serious | not serious | serious | none | 24/789 (3.0%) | 30/803 (3.7%) | **OR 0.84** (0.37 to 1.87) | **6 fewer per 1,000** (from 23 fewer to 30 more) | ⨁◯◯◯ Very low |  |
| **Perineal pain** | | | | | | | | | | | | |
| 5 | observational studies | serious | serious | not serious | serious | none | 128/306 (41.8%) | 54/388 (13.9%) | **OR 3.59** (1.10 to 11.74) | **228 more per 1,000** (from 12 more to 516 more) | ⨁◯◯◯ Very low |  |

**CI:** confidence interval; **HR:** hazard Ratio; **MD:** mean difference; **OR:** odds ratio

**References**: 1. Carpelan A, Karvonen J, Varpe P, Rantala A, Kaljonen A, Grönroos J, et al. Extralevator versus standard abdominoperineal excision in locally advanced rectal cancer: a retrospective study with long-term follow-up. Int J Colorectal Dis. 2018 Apr;33(4):375–81.

2. Ortiz H, Ciga MA, Armendariz P, Kreisler E, Codina-Cazador A, Gomez-Barbadillo J, et al. Multicentre propensity score-matched analysis of conventional *versus* extended abdominoperineal excision for low rectal cancer. Br J Surg. 2014 May 12;101(7):874–82.

3. Pai VD, Engineer R, Patil PS, Arya S, Desouza AL, Saklani AP. Selective extra levator versus conventional abdomino perineal resection: experience from a tertiary-care center. J Gastrointest Oncol. 2016 Jun;7(3):354–9.

4. Seshadri RA, West NP, Sundersingh S. A pilot randomized study comparing extralevator with conventional abdominoperineal excision for low rectal cancer after neoadjuvant chemoradiation. Colorectal Dis [Internet]. 2017 Jul [cited 2024 Oct 17];19(7). Available from: https://onlinelibrary.wiley.com/doi/10.1111/codi.13726

5. Othman, El-Khatib, Shehata. Extra-Levator Abdomino-Perineal Excision Versus Standard Abdomino-Perineal Excision: A Prospective Study in the Egyptian National Cancer Institute. Indian J Public Health Res Dev. 11/3(March 2020):2572–7.

6. Vaughan-Shaw PG, Cheung T, Knight JS, Nichols PH, Pilkington SA, Mirnezami AH. A prospective case–control study of extralevator abdominoperineal excision (ELAPE) of the rectum versus conventional laparoscopic and open abdominoperineal excision: comparative analysis of short-term outcomes and quality of life. Tech Coloproctology. 2012 Oct;16(5):355–62.

7. Murashko, R.A., Uvarov, I.B., Ermakov, E.A., Kaushanskiy, V.B., Konkov, R.V., Sichinava, D.D., et al. Extralevator Abdominoperineal Excision of the Rectum: Short-term Outcomes in Comparison with Conventional Surgery. КОЛОПРОКТОЛОГИЯ. 2017;4(62):34–9.

8. Rohatgi A. Webplotdigitizer [Internet]. 2022. Available from: https://automeris.io/WebPlotDigitizer

9. Luo D, Wan X, Liu J, Tong T. Optimally estimating the sample mean from the sample size, median, mid-range, and/or mid-quartile range. Stat Methods Med Res. 2018 Jun;27(6):1785–805.

10. Shi J, Luo D, Weng H, Zeng X, Lin L, Chu H, et al. Optimally estimating the sample standard deviation from the five‐number summary. Res Synth Methods. 2020 Jul 25;jrsm.1429.

11. Mantel, Nathan, and William Haenszel. Statistical Aspects of the Analysis of Data From Retrospective Studies of Disease. JNCI J Natl Cancer Inst [Internet]. 1959 Apr [cited 2023 May 29]; Available from: https://academic.oup.com/jnci/article/22/4/719/900746/Statistical-Aspects-of-the-Analysis-of-Data-From

12. Robins J, Greenland S, Breslow NE. A general estimator for the variance of the mantel-haenszel odds ratio. American Journal of Epidemiology. 1986;719–23.

13. Cooper HM, Hedges LV, Valentine JC, editors. The handbook of research synthesis and meta-analysis. 2nd ed. New York: Russell Sage Foundation; 2009.

14. J. Sweeting M, J. Sutton A, C. Lambert P. What to add to nothing? Use and avoidance of continuity corrections in meta-analysis of sparse data. Stat Med. 2004 May 15;23(9):1351–75.

15. Knapp G, Hartung J. Improved tests for a random effects meta-regression with a single covariate. Stat Med. 2003 Sep 15;22(17):2693–710.

16. IntHout J, Ioannidis JP, Borm GF. The Hartung-Knapp-Sidik-Jonkman method for random effects meta-analysis is straightforward and considerably outperforms the standard DerSimonian-Laird method. BMC Med Res Methodol. 2014 Dec;14(1):25.

17. Paule, Robert, and John Mandel. Consensus Values and Weighting Factors. J Res Natl Bur Stand. 1982;87(5):377–87.

18. Harrer, Mathias, Pim Cuijpers, Furukawa Toshi A, and David D Ebert. Doing Meta-Analysis With R: A Hands-On Guide. 1st ed. Boca Raton FL Lond Chapman HallCRC Press. 2021;

19. Veroniki AA, Jackson D, Viechtbauer W, Bender R, Bowden J, Knapp G, et al. Methods to estimate the between‐study variance and its uncertainty in meta‐analysis. Res Synth Methods. 2016 Mar;7(1):55–79.

20. Jackson D, Law M, Rücker G, Schwarzer G. The Hartung‐Knapp modification for random‐effects meta‐analysis: A useful refinement but are there any residual concerns? Stat Med. 2017 Nov 10;36(25):3923–34.

21. Kaplan EL, Meier P. Nonparametric Estimation from Incomplete Observations. J Am Stat Assoc. 1958 Jun;53(282):457–81.

22. Cheung MWL. Modeling dependent effect sizes with three-level meta-analyses: A structural equation modeling approach. Psychol Methods. 2014;19(2):211–29.

23. Combescure C, Foucher Y, Jackson D. Meta-analysis of single-arm survival studies: a distribution-free approach for estimating summary survival curves with random effects. Stat Med. 2014 Jul 10;33(15):2521–37.

24. Klein M, Colov E, Gögenur I. Similar long-term overall and disease-free survival after conventional and extralevator abdominoperineal excision—a nationwide study. Int J Colorectal Dis. 2016 Jul;31(7):1341–7.

25. Prytz M, Angenete E, Bock D, Haglind E. Extralevator Abdominoperineal Excision for Low Rectal Cancer—Extensive Surgery to Be Used With Discretion Based on 3-Year Local Recurrence Results: A Registry-based, Observational National Cohort Study. Ann Surg. 2016 Mar;263(3):516–21.

26. Aggarwal N, Seshadri RA, Arvind A, Jayanand SB. Perineal Wound Complications Following Extralevator Abdominoperineal Excision: Experience of a Regional Cancer Center. Indian J Surg Oncol. 2018 Jun;9(2):211–4.

27. Asplund D, Haglind E, Angenete E. Outcome of extralevator abdominoperineal excision compared with standard surgery: results from a single centre. Colorectal Dis. 2012 Oct;14(10):1191–6.

28. Bianco F, Romano G, Tsarkov P, Stanojevic G, Shroyer K, Giuratrabocchetta S, et al. Extralevator with *vs* nonextralevator abdominoperineal excision for rectal cancer: the RELAP e randomized controlled trial. Colorectal Dis. 2017 Feb;19(2):148–57.

29. Cesar D, Araujo R, Valadão M, Linhares E, Meton F, Jesus JPD. Surgical and oncological short-term outcomes of prone extralevator abdominoperineal excision for low rectal cancer. J Coloproctology. 2018 Jun;38(02):124–31.

30. Colov EP, Klein M, Gögenur I. Wound Complications and Perineal Pain After Extralevator Versus Standard Abdominoperineal Excision: A Nationwide Study. Dis Colon Rectum. 2016 Sep;59(9):813–21.

31. Danilov MA, Leontyev AV, Baychorov AB, Abdulatipova ZM, Saakyan GG. Laparoscopic extralevator abdominoperineal extirpation of the rectum: long-term results. Pelvic Surg Oncol. 2020 Dec 30;10(3–4):34–42.

32. Dsouza R, Varghese G, Mittal R, Jesudason MR. Perineal Wound Outcomes after Extralevator Abdominoperineal Excision for Rectal Adenocarcinoma- A Tertiary Care Centre Experience. Wound Med. 2020 Jun;29:100184.

33. Güven HE, Aksel B. Is extralevator abdominoperineal resection necessary for low rectal carcinoma in the neoadjuvant chemoradiotherapy era? Acta Chir Belg. 2020 Sep 2;120(5):334–40.

34. Han JGang, Wang ZJun, Wei GHui, Gao ZGang, Yang Y, Zhao BCheng. Randomized clinical trial of conventional versus cylindrical abdominoperineal resection for locally advanced lower rectal cancer. Am J Surg. 2012 Sep;204(3):274–82.

35. Hanif Z, Bradley A, Hammad A, Mukherjee A. Extralevator abdominoperineal excision (Elape): A retrospective cohort study. Ann Med Surg. 2016 Sep;10:32–5.

36. How P, West NP, Brown G. An MRI-based Assessment of Standard and Extralevator Abdominoperineal Excision Specimens: Time for a Patient Tailored Approach? Ann Surg Oncol. 2014 Mar;21(3):822–8.

37. Kamali D, Sharpe A, Musbahi A, Reddy A. Oncological and quality of life outcomes following extralevator versus standard abdominoperineal excision for rectal cancer. Ann R Coll Surg Engl. 2017 May;99(5):402–9.

38. Klein M, Fischer A, Rosenberg J, Gögenur I. ExtraLevatory AbdominoPerineal Excision (ELAPE) Does Not Result in Reduced Rate of Tumor Perforation or Rate of Positive Circumferential Resection Margin: A Nationwide Database Study. Ann Surg. 2015 May;261(5):933–8.

39. Liu B, Farquharson J. The quality of lymph node harvests in extralevator abdominoperineal excisions. BMC Surg. 2020 Dec;20(1):241.

40. Nessar G, Demirbag AE, Celep B, Elbir OH, Kayaalp C. Extralevator abdominoperineal excision versus conventional surgery for low rectal cancer: a single surgeon experience. Turk J Surg. 2016 Dec 21;32(4):244–7.

41. Papp G, Dede K, Bursics A. Short-term advantages of ELAPE over APR. Acta Chir Belg. 2021 Sep 3;121(5):327–32.

42. Park S, Hur H, Min BS, Kim NK. Short-term Outcomes of an Extralevator Abdominoperineal Resection in the Prone Position Compared With a Conventional Abdominoperineal Resection for Advanced Low Rectal Cancer: The Early Experience at a Single Institution. Ann Coloproctology. 2016;32(1):12.

43. Perdawood SK, Lund T. Extralevator versus standard abdominoperineal excision for rectal cancer. Tech Coloproctology. 2015 Mar;19(3):145–52.

44. Prytz M, Angenete E, Ekelund J, Haglind E. Extralevator abdominoperineal excision (ELAPE) for rectal cancer—short-term results from the Swedish Colorectal Cancer Registry. Selective use of ELAPE warranted. Int J Colorectal Dis. 2014 Aug;29(8):981–7.

45. Qi X, Liu M, Tan F, Xu K, Yao Z, Zhang N, et al. Laparoscopic extralevator abdominoperineal resection versus laparoscopic abdominoperineal resection for lower rectal cancer: A retrospective comparative study from China. Int J Surg. 2019 Nov;71:158–65.

46. Shen Z, Bu Z, Li A, Lu J, Zhu L, Chong CS, et al. Multicenter study of surgical and oncologic outcomes of extra-levator versus conventional abdominoperineal excision for lower rectal cancer. Eur J Surg Oncol. 2020 Jan;46(1):115–22.

47. Stelzner S, Hellmich G, Schubert C, Puffer E, Haroske G, Witzigmann H. Short-term outcome of extra-levator abdominoperineal excision for rectal cancer. Int J Colorectal Dis. 2011 Jul;26(7):919–25.

48. Stelzner S, Hellmich G, Sims A, Kittner T, Puffer E, Zimmer J, et al. Long-term outcome of extralevator abdominoperineal excision (ELAPE) for low rectal cancer. Int J Colorectal Dis. 2016 Oct;31(10):1729–37.

49. Ulusoy C, Nikolovski A. Extralevator Abdominoperineal Excision (ELAPE) is Not Superior to Abdominoperineal Excision (APE) in the Era of Neoadjuvant Treatment in Rectal Cancer. PRILOZI. 2022 Nov 1;43(3):21–7.

50. Van Oostendorp SE, Roodbeen SX, Chen CC, Caycedo-Marulanda A, Joshi HM, Tanis PJ, et al. Transperineal minimally invasive APE: preliminary outcomes in a multicenter cohort. Tech Coloproctology. 2020 Aug;24(8):823–31.

51. Wang Z, Liang R, Yalikun D, Yang J, Li W, Kou Z. Laparoscopic extralevator abdominoperineal excision in distal rectal cancer patients: a retrospective comparative study. BMC Surg. 2022 Dec 8;22(1):418.

52. West NP, Anderin C, Smith KJE, Holm T, Quirke P. Multicentre experience with extralevator abdominoperineal excision for low rectal cancer. Br J Surg. 2010 Mar 4;97(4):588–99.

53. Zhang H, Li G, Cao K, Zhai Z, Wei G, Ye C, et al. Long-term outcomes after extra-levator versus conventional abdominoperineal excision for low rectal cancer. BMC Surg. 2022 Dec;22(1):242.
